# Deconstructing α‑Amidoalkyl Sulfones as Dual *d*‑Sulfonyl/*a*‑Azomethine Synthons: Synthesis of 3‑Sulfonylmethylindole Aminals

**DOI:** 10.1021/acs.joc.5c01392

**Published:** 2025-08-07

**Authors:** Guillermo Domínguez, Anje Mujika, Iker Hernández, Vadim A. Soloshonok, Aitor Landa, Mikel Oiarbide

**Affiliations:** † Department of Organic Chemistry I, Faculty of Chemistry, University of the Basque Country UPV/EHU, 20018 Donostia/San Sebastián, Spain; ‡ Basque Foundation for Science, IKERBASQUE, Bilbao 48013, Spain

## Abstract

α-Amido sulfones (**1**) are widely applied as electrophilic aminoalkylation reagents. However, nonproductive sulfinate species are formed alongside. Here, we report that ethyl propiolate-promoted coupling of **1** and gramines provides 3-sulfonylmethylindole aminals **II** smoothly, thus establishing α-amido sulfones as dual donor/acceptor reagents with full atom incorporation on the target molecule. Simple adjustment of reactants loading allows one to revert the reaction outcome, leading to exclusive formation of 3-sulfonylmethylindoles **I** instead.

## Graphical abstract

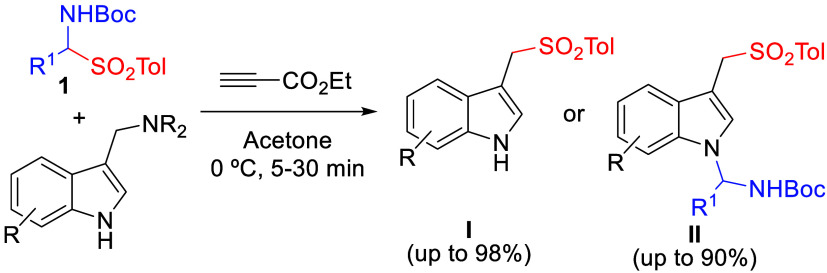

## Introduction

α-Amido sulfones (**1**, R^2^ = aryl or OR′, Figure 1A) are readily accessible reagents from the three-component coupling of an amide (or carbamate), an aldehyde, and the corresponding organosulfinic acid or salt.^1^ Compounds **1**, which tend to be stable solids, have been widely applied as the synthetic equivalent of the electrophilic azomethine synthon **2**.^2^ Bases can promote the release of **2** from **1** for subsequent *in situ* coupling with a variety of nucleophiles. This tactic has been successfully implemented for aminoalkylations of carbon- and heteroatom-centered nucleophiles,^2,3^ including catalytic enantioselective variants.^4^ In all of these developments, the aryl­(alkyl)­sulfinyl fragment **5** does not get incorporated onto the target molecule and becomes waste material. Rare exceptions to this established behavior are known in which reagent **1** may serve as a synthetic equivalent of arenesulfinyl anion **3** and the aryl­(alkyl)­sulfonylmethyl cation **4**, respectively. Thus, Petrini found^5^ that the Friedel–Crafts reaction of indoles with α-amido sulfones in the presence of montmorillonite K-10 leads unexpectedly to 3-(1-arylsulfonylalkyl) indoles in good yield, while Yan reported^6^ the organocatalyzed conjugate addition of **1** to β-aryl ynones and ynoates to furnish axially chiral α-sulfonyl styrenes enantioselectively. Once again, these two examples represent synthetic applications, wherein fragments of reagent **1** (amide moiety **7** and *N*-acylimine moiety **6**, respectively) are sacrificially lost through nonproductive pathways.

**Figure 1:**
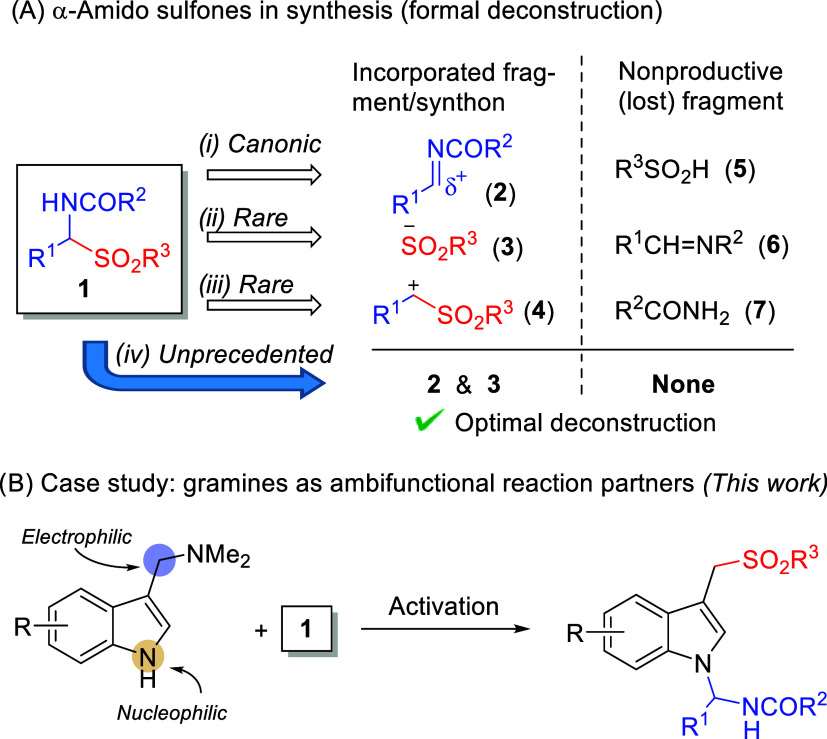
α-Amido sulfones as ambifunctional reagents.

The possibility of fully exploiting the rich functionality of α-amido sulfone reagents **1** by incorporating all their constituents onto the reaction product in a single synthetic operation, while tempting, remains unmet so far. Since deconstruction of **1** would deliver two fragments possessing, respectively, electron-acceptor and donor character, the idea of pairing reagent **1** with a complementary donor/acceptor ambifunctional reaction partner seemed attractive. However, such a reactant combination poses significant selectivity issues, as several crossed and self-condensation pathways may compete. In addition, under nonspecific activation mechanisms, *e.g*. general base- or acid-activation approaches, total or partial quenching of the required dual reactivity may occur, ultimately leading to low reaction efficiency and/or selectivity. In this context, we thought of gramines as potentially ambifunctional reaction partner candidates. Gramines display a nucleophilic N_1_ nitrogen, while the carbon attached at C_3_ of the indole ring may become electrophilic upon proper C_1′_–N bond activation. In particular, early studies by Sainsbury and further refinement by Williams uncovered mild activation of the gramines C_1′_–NMe_2_ bond via nitrogen quaternization through conjugate addition to a propiolic ester, thus enabling coupling with certain active methylenes.^7^ Given the acid/base- and redox-neutral nature of this unique activation mechanism, we presumed that such activation conditions would be compatible with the reactivity of α-amido sulfones **1**. Here, we report that, indeed, α-amido sulfones **1** may behave as dual donor/acceptor reagents in the ethyl propiolate-promoted coupling with gramines **8** to efficiently furnish 3-sulfonylmethylindole aminals **14**.^8,9^ Interestingly, it was also found that by adjusting the mol ratio of reactants, exclusive formation of either the dual addition product **14** or the monoaddition product **13** ^10,11^ takes place in a fully controllable manner.

## Results and Discussion

The model reaction between gramine **8a** and α-amido sulfone **1a** was selected for initial exploration using ethyl propiolate **12** as the only reaction promoter. In a first trial, stirring an equimolar mixture of the triad **1a**/**8a**/**12** in CH_2_Cl_2_ at 0 °C for 16 h was accompanied by the disappearance of most starting material (85% conv.) and formation of a mixture of mono- and difunctionalized adducts **13aa** and **14aa** in a 1:3 ratio (Table 1, entry 1). Further exploration showed that the reaction outcome strongly depends on the molar ratio of the reactants. Thus, slightly decreasing the amount of gramine **8a** and promoter **12** with respect to α-amido sulfone **1a** caused a reversal in the products distribution. For instance, using **1a**/**8a**/**12** in a ratio of 1:0.8:0.8, monofunctionalized product **13aa** was obtained almost exclusively (86% conversion, entry 2). Further screening of the conditions established an optimal reactants ratio of 1:0.5:0.8, leading to 90% reaction conversion from which essentially pure compound **13aa** could be isolated in 80% yield (entry 3). Conversely, increasing the ratio of **8a** and **12** with respect to α-amido sulfone **1a**, under otherwise identical conditions, led to a dramatic increase in selectivity while favoring formation of adduct **14aa**. Thus, applying a ratio of the three reactants of 1:1.2:1.6 allowed to obtain **14aa** almost exclusively (**13aa**/**14aa**= 1:>20) and in 80% isolated yield (entry 9). Further increases of the relative amounts of both **8a** and **12** were fruitless (entry 10). Then, it was observed that the reaction proceeded in chloroform, ethyl acetate, and acetonitrile faster and more efficiently than in dichloromethane (entries 4–6 vs 3). The optimum solvent turned out to be acetone (entry 7), in which essentially complete conversion was achieved in just a few minutes, leading to almost quantitative formation of adduct **13aa**. Ethanol was less suitable as a solvent (entry 8). Acetone was also the best performing solvent for the double addition reaction (entry 11), so conditions for entries 7 and 11 were selected for further reaction development. Finally, three additional gramines (**9–11**) were also tested under the above optimized conditions. While the reactions involving pyrrolidine-derived gramine **9** and piperidine-derived gramine **10** proceeded smoothly, furnishing sulfonylated product **13aa** in yields of 94 and 96%, respectively, the morpholine-derived gramine **11** proved to be comparatively less reactive (see Table SI-1 for details). It should be noted that in neither of the above reactions, a product from sulfonylation of acetylenic ester **12** was observed.^12^

**Table 1:**
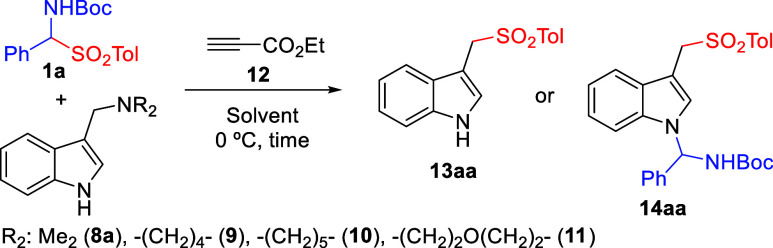
Screening of Conditions for the Ethyl Propiolate-Promoted Coupling of Gramines and α-Amido Sulfone 1a

entry	**1a:8a:12** (mol ratio)	solvent	time (h)	conv. (%)^b^	**13aa:14aa** (mol ratio)^b^ ^,^ ^c^
1	1:1.0:1.0	CH_2_Cl_2_	16	85	1:3
2	1:0.8:0.8	CH_2_Cl_2_	16	86	>20:1
3	1:0.5:0.8	CH_2_Cl_2_	16	90	>20:1 (80%)
4	as above	CHCl_3_	0.5	95	>20:1 (91%)
5	as above	EtOAc	0.5	96	>20:1 (94%)
6	as above	CH_3_CN	0.5	94	>20:1 (90%)
7	**as above**	**acetone**	**0.5**	**99**	>20:1 **(98%)**
8	as above	EtOH	0.5	60	>20:1 (54%)
9	1:1.2:1.6	CH_2_Cl_2_	16	92	1:>20 (80%)
10	1:2.0:2.0	CH_2_Cl_2_	16	80	1:>20
11	1:1.2:1.6	**acetone**	**0.5**	**99**	1:>20 (86%)

a To a solution of **1a** and **12**, gramine **8a** dissolved in the specified solvent was added dropwise and the mixture stirred at 0 °C.

b Determined by ^1^H NMR.

c In parentheses, the yield of isolated major product after purification.

The reaction leading to sulfonylated adducts **13** under the conditions of Table 1, entry 7, proved to be applicable to a wide range of gramines featuring variable substitution patterns. For example (Scheme 1), gramines bearing 5-methyl, 5-methoxy, and 6-benzyloxy groups at the indole ring provided adducts **13ab**, **13ac**, and **13ad** in yields of 90, 91, and 86%, respectively. Halogen substituents at different positions of the indole ring were well tolerated too, as illustrated by the formation of chloro-substituted adducts **13ae–ah** in 81, 89, 91, and 90% yield, and the fluorinated products **13ai** and **13aj** in 85 and 92% yield, respectively. Other electron-poor gramines, such as the 5-trifluoromethyl and 5-methoxycarbonyl gramines **8k**,**l** led to the corresponding adducts **13ak** and **13al** with yields of 79 and 88%. The cyano- and nitro-substituted gramines showed differentiated reactivity. Thus, both 5- and 6-cyano gramines **8m**,**n** showed high reaction rates and the corresponding adducts **13am**,**an** could be isolated in good yields, provided the reactions are kept for just 5 min and then directly submitted to column chromatography; the reaction with 6-nitro gramine **8o** was so fast that difunctionalized product **14ao** was invariably obtained (see below). The boryl-substituted gramine **8p** furnished sulfonylated adduct **13ap** in a good isolated yield (93%). Importantly, the present method is also applicable to 7-aza analogue **15** as well as 2-methyl and 2-phenyl substituted gramines **8r** and **8s**, which led, respectively, to products **16aq**, **13ar**, and **13as** in yields of 84, 82, and 84%. Remarkably, the reaction also proceeded smoothly, with the *N*-methyl counterpart of **8a** affording adduct **13at** in 71% yield. Larger-scale coupling reaction between **8a** (11 mmol) and **1a** afforded 2.83 g of adduct **13aa** (90% yield).

**Scheme 1:**
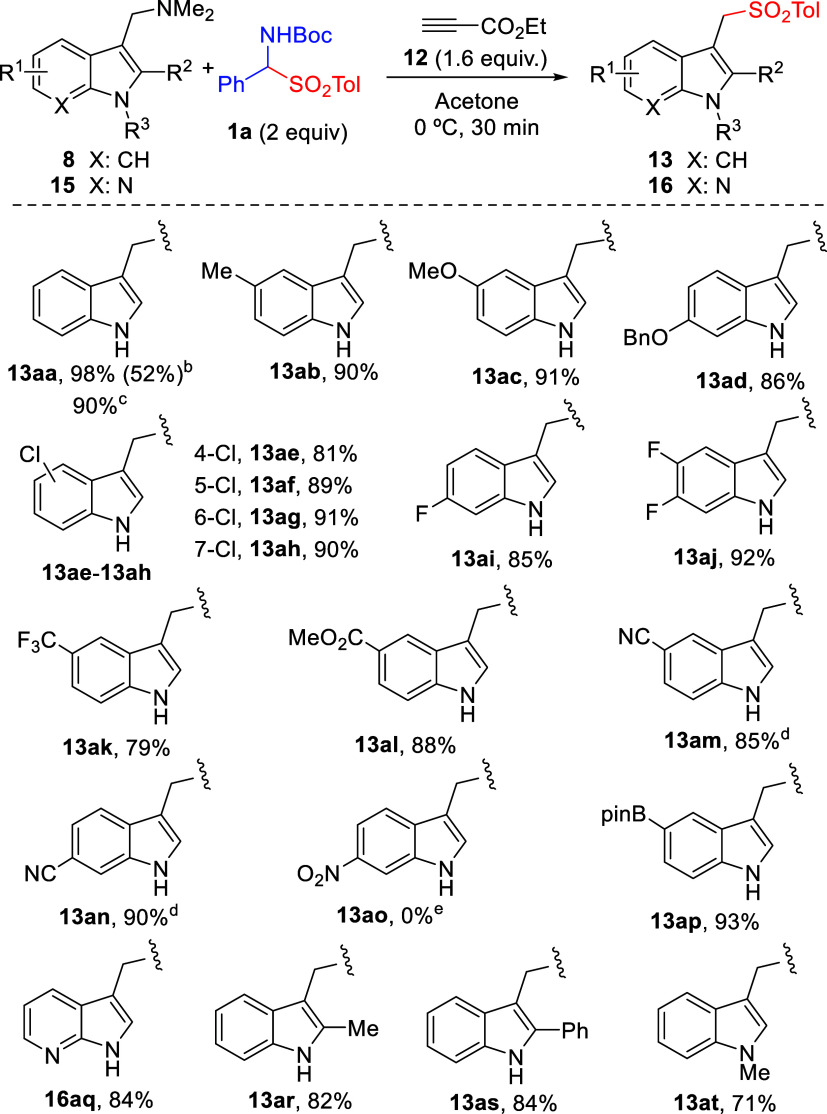
Scope of the Reaction Leading to 3-Arenesulfonylmethyl (aza)­indoles 13/16^e^

a Reactions conducted at 0.2 mmol scale. Yields correspond to isolated **13**/**16** after purification.
b Using *N*-Cbz analogue **1a**′.
c Reaction run at 11 mmol scale.
d Reaction stirred for 5 min only, otherwise double addition product appears.
e Double addition product **14ao** is formed instead (57% yield).

Then, we investigated the generality of the double functionalization of gramines that involves α-amido sulfones **1** as dual reagents. It was encouraging to observe that using the parent gramine **8a** under the optimized conditions of Table 1, entry 11, α-amido sulfones **1e** and **1f**, bearing the 4*-*chlorophenyl and 4*-*methoxyphenyl residue, worked well affording sulfonylated aminals **14ea** and **14fa** in yields of 83 and 84%. α-Amido sulfones derived from aliphatic aldehyde propanal (**1b**) and even formaldehyde (**1d**) led to adducts **14ba** and **14da** in slightly lower, yet synthetically useful, isolated yields (72 and 61%). The successful coupling of gramine **8a** and α-amido sulfone **1c** to furnish adduct **14ca** in 72% yield further indicates that α-amido sulfones derived from aliphatic aldehydes are well-tolerated and that both tolyl- and phenylsulfonyl groups can be equally compatible. Then, variations on the gramine side were evaluated. The coupling of **1a** with methyl, methoxy-, and benzyloxy-substituted gramines **8b**–**d** afforded aminals **14ab**, **14ac**, and **14ad** in yields of 84, 82, and 88%, respectively. Halogenated gramines, such as 4-, 5-, and 6-chlorogramines **8e**–**g**, and 5,6-difluorogramine **8j**, furnished the corresponding aminals **14ae**–**ag** and **14aj** in good yields too. However, 7-chlorogramine **8h** did not react under these conditions to provide **14ah**, probably because of steric repulsion between the substituent at C7 and the incoming azomethine becomes incompatible with the formation of the corresponding transition state (*vide infra*). Indeed, when the less demanding α-unsubstituted amido sulfone **1d** was employed, aminal **14dh** was isolated in 80% yield. Similarly, while the reaction of 2-substituted gramine **8s** with **1a** did not proceed, the coupling with α-unsubstituted **1d** proceeded smoothly, affording **14ds** in 77% yield. Gramines bearing nitrile (**8m**, **8n**), nitro (**8o**), as well as ester **8l** did also participate satisfactorily. Once again, the larger-scale coupling reactions of gramine **8a** with α-amido sulfone **1a**, and of gramine **8s** with α-amido sulfone **1d** (both run at 6 mmol), were easy to perform, affording products **14aa** and **14ds** in isolated yields of 2.3 g (78%) and 2.53 g (86%), respectively (Scheme 2).

**Scheme 2:**
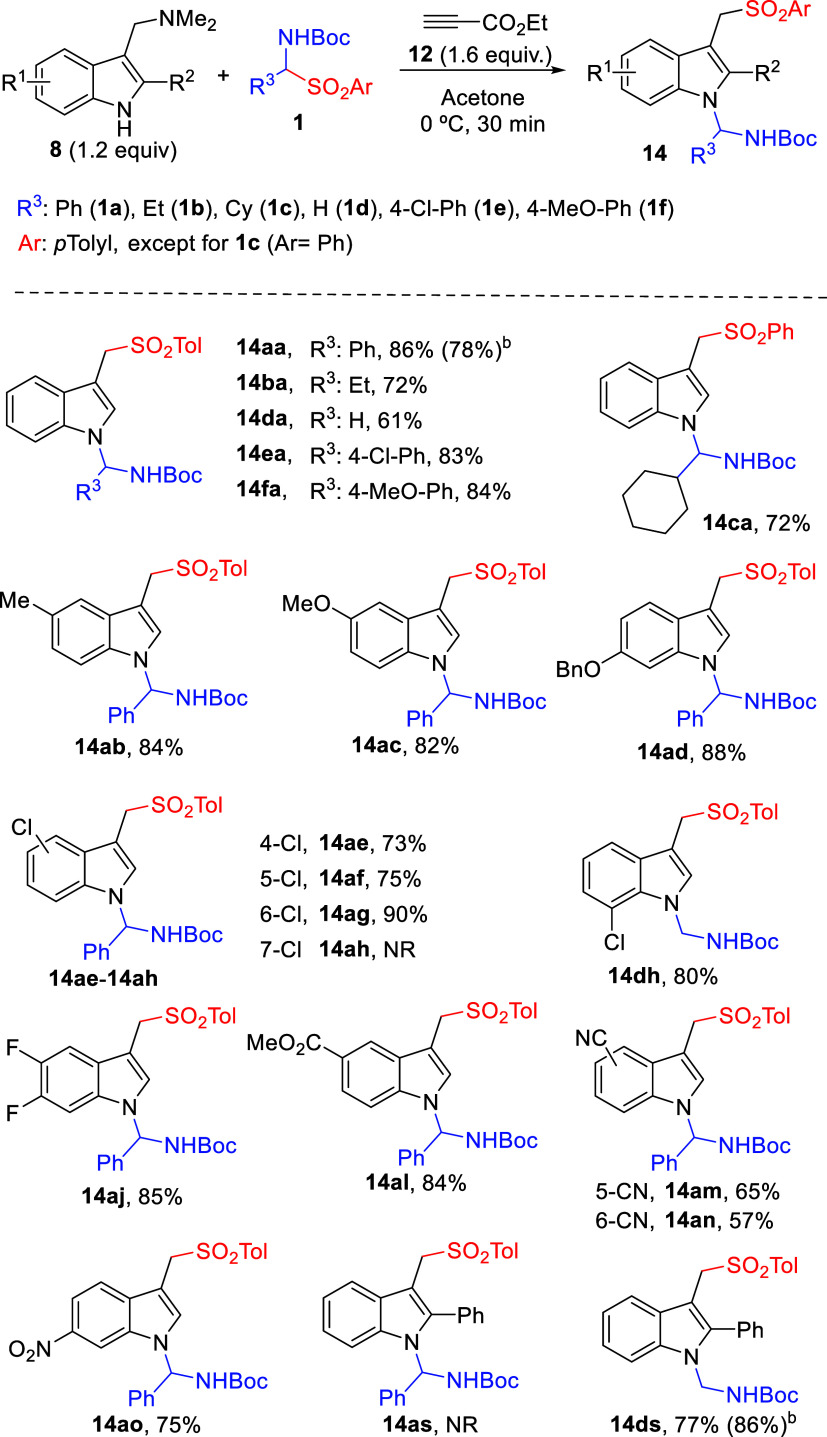
α-Amido Sulfones as Dual Donor/Acceptor Reagents^b^

a Reaction conducted at 0.2 mmol scale. Yields correspond to isolated **14** after purification.
b Reaction run at 6 mmol scale.

In order to rationalize the role played by each reaction component during aminal formation, the reactivity of sulfonylated adduct **13aa** and *N*-Boc imine **17**, the most plausible immediate precursors, was studied under specially designed conditions. As Scheme 3 shows, in the absence of any “additive” both molecules remained intact (entry a). As expected, addition of gramine **8a** (1 equiv) restored reactivity and aminal **14aa** was obtained along with minor amounts of **21** resulting from the competing reaction of **17** with gramine (entry b). Addition of Et_3_N or alternative tertiary amines, including chiral ones, had no impact, and unreacted materials were recovered (entry c). Most interestingly, neither *N*-methyl analogue **18** nor 3-methylindole **19** were able to promote the reaction (entries d and e), indicating both NH and NMe_2_ functionalities of gramine apparently work in cooperation. Moreover, since a combination of both **18** and **19** also failed in promoting the coupling (entry f), it may be inferred that both functionalities must be linked covalently as in gramines for cooperative action being effective. Additional experiments (not shown) revealed that gramine **8a** is able to react directly with **17** to afford aminal **21**, whereas 3-methylindole **19** did not, further supporting the idea of bifunctional activation and pointing toward intermolecular hydrogen bonds as the key player. However, Takemoto’s thiourea catalyst **C1** was unable to promote the reaction between **13aa** and **17** (entry g). It was intriguing that when employed in combination with gramine **8a** as an additive, this catalyst did not affect the gramine-promoted coupling between **17** and **13aa** to any significant extent, but instead fully inhibited competitive formation of aminal **21** (entry h vs b). Finally, upon addition of one equivalent of enamine **20**, the presuming side product during sulfonylation step, aminoalkylated product **22** was formed (entry (i)). It should be noted that neither product **21** nor **22** were detected under optimal reaction conditions developed in Table 1 and Scheme 2, thus indicating neither reaction pathway toward these compounds is mainstream under standard conditions. Looking for evidence of any relevant intermolecular hydrogen-bond interactions involving gramines **8** and 3-sulfonylmethylindoles **13**, we analyzed the ^1^H NMR chemical shift variations of indolic NH signals as a function of temperature for both **8a** and **13aa**, independently, taken in DMSO-*d*_6_ as the solvent (Figure 2a,b).^13^ Representation of this variation afforded straight lines with slopes of −3.7 and −2.9 ppb/K for compounds **8a** and **13aa** alone, respectively. These values are consistent with both compounds forming H-bonded aggregates in solution. When an equimolar mixture of **8a** and **13aa** was submitted to the same monitoring (Figure 2c), the corresponding straight line slope for each NH peak was −2.9 and −2.4; that is, 0.8 and 0.5 higher relative to **8a** and **13aa** taken alone. This observation suggests that the H-bond network in the heteroaggregate (**8a**:**13aa**) is stronger than that in the corresponding homoaggregates.

**Scheme 3:**
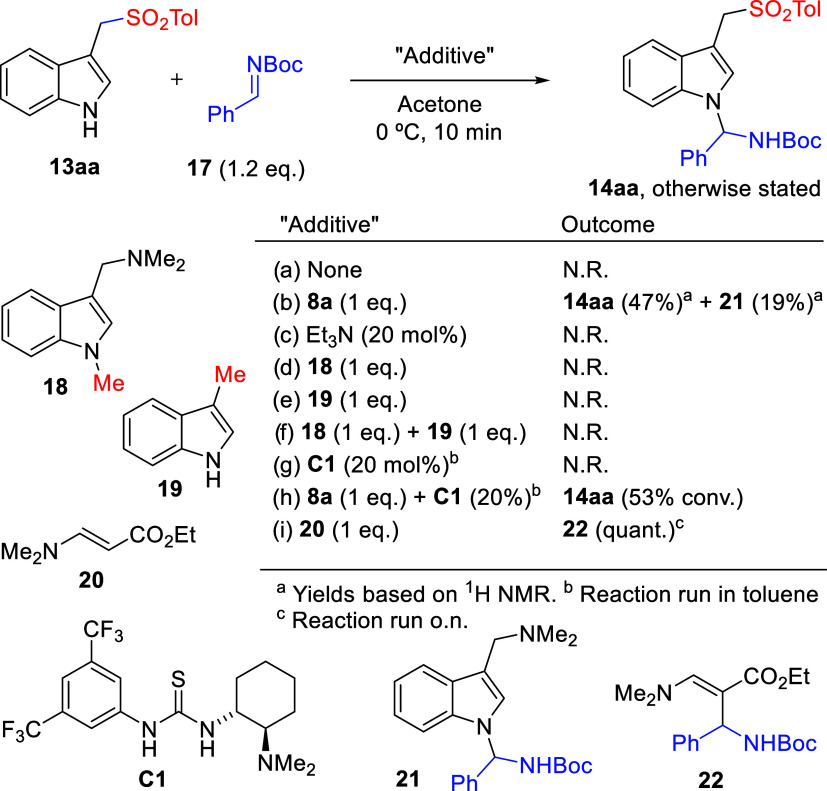
Control Experiments

**Figure 2:**
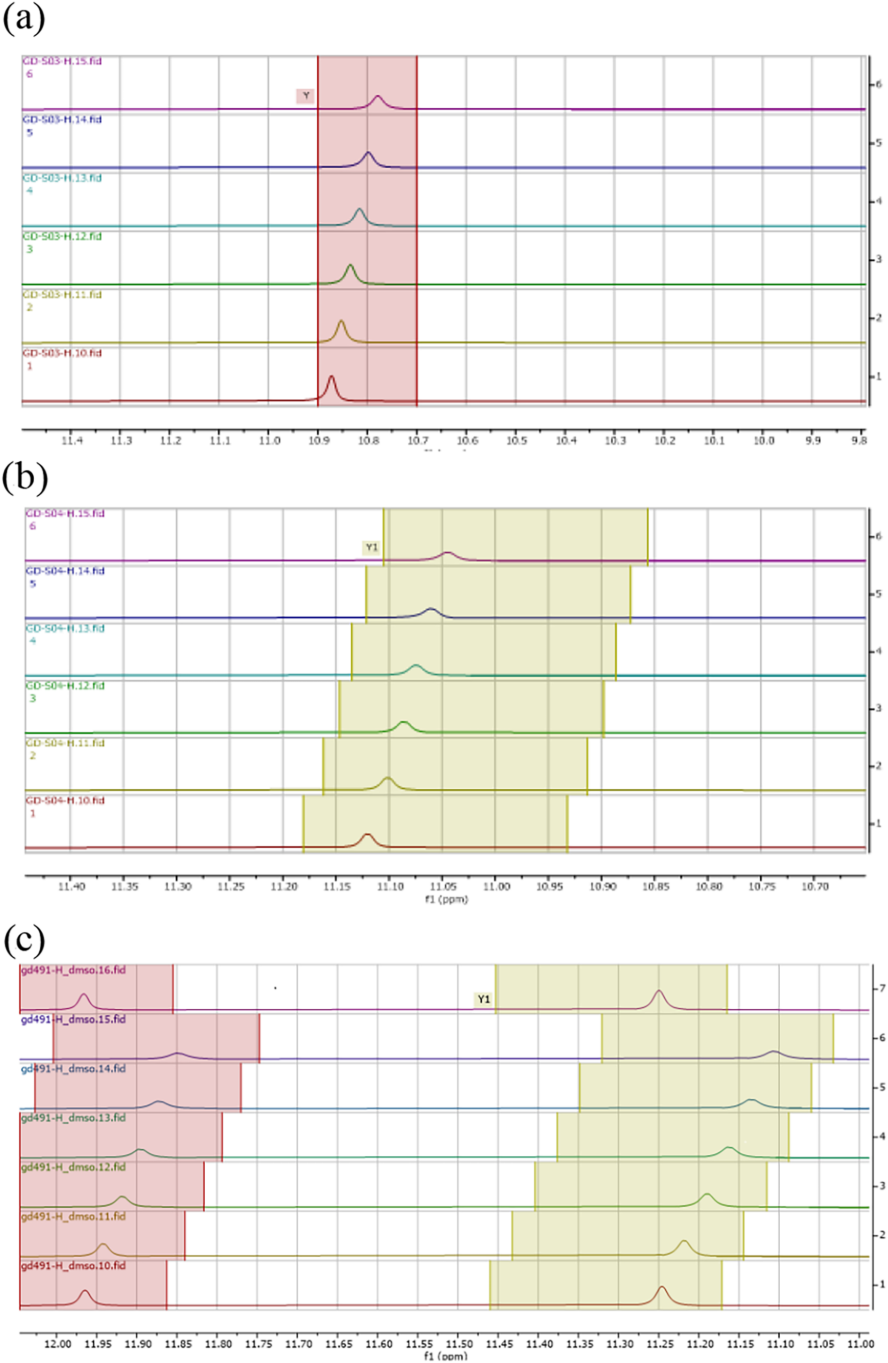
Variation of ^1^H NMR *NH* peaks as a function of temperature for (a) gramine **8a** alone, (b) sulfone **13aa** alone, and (c) equimolar admix of **8a** and **13aa** (taken in DMSO-*d*_6_; Δ*T* intervals = 5 K). Equations of δ (ppm) vs Δ*T* straight lines: (a) δ = 11.98–0.003721Δ*T*; (b) δ = 11.99–0.002936Δ*T*; and (c) δ_1_ = 12.09–0.00289296Δ*T*, δ_2_ = 12.67–0.00243207Δ*T*.

Considering the collective data above, the pathway depicted in Scheme 4 would explain the formation of both the mono- and dual addition products in a sequential manner. On the one hand, base-assisted fragmentation of α-amido sulfone **1a** into *N*-Boc imine **17** and arylsulfonyl anion **3** would take place. As we confirmed by independent control experiments (see Supporting Information), gramine **8a** would be able to promote such fragmentation, but participation of other basic species that evolve along the process could not be discarded. In parallel, the propiolate ester **12**-promoted deaminative generation of azafulvene-like intermediate **I-2** from gramine **8a** would take place. Then, the addition reaction of arylsulfonyl anion **3** to electrophilic **I-2** and subsequent protonation would furnish sulfonylated adduct **13aa**. According to the short reaction times observed experimentally for the formation of isolable adducts **13** (almost complete conversion after roughly 10 min of reaction at 0 °C), all of the above elemental steps would be fast and essentially irreversible. Then, the formation of aminal **14** would occur by the addition of **13aa** to *N*-Boc imine **17**, very likely with the remaining gramine **8a** playing a critical activation role. While highly speculative, we tentatively propose that this reaction may proceed through **TS-1**, a transition-state model in which both reactants and gramine **8a** interact through H-bonding. An alternative pathway would involve the nucleophilic indole nitrogen generated upon addition of the tolylsulfinate anion **3** to the vinylogous intermediate **I-2**, which would directly react with acylimine **17**. However, this unassisted pathway would not explain the necessity of having an excess of gramine **8** for the reaction to proceed to aminal product **14**. In addition, this alternative pathway would hardly explain the observed different rates with which monoaddition adducts **13** (fast formation) and dual addition adducts **14** (comparatively slower formation) are formed. Moreover, the allegedly generated anionic indole intermediate would be strongly basic and would expectedly be protonated by the R_3_NH^+^ generated in the previous step.

**Scheme 4:**
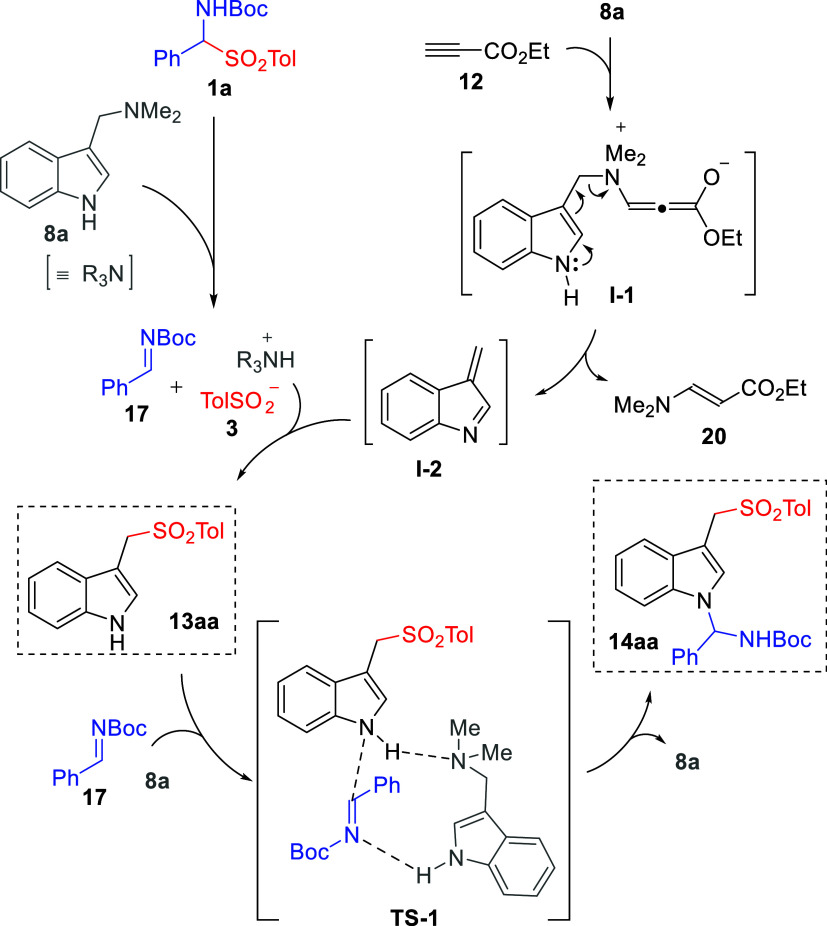
Plausible Reaction Pathway

## Conclusions

In conclusion, we demonstrate that the reaction between gramines **8** and α-amido sulfones **1** in the presence of ethyl propiolate may furnish sulfonylated adducts **13** or, alternatively, double addition aminals **14**, in a controllable fashion. Experimental evidence shows that the presence of unreacted gramine **8** is critical for the aminal formation step to proceed efficiently and that crossed H-bonding interactions between adduct **13** and gramine **8** are likely involved. To our knowledge, this novel approach to aminals **14** represents the first application of α-amido sulfones **1** as dual (donor/acceptor) sulfonyl/azomethine synthons. We hope these results will stimulate the exploration of this dual synthon concept to other reaction settings.

## Experimental Section

### General Information

All nonaqueous reactions were performed under an inert atmosphere using oven-dried glassware and were magnetically stirred. Reactions requiring low temperatures were performed using Julabo FT902 cooling bath circulators and isopropanol baths. Yields refer to chromatographically purified samples unless otherwise stated. Organic layers washed with aqueous phases were dried over MgSO_4_ and evaporated under reduced pressure using rotary evaporators Büchi R-200 and R-210. For trace solvent removal, vacuum pump Edwards nXDS6iC (≈0.5 mmHg) was employed. Column chromatography was performed on ROCC 60 silica gel 40–63 μm as stationary phase and a suitable mixture of solvents (typically hexane/ethyl acetate) as eluent. Thin layer chromatography (TLC) was conducted using Merck silica gel 60 F254 plates, and spots were visualized by fluorescence quenching under UV light and by staining the plates with a dipping aqueous solution of potassium permanganate (1 g of salt in 100 mL of water), followed by heating. Melting points were determined in open capillaries in a Stuart SHP3 melting point apparatus. ^1^H NMR and ^13^C NMR spectra were recorded at 300 and 75 MHz, respectively. The chemical shifts are reported in ppm relative to CDCl_3_ (δ = 7.26), DMSO-*d*_6_ (δ = 2.50), methanol-*d*_4_ (δ = 3.31), or acetone-*d*_6_ (δ = 2.05) for ^1^H NMR and relative to the central resonances of CDCl_3_ (δ = 77.2), DMSO-*d*_6_ (δ = 39.5), or acetone-*d*_6_ (δ = 29.8) for ^13^C NMR. Peaks are labeled as singlet (s), doublet (d), triplet (t), quartet (q), double doublet (dd), double triplet (dt), triplets of doublets (td), quartets of doublets (qd), or multiplet (m). Coupling constants (*J*) are reported in Hertz (Hz). *MestReNova Mnova* 11.0.4 program was employed to process and edit the registered spectra. Mass spectra were recorded on an ESI-ion trap mass spectrometer (Agilent 1100 series LC/MSD, SL model) on a UPLC-DAD-QTOF, Ultra High Performance Liquid Chromatography–Mass spectrometer, Waters UPLC ACQUITY, Waters PDA detector, Waters Sunapt G2 or Agilent Thermoquest LCT spectrometer. Infrared spectra were measured employing a Bruker α-P compact FT-IR spectrometer.

All reagents, including indoles, carbamates, and aldehydes, were purchased from commercial suppliers and used without further purification. α-Amido sulfones **1**, gramines **8–11**, and the 7-aza analogue **15** were prepared according to literature procedures as detailed in the Supporting Information. Et_3_N was purified by distillation over KOH. Anhydrous solvents were obtained following the established procedures: CH_2_Cl_2_ and CH_3_CN were dried over CaH_2_. HPLC-grade toluene, acetone, EtOAc, and EtOH were used as purchased.

### General Procedure for the Coupling between Gramines 8 (or the 7-aza Analogue 15) and α-Amido Sulfones 1—Method A: Synthesis of Products 13/16 (Scheme 1)

To a solution of the corresponding gramine **8** or **15** (0.2 mmol, 1.0 equiv) in acetone (2 mL), α-amido sulfone **1a** (144 mg, 0.4 mmol, 2.0 equiv) was added, and after the reaction was cooled to 0 °C, ethyl propiolate **12** (31.4 mg, 0.32 mmol, 1.6 equiv) was added dropwise. After stirring the mixture for 30 min, the solvent was evaporated and the crude product was purified by flash column chromatography using as eluent a gradient mixture of hexane/ethyl acetate. The resulting material was finally washed with *n*-hexane and dried under vacuum.

#### 3-(Tosylmethyl)-1*H*-indole (**13aa**)

The title compound was prepared from gramine **8a** (34.8 mg, 0.2 mmol, 1 equiv) according to the general procedure. Column chromatography performed using gradient elution: 10:1 to 5:1 hexane/ethyl acetate. White solid. Yield: 56 mg, 0.196 mmol, 98%. ^1^H NMR (300 MHz, CDCl_3_) δ 8.18 (s, 1H), 7.55 (d, *J* = 8.2 Hz, 2H), 7.36–7.27 (m, 2H), 7.16 (dd, *J* = 7.7, 4.8 Hz, 3H), 7.09 (d, *J* = 2.6 Hz, 1H), 7.06–6.99 (m, 1H), 4.52 (s, 2H), 2.37 (s, 3H). All spectroscopy data were coincident with those previously reported in the literature.^10f^

(**Reaction run at 11 mmol scale**) To a solution of gramine **8a** (1.92 g, 11 mmol, 1 equiv) in acetone (10 mL), α-amido sulfone **1a** (7.95 g, 22 mmol, 2 equiv) was added and, after cooling the reaction to 0 °C, ethyl propiolate **12** (1.73 g, 17.6 mmol, 1.6 equiv) was added dropwise. After stirring the mixture for 30 min at the same temperature, the solvent was evaporated and the crude product was purified by flash column chromatography (gradient elution: 10:1 to 5:1 hexane/ethyl acetate). The resulting material was washed with *n*-hexane and dried under vacuum to yield essentially pure **13aa** as a white solid: 2.83 g (9.9 mmol, 90%).

#### 5-Methyl-3-(tosylmethyl)-1*H*-indole (**13ab**)

The title compound was prepared from gramine **8b** (37.6 mg, 0.2 mmol, 1 equiv) according to the general procedure. Column chromatography performed using gradient elution: 10:1 to 5:1 hexane/ethyl acetate. White solid. Yield: 53.8 mg, 0.180 mmol, 90%. ^1^H NMR (300 MHz, CDCl_3_) δ 8.16 (s, 1H), 7.59–7.44 (m, 2H), 7.29–7.22 (m, 1H), 7.22–7.14 (m, 2H), 7.04 (d, *J* = 2.7 Hz, 1H), 6.97 (d, *J* = 8.4 Hz, 1H), 6.93 (d, *J* = 3.7 Hz, 1H), 4.49 (d, *J* = 3.0 Hz, 2H), 2.39 (d, *J* = 2.8 Hz, 3H), 2.33 (d, *J* = 3.0 Hz, 3H). All spectroscopy data were coincident with those previously reported in the literature.^10e^

#### 5-Methoxy-3-(tosylmethyl)-1*H*-indole (**13ac**)

The title compound was prepared from gramine **8c** (40.8 mg, 0.2 mmol, 1 equiv) according to the general procedure. Column chromatography performed using gradient elution: 10:1 to 5:1 hexane/ethyl acetate. White solid. Yield: 57.4 mg, 0.182 mmol, 91%. ^1^H NMR (300 MHz, CDCl_3_) δ 8.24 (s, 1H), 7.61–7.47 (m, 2H), 7.23–7.11 (m, 3H), 7.02 (d, *J* = 2.7 Hz, 1H), 6.80 (dd, *J* = 8.8, 2.4 Hz, 1H), 6.63 (d, *J* = 2.4 Hz, 1H), 4.55–4.38 (m, 2H), 3.72 (s, 3H), 2.38 (s, 3H). All spectroscopy data were coincident with those previously reported in the literature.^10e^

#### 6-(Benzyloxy)-3-(tosylmethyl)-1*H*-indole (**13ad**)

The title compound was prepared from gramine **8d** (56.0 mg, 0.2 mmol, 1 equiv) according to the general procedure. Column chromatography performed using gradient elution: 10:1 to 5:1 hexane/ethyl acetate. White solid. Yield: 67.3 mg, 0.172 mmol, 86%. ^1^H NMR (300 MHz, CDCl_3_) δ 8.07 (s, 1H), 7.62–7.51 (m, 2H), 7.48–7.28 (m, 5H), 7.18 (dd, *J* = 8.4, 3.9 Hz, 3H), 6.94 (d, *J* = 2.5 Hz, 1H), 6.86 (d, *J* = 2.2 Hz, 1H), 6.78 (dd, *J* = 8.7, 2.2 Hz, 1H), 5.07 (s, 2H), 4.47 (s, 2H), 2.38 (s, 3H). All spectroscopy data were coincident with those previously reported in the literature.^10e^

#### 4-Chloro-3-(tosylmethyl)-1*H*-indole (**13ae**)

The title compound was prepared from gramine **8e** (41.6 mg, 0.2 mmol, 1 equiv) according to the general procedure. Column chromatography performed using gradient elution: 10:1 to 5:1 hexane/ethyl acetate. White solid. Yield: 51.8 mg, 0.162 mmol, 81%. ^1^H NMR (300 MHz, CDCl_3_) δ 8.48 (s, 1H), 7.66–7.57 (m, 2H), 7.38 (d, *J* = 2.7 Hz, 1H), 7.27–7.16 (m, 3H), 7.11–6.6.96 (m, 2H), 4.93 (s, 2H), 2.40 (s, 3H). All spectroscopy data were coincident with those previously reported in the literature.^10e^

#### 5-Chloro-3-(tosylmethyl)-1*H*-indole (**13af**)

The title compound was prepared from gramine **8f** (41.6 mg, 0.2 mmol, 1 equiv) according to the general procedure. Column chromatography performed using gradient elution: 10:1 to 5:1 hexane/ethyl acetate. Pale orange foam. Yield: 57.0 mg, 0.178 mmol, 89%. ^1^H NMR (300 MHz, DMSO-*d*_6_) δ 11.33 (s, 1H), 7.59 (d, *J* = 8.1 Hz, 2H), 7.34 (dd, *J* = 8.5, 3.4 Hz, 4H), 7.22 (d, *J* = 2.6 Hz, 1H), 7.04 (dd, *J* = 8.6, 2.2 Hz, 1H), 4.74 (s, 2H), 2.37 (s, 3H). ^13^C {^1^H} NMR (75 MHz, DMSO-*d*_6_) δ 144.1, 135.8, 134.3, 129.5, 128.8, 128.2, 128.1, 123.7, 121.2, 118.3, 113.0, 101.7, 53.0, 21.0. HRMS (ESI) *m*/*z*: [M + Na]^+^ calcd for C_16_H_14_ClNO_2_SNa^+^, 342.0326; found, 342.0329. IR (cm^–1^): 3387, 1455, 1262, 1161.

#### 6-Chloro-3-(tosylmethyl)-1*H*-indole (**13ag**)

The title compound was prepared from gramine **8g** (41.6 mg, 0.2 mmol, 1 equiv) according to the general procedure. Column chromatography performed using gradient elution: 10:1 to 5:1 hexane/ethyl acetate. White solid. Yield: 58.2 mg, 0.182 mmol, 91%. ^1^H NMR (300 MHz, CDCl_3_) δ 8.20 (s, 1H), 7.63–7.42 (m, 2H), 7.39–7.31 (m, 1H), 7.23–7.12 (m, 3H), 7.12–7.05 (m, 1H), 7.00 (dt, *J* = 8.6, 1.6 Hz, 1H), 4.48 (t, *J* = 1.0 Hz, 2H), 2.39 (s, 3H). All spectroscopy data were coincident with those previously reported in the literature.^10^

#### 7-Chloro-3-(tosylmethyl)-1*H*-indole (**13ah**)

The title compound was prepared from gramine **8h** (41.6 mg, 0.2 mmol, 1 equiv) according to the general procedure. Column chromatography performed using gradient elution: 10:1 to 5:1 hexane/ethyl acetate. White solid, mp 203–205 °C. Yield: 57.6 mg, 0.180 mmol, 90%. ^1^H NMR (300 MHz, CDCl_3_) δ 8.42 (s, 1H), 7.55 (d, *J* = 8.1 Hz, 2H), 7.27–7.13 (m, 4H), 7.11 (d, *J* = 2.6 Hz, 1H), 6.98 (dd, *J* = 10.3, 5.4 Hz, 1H), 4.49 (s, 2H), 2.38 (s, 3H). ^13^C {^1^H} NMR (75 MHz, CDCl_3_) δ 144.7, 135.3, 129.7, 129.0, 128.7, 126.4, 122.1, 121.2, 117.6, 116.8, 110.1, 104.6, 54.5, 21.7. HRMS (ESI) *m*/*z*: [M + Na]^+^ calcd for C_16_H_14_ClNO_2_SNa^+^, 342.0326; found, 342.0328. IR (cm^–1^): 3389, 1459, 1257, 1163.

#### 6-Fluoro-3-(tosylmethyl)-1*H*-indole (**13ai**)

The title compound was prepared from gramine **8i** (38.4 mg, 0.2 mmol, 1 equiv) according to the general procedure. Column chromatography performed using gradient elution: 10:1 to 5:1 hexane/ethyl acetate. White solid. Yield: 51.6 mg, 0.170 mmol, 85%. ^1^H NMR (300 MHz, DMSO-*d*_6_) δ 11.15 (s, 1H), 7.64–7.49 (m, 2H), 7.39 (dd, *J* = 8.7, 5.5 Hz, 1H), 7.35–7.26 (m, 2H), 7.08 (dd, *J* = 8.2, 2.3 Hz, 2H), 6.77 (ddd, *J* = 9.7, 8.7, 2.4 Hz, 1H), 4.69 (s, 2H), 2.33 (s, 3H). ^19^F NMR (471 MHz, DMSO-*d*_6_) δ −121.73 (td, *J*_FF/FH_ = 7.8, 5.4 Hz). All spectroscopy data were coincident with those previously reported in the literature.^10e^

#### 5,6-Difluoro-3-(tosylmethyl)-1*H*-indole (**13aj**)

The title compound was prepared from gramine **8j** (42.0 mg, 0.2 mmol, 1 equiv) according to the general procedure. Column chromatography performed using gradient elution: 10:1 to 5:1 hexane/ethyl acetate. White foam. Yield: 59.1 mg, 0.184 mmol, 92%. ^1^H NMR (300 MHz, DMSO-*d*_6_) δ 11.29 (s, 1H), 7.59 (d, *J* = 8.2 Hz, 2H), 7.44–7.24 (m, 4H), 7.19 (d, *J* = 2.6 Hz, 1H), 4.73 (s, 2H), 2.36 (d, *J* = 1.9 Hz, 3H). ^13^C {^1^H} NMR (75 MHz, DMSO-*d*_6_) δ 146.1 (dd, *J* = 232.0, 15.7 Hz), 145.3 (dd, *J*_FC_ = 235.0, 15.0 Hz), 144.1, 135.8, 130.8 (d, *J* = 10.5 Hz), 129.5, 128.9 (d, *J* = 3.7 Hz), 128.1, 122.6 (d, *J* = 8.0 Hz), 105.7 (d, *J* = 19.5 Hz), 102.3 (d, *J* = 4.5 Hz), 99.4 (d, *J* = 21.0 Hz), 52.9, 21.0. ^19^F NMR (471 MHz, DMSO-*d*_6_) δ −145.22 (dd, *J*_FF/FH_ = 11.5, 2.1 Hz), −148.78 (dd, *J*_FF/FH_ = 11.5, 2.2 Hz). HRMS (ESI) *m*/*z*: [M + H]^+^ calcd for C_16_H_14_F_2_NO_2_S^+^, 322.0635; found, 322.0637. IR (cm^–1^): 3380, 1461, 1259, 1158.

#### 3-(Tosylmethyl)-5-(trifluoromethyl)-1*H*-indole (**13ak**)

The title compound was prepared from gramine **8k** (48.4 mg, 0.2 mmol, 1 equiv) according to the general procedure. Column chromatography performed using gradient elution: 10:1 to 5:1 hexane/ethyl acetate. Pale yellow foam. Yield: 55.8 mg, 0.158 mmol, 79%. ^1^H NMR (300 MHz, acetone-*d*_6_) δ 10.76 (s, 1H), 7.68–7.62 (m, 1H), 7.57 (d, *J* = 8.4 Hz, 3H), 7.45–7.39 (m, 1H), 7.35 (dd, *J* = 8.5, 1.7 Hz, 1H), 7.31–7.22 (m, 2H), 4.73 (s, 2H), 2.36 (s, 3H). ^13^C {^1^H} NMR (75 MHz, acetone-*d*_6_) δ 145.3, 138.6, 137.1, 130.2, 129.9, 129.8, 129.4, 126.5 (q, *J* = 269.0 Hz), 127.6, 122.0 (q, *J* = 21.0 Hz), 118.9 (q, *J* = 4.0 Hz), 117.6 (q, *J* = 4.0 Hz), 113.0, 110.9, 105.1, 54.2, 21.4. ^19^F NMR (471 MHz, acetone-*d*_6_) δ −58.88. HRMS (ESI) *m*/*z*: [M + H]^+^ calcd for C_17_H_15_F_3_NO_2_S^+^, 354.3592; found, 354.3594. IR (cm^–1^): 3394, 1459, 1264, 1155.

#### Methyl 3-(Tosylmethyl)-1*H*-indole-5-carboxylate (**13al**)

The title compound was prepared from gramine **8l** (46.4 mg, 0.2 mmol, 1 equiv) according to the general procedure. Column chromatography performed using gradient elution: 10:1 to 5:1 hexane/ethyl acetate. White solid, mp 228–230 °C. Yield: 60.2 mg, 0.176 mmol, 88%. ^1^H NMR (300 MHz, CDCl_3_) δ 8.48 (s, 1H), 7.90–7.79 (m, 2H), 7.62–7.51 (m, 2H), 7.34 (d, *J* = 9.1 Hz, 1H), 7.24 (d, *J* = 2.5 Hz, 1H), 7.17 (d, *J* = 8.0 Hz, 2H), 4.53 (s, 2H), 3.91 (s, 3H), 2.35 (s, 3H). ^13^C {^1^H} NMR (75 MHz, CDCl_3_) δ 144.9, 138.3, 135.3, 129.7, 128.7, 127.3, 126.7, 124.0, 122.5, 121.6, 111.2, 110.1, 104.8, 54.3, 52.0, 21.7. HRMS (ESI) *m*/*z*: [M + H]^+^ calcd for C_18_H_18_NO_4_S^+^, 344.3970; found, 344.3973. IR (cm^–1^): 3382, 1648, 1453, 1258.

#### 3-(Tosylmethyl)-1*H*-indole-5-carbonitrile (**13am**)

The title compound was prepared from gramine **8m** (39.8 mg, 0.2 mmol, 1 equiv) according to the general procedure. Column chromatography performed using gradient elution: 10:1 to 5:1 hexane/ethyl acetate. White solid. Yield: 52.8 mg, 0.170 mmol, 85%. ^1^H NMR (300 MHz, CDCl_3_) δ 8.53 (s, 1H), 7.56 (d, *J* = 7.8 Hz, 2H), 7.44–7.30 (m, 5H), 7.21 (s, 1H), 4.48 (s, 2H), 2.43 (s, 3H). All spectroscopy data were coincident with those previously reported in the literature.^10^

#### 3-(Tosylmethyl)-1*H*-indole-6-carbonitrile (**13an**)

The title compound was prepared from gramine **8n** (39.8 mg, 0.2 mmol, 1 equiv) according to the general procedure. Column chromatography performed using gradient elution: 10:1 to 5:1 hexane/ethyl acetate. White foam. Yield: 55.9 mg, 0.180 mmol, 90%. ^1^H NMR (300 MHz, DMSO-*d*_6_) δ 11.71 (s, 1H), 7.86 (s, 1H), 7.59 (dd, *J* = 8.2, 6.8 Hz, 3H), 7.41 (d, *J* = 2.6 Hz, 1H), 7.31 (dd, *J* = 16.6, 8.1 Hz, 3H), 4.80 (s, 2H), 2.36 (s, 3H). ^13^C {^1^H} NMR (75 MHz, DMSO-*d*_6_) δ 144.2, 135.7, 134.7, 131.5, 130.3, 129.6, 128.1, 121.6, 120.5, 120.3, 116.6, 102.9, 102.8, 52.6, 21.1. HRMS (ESI) *m*/*z*: [M + H]^+^ calcd for C_17_H_15_N_2_O_2_S^+^, 311.0849; found, 311.0856. IR (cm^–1^): 3392, 1447, 1255, 1159.

#### 5-(4,4,5,5-Tetramethyl-1,3,2-dioxaborolan-2-yl)-3-(tosylmethyl)-1*H*-indole (**13ap**)

The title compound was prepared from gramine **8p** (60.2 mg, 0.2 mmol, 1 equiv) according to the general procedure. Column chromatography performed using gradient elution: 10:1 to 5:1 hexane/ethyl acetate. White foam. Yield: 76.6 mg, 0.186 mmol, 93%. ^1^H NMR (300 MHz, CDCl_3_) δ 8.60 (s, 1H), 7.72 (d, *J* = 1.0 Hz, 1H), 7.63–7.49 (m, 3H), 7.28 (dd, *J* = 8.2, 0.8 Hz, 1H), 7.19–6.99 (m, 3H), 4.53 (s, 2H), 2.33 (s, 3H), 1.35 (s, 12H). ^13^C {^1^H} NMR (75 MHz, CDCl_3_) δ 144.5, 137.8, 135.4, 129.7, 128.7, 128.5, 126.8, 126.1, 126.0, 110.9, 103.3, 83.6, 54.2, 25.0, 21.7. HRMS (ESI) *m*/*z*: [M + H]^+^ calcd for C_22_H_27_BNO_4_S^+^, 412.3230; found, 412.3227. IR (cm^–1^): 3345, 1460, 1265, 1163.

#### 2-Methyl-3-(tosylmethyl)-1*H*-indole (**13ar**)

The title compound was prepared from gramine **8r** (37.6 mg, 0.2 mmol, 1 equiv) according to the general procedure. Column chromatography performed using gradient elution: 10:1 to 5:1 hexane/ethyl acetate. White solid. Yield: 49.0 mg, 0.164 mmol, 82%. ^1^H NMR (300 MHz, acetone-*d*_6_) δ 10.11 (s, 1H), 7.61–7.46 (m, 2H), 7.38–7.16 (m, 4H), 6.99 (ddd, *J* = 8.1, 7.0, 1.3 Hz, 1H), 6.90 (ddd, *J* = 8.0, 7.0, 1.1 Hz, 1H), 4.52 (s, 2H), 2.39 (s, 3H), 2.08 (s, 3H). All spectroscopy data were coincident with those previously reported in the literature.^10e^

#### 2-Phenyl-3-(tosylmethyl)-1*H*-indole (**13as**)

The title compound was prepared from gramine **8s** (50.0 mg, 0.2 mmol, 1 equiv) according to the general procedure. Column chromatography performed using gradient elution: 10:1 to 5:1 hexane/ethyl acetate. White solid. Yield: 60.6 mg, 0.168 mmol, 84%. ^1^H NMR (300 MHz, CDCl_3_) δ 8.27 (s, 1H), 7.50 (dd, *J* = 8.2, 6.5 Hz, 3H), 7.37 (s, 6H), 7.21 (ddd, *J* = 8.2, 7.0, 1.2 Hz, 1H), 7.15–7.06 (m, 3H), 4.64 (s, 2H), 2.38 (s, 3H). All spectroscopy data were coincident with those previously reported in the literature.^10e^

#### 3-(Tosylmethyl)-1*H*-pyrrolo­[2,3-*b*]­pyridine (**16aq**)

The title compound was prepared from gramine **15** (35.0 mg, 0.2 mmol, 1 equiv) according to the general procedure. Column chromatography performed using gradient elution: 10:1 to 5:1 hexane/ethyl acetate. Light orange foam. Yield: 48.0 mg, 0.168 mmol, 84%. ^1^H NMR (300 MHz, CDCl_3_) δ 10.05 (s, 1H), 8.44–8.19 (m, 1H), 7.74 (dd, *J* = 8.0, 1.5 Hz, 1H), 7.54 (dd, *J* = 8.5, 1.9 Hz, 2H), 7.23–7.12 (m, 3H), 7.04 (dd, *J* = 7.9, 4.8 Hz, 1H), 4.49 (s, 2H), 2.39 (s, 3H). ^13^C {^1^H} NMR (75 MHz, CDCl_3_) 148.4, 144.8, 143.7, 135.2, 129.7, 128.7, 127.9, 126.4, 116.6, 110.1, 102.1, 54.7, 21.4. HRMS (ESI) *m*/*z*: [M + H]^+^ calcd for C_15_H_15_N_2_O_2_S^+^, 287.0849; found, 287.0855. IR (cm^–1^): 3393, 1458, 1260, 1161.

#### 1-Methyl-3-(tosylmethyl)-1*H*-indole (**13at**)

The title compound was prepared from gramine **8t** (37.6 mg, 0.2 mmol, 1 equiv) according to the general procedure. Column chromatography performed using gradient elution: 10:1 to 5:1 hexane/ethyl acetate. White solid. Yield: 42.4 mg, 0.142 mmol, 71%. ^1^H NMR (300 MHz, CDCl_3_) δ 7.58 (dd, *J* = 8.8, 2.1 Hz, 2H), 7.32–7.27 (m, 1H), 7.19 (s, 3H), 7.10–6.97 (m, 3H), 4.50 (d, *J* = 2.5 Hz, 2H), 3.77 (d, *J* = 2.8 Hz, 3H), 2.39 (d, *J* = 2.4 Hz, 3H). All spectroscopy data were coincident with those previously reported in the literature.^10e^

### General Procedure for the Coupling between Gramines 8 and α-Amido Sulfones 1—Method B: Synthesis of Product 14 (Scheme 2)

To a solution of the corresponding gramine **8** (0.24 mmol, 1.2 equiv) in acetone (2 mL), the respective α-amido sulfone **1** was added as a solid (0.2 mmol, 1.0 equiv). Then, the reaction was cooled to 0 °C (bath temperature), and ethyl propiolate **12** (31.4 mg, 0.32 mmol, 1.6 equiv) was added dropwise. After stirring the reaction mixture at 0 °C for 30 min, the solvent was evaporated and the crude product was purified by flash column chromatography using as eluent a gradient mixture of hexane/ethyl acetate leading in most cases to essentially pure product **14**. If needed, further purification was applied by washing the resulting material with *n*-hexane and drying under vacuum.

#### *tert*-Butyl (Phenyl­(3-(tosylmethyl)-1*H*-indol-1-yl)­methyl)­carbamate (**14aa**)

The title compound was prepared from gramine **8a** (41.8 mg, 0.24 mmol, 1.2 equiv) and α-amido sulfone **1a** (72 mg, 0.2 mmol, 1 equiv) according to the general procedure. Column chromatography performed using gradient elution: 10:1 to 5:1 hexane/ethyl acetate. White solid, mp 162–165 °C. Yield: 84.4, 0.172 mmol, 86%. ^1^H NMR (300 MHz, CDCl_3_) δ 7.58–7.45 (m, 2H), 7.42–7.31 (m, 4H), 7.26 (s, 1H), 7.19–7.10 (m, 5H), 7.05 (td, *J* = 7.4, 1.2 Hz, 1H), 6.96 (s, 1H), 5.45 (s, 1H), 4.49 (s, 2H), 2.36 (s, 3H), 1.44 (s, 9H). ^13^C {^1^H} NMR (75 MHz, CDCl_3_) δ 155.0, 145.0, 138.1, 136.2, 135.8, 130.0, 129.6, 129.5, 129.3, 128.8, 127.4, 126.9, 123.1, 121.2, 119.7, 111.2, 103.6, 81.6, 65.7, 55.0, 28.9, 22.2. HRMS (ESI) *m*/*z*: [M + H]^+^ calcd for C_28_H_31_N_2_O_4_S^+^, 491.1999; found, 491.2006. IR (cm^–1^): 3317, 1671, 1525, 1458, 1300, 1252.

(**Reaction run at 6 mmol scale**) To a solution of gramine **8a** (1.25 g, 7.2 mmol, 1.2 equiv) in acetone (5 mL), α-amido sulfone **1a** (2.17 g, 6 mmol, 1 equiv) was added. Then, the reaction mixture was cooled to 0 °C (bath temperature), and ethyl propiolate **12** (942 mg, 9.6 mmol, 1.6 equiv) was added dropwise. After stirring the reaction mixture at 0 °C for 60 min, the solvent was evaporated and the crude product was purified by flash column chromatography (gradient elution: 10:1 to 5:1 hexane/ethyl acetate). The resulting material was washed with *n*-hexane and dried under vacuum to yield essentially pure **14aa** as a white solid: 2.35 g (4.8 mmol, 80%).

#### *tert*-Butyl (1-(3-(Tosylmethyl)-1*H*-indol-1-yl)­propyl)­carbamate (**14ba**)

The title compound was prepared from gramine **8a** (41.8 mg, 0.24 mmol, 1.2 equiv) and α-amido sulfone **1b** (62.6 mg, 0.2 mmol, 1 equiv) according to the general procedure. Column chromatography performed using gradient elution: 10:1 to 5:1 hexane/ethyl acetate. White solid, mp 160–164 °C. Yield: 63.6 mg, 0.144 mmol, 72%. ^1^H NMR (300 MHz, CDCl_3_) δ 7.57–7.46 (m, 2H), 7.26 (s, 2H), 7.22–7.08 (m, 2H), 7.02 (ddd, *J* = 9.0, 6.3, 0.9 Hz, 2H), 5.89 (d, *J* = 13.9 Hz, 1H), 5.07 (s, 1H), 4.49 (s, 2H), 2.36 (s, 3H), 2.03 (dd, *J* = 11.5, 4.5 Hz, 2H), 1.39 (s, 9H), 0.94–0.74 (m, 3H). ^13^C {^1^H} NMR (75 MHz, CDCl_3_) δ 154.6, 144.5, 135.8, 135.3, 129.5, 128.8, 127.9, 125.5, 122.5, 120.5, 118.9, 110.5, 110.1, 103.0, 77.4, 64.3, 54.6, 28.7, 28.4, 21.7, 10.1. HRMS (ESI) *m*/*z*: [M + H]^+^ calcd for C_24_H_31_N_2_O_4_S^+^, 443.5740; found, 443.5745. IR (cm^–1^): 3327, 1673, 1526, 1453, 1298.

#### *tert*-Butyl (Cyclohexyl­(3-((phenylsulfonyl)­methyl)-1*H*-indol-1-yl)­methyl)­carbamate (**14ca**)

The title compound was prepared from gramine **8a** (41.82 mg, 0.24 mmol, 1.2 equiv) and α-amido sulfone **1c** (72.4 mg, 0.2 mmol, 1 equiv) according to the general procedure. Column chromatography performed using gradient elution: 10:1 to 5:1 hexane/ethyl acetate. White solid, mp 163–166 °C. Yield: 69.5 mg, 0.144 mmol, 72%. ^1^H NMR (300 MHz, CDCl_3_) δ 7.67–7.56 (d, *J* = 7.6 Hz, 2H), 7.48 (m, 2H), 7.30–7.38 (m, 3H), 7.15 (t, *J* = 7.8 Hz, 1H), 7.02 (t, *J* = 7.5 Hz, 1H), 6.94 (s, 1H), 5.61 (s_b_, 1H), 5.18 (d, *J* = 8.9 Hz, 1H), 4.53 (s, 2H), 1.98–1.75 (m, 2H), 1.70–1.50 (m, 2H), 1.37 (s, 9H), 1.25–0.75 (m, 7H). ^13^C {^1^H} NMR (75 MHz, CDCl_3_) δ 154.9, 138.0, 135.9, 133.5, 129.2, 128.8, 127.6, 126.0, 122.5, 120.4, 118.9, 110.4, 102.7, 80.5, 67.6, 54.5, 42.5, 29.7, 29.1, 28.4, 26.0, 25.7. HRMS (ESI) *m*/*z*: [M + H]^+^ calcd for C_27_H_35_N_2_O_4_S^+^, 482.2239; found, 482.2242. IR (cm^–1^): 3347, 1670, 1524, 1458, 1301.

#### *tert*-Butyl ((3-(Tosylmethyl)-1*H*-indol-1-yl)­methyl)­carbamate (**14da**)

The title compound was prepared from gramine **8a** (41.8 mg, 0.24 mmol, 1.2 equiv) and α-amido sulfone **1d** (57 mg, 0.2 mmol, 1 equiv) according to the general procedure. Column chromatography performed using gradient elution: 10:1 to 5:1 hexane/ethyl acetate. White solid, mp 159–162 °C. Yield: 50.6 mg, 0.122 mmol, 61%. ^1^H NMR (300 MHz, CDCl_3_) δ 7.57 (d, *J* = 8.1 Hz, 2H), 7.53–7.37 (m, 1H), 7.32 (d, *J* = 8.0 Hz, 1H), 7.20 (t, *J* = 8.1 Hz, 3H), 7.12 (d, *J* = 3.4 Hz, 1H), 7.10–6.95 (m, 1H), 5.41 (d, *J* = 6.5 Hz, 1H), 5.30 (d, *J* = 8.6 Hz, 1H), 4.47 (d, *J* = 2.7 Hz, 2H), 2.38 (d, *J* = 3.2 Hz, 3H), 1.43 (s, 9H). ^13^C {^1^H} NMR (75 MHz, CDCl_3_) δ 144.6, 141.6, 135.7, 129.6, 129.4, 128.7, 122.7, 120.6, 119.2, 109.8, 102.8, 99.7, 80.7, 54.5, 51.9, 28.4, 21.7. HRMS (ESI) *m*/*z*: [M + H]^+^ calcd for C_22_H_27_N_2_O_4_S^+^, 415.5200; found, 415.5202. IR (cm^–1^): 3378, 1692, 1134, 736.

#### *tert*-Butyl ((4-Chlorophenyl)­(3-(tosylmethyl)-1*H*-indol-1-yl)­methyl) carbamate (**14ea**)

The title compound was prepared from gramine **8a** (41.8 mg, 0.24 mmol, 1.2 equiv) and α-amido sulfone **1e** (79 mg, 0.2 mmol, 1 equiv) according to the general procedure. Column chromatography performed using gradient elution: 10:1 to 5:1 hexane/ethyl acetate. White solid, mp 165–168 °C. Yield: 87.2 mg, 0.166 mmol, 83%. ^1^H NMR (300 MHz, CDCl_3_) δ 7.61–7.49 (m, 2H), 7.39 (d, *J* = 7.7 Hz, 1H), 7.31 (dt, *J* = 8.4, 2.3 Hz, 2H), 7.25 (dd, *J* = 9.4, 6.3 Hz, 1H), 7.20–7.01 (m, 5H), 6.94 (d, *J* = 3.5 Hz, 2H), 5.45 (d, *J* = 8.8 Hz, 1H), 4.49 (d, *J* = 3.7 Hz, 2H), 2.38 (d, *J* = 3.8 Hz, 3H), 1.44 (s, 9H). ^13^C {^1^H} NMR (75 MHz, CDCl_3_) δ 144.6, 136.2, 135.5, 135.4, 135.0, 129.5, 129.3, 128.7, 128.3, 127.8, 126.7, 122.9, 120.9, 119.4, 110.6, 110.1, 103.5, 81.4, 64.7, 54.4, 28.4, 21.7. HRMS (ESI) *m*/*z*: [M + H]^+^ calcd for C_28_H_30_ClN_2_O_4_S^+^, 525.0600; found, 525.0606. IR (cm^–1^): 3342, 1685, 1522, 1458, 1303.

#### *tert*-Butyl ((4-Methoxyphenyl)­(3-(tosylmethyl)-1*H*-indol-1-yl)­methyl) carbamate (**14fa**)

The title compound was prepared from gramine **8a** (41.8 mg, 0.24 mmol, 1.2 equiv) and α-amido sulfone **1f** (78.2 mg, 0.2 mmol, 1 equiv) according to the general procedure. Column chromatography performed using gradient elution: 10:1 to 5:1 hexane/ethyl acetate. White solid, mp 164–166 °C. Yield: 87.4 mg, 0.168 mmol, 84%. ^1^H NMR (300 MHz, CDCl_3_) δ 7.57–7.50 (m, 1H), 7.45–7.32 (m, 2H), 7.26 (d, *J* = 8.1 Hz, 1H), 7.19–7.08 (m, 4H), 7.08–7.01 (m, 2H), 6.97 (s, 1H), 6.89–6.79 (m, 2H), 5.39 (d, *J* = 9.6 Hz, 1H), 4.49 (s, 2H), 3.80 (d, *J* = 4.7 Hz, 3H), 2.37 (s, 3H), 1.44 (s, 9H). ^13^C {^1^H} NMR (75 MHz, CDCl_3_) δ 160.0, 154.5, 153.0, 144.5, 135.6, 135.4, 129.5, 128.8, 127.6, 126.9, 122.6, 120.7, 119.2, 114.4, 110.7, 109.8, 103.0, 81.1, 64.9, 55.5, 54.6, 28.4, 21.7. HRMS (ESI) *m*/*z*: [M + H]^+^ calcd for C_29_H_33_N_2_O_5_S^+^, 521.2032; found, 521.2033. IR (cm^–1^): 3347, 1677, 1522, 1453, 1306.

#### *tert*-Butyl ((5-Methyl-3-(tosylmethyl)-1*H*-indol-1-yl)­(phenyl)­methyl)­carbamate (**14ab**)

The title compound was prepared from gramine **8b** (45.2 mg, 0.24 mmol, 1.2 equiv) and α-amido sulfone **1a** (72 mg, 0.2 mmol, 1 equiv) according to the general procedure. Column chromatography performed using gradient elution: 10:1 to 5:1 hexane/ethyl acetate. White solid, mp 174–176 °C. Yield: 84.8 mg, 0.168 mmol, 84%. ^1^H NMR (300 MHz, CDCl_3_) δ 7.53 (d, *J* = 8.1 Hz, 2H), 7.3–7.30 (m, 2H), 7.14 (dd, *J* = 7.7, 5.4 Hz, 5H), 7.05 (s, 2H), 6.96–6.89 (m, 2H), 5.51 (s, 1H), 4.46 (s, 2H), 2.37 (s, 3H), 2.34 (s, 3H), 1.44 (s, 9H). ^13^C {^1^H} NMR (75 MHz, CDCl_3_) δ 154.5, 144.4, 137.7, 135.3, 134.0, 130.0, 129.4, 129.3, 129.0, 128.9, 128.8, 128.5, 127.0, 126.3, 118.8, 110.3, 102.6, 81.0, 65.2, 54.6, 28.4, 21.7, 21.4. HRMS (ESI) *m*/*z*: [M + H]^+^ calcd for C_29_H_33_N_2_O_4_S^+^, 505.2083; found, 505.2087. IR (cm^–1^): 3365, 1523, 1665, 1294.

#### *tert*-Butyl ((5-Methoxy-3-(tosylmethyl)-1*H*-indol-1-yl)­(phenyl)­methyl)­carbamate (**14ac**)

The title compound was prepared from gramine **8c** (49.0 mg, 0.24 mmol, 1.2 equiv) and α-amido sulfone **1a** (72 mg, 0.2 mmol, 1 equiv) according to the general procedure. Column chromatography performed using gradient elution: 10:1 to 5:1 hexane/ethyl acetate. White solid, mp 176–180 °C. Yield: 85.4 mg, 0.164 mmol, 82%. ^1^H NMR (300 MHz, CDCl_3_) δ 7.53 (dt, *J* = 8.3, 1.9 Hz, 2H), 7.35 (dp, *J* = 4.9, 1.7 Hz, 3H), 7.21–7.03 (m, 5H), 7.00–6.87 (m, 1H), 6.86–6.65 (m, 2H), 5.45 (s, 1H), 4.56–4.35 (m, 2H), 3.77–3.70 (m, 3H), 2.37 (d, *J* = 2.8 Hz, 3H), 1.44 (s, 9H). ^13^C {^1^H} NMR (75 MHz, CDCl_3_) δ 154.9, 144.5, 137.6, 135.3, 130.7, 129.5, 129.1, 129.0, 128.9, 128.8, 127.4, 113.1, 111.5, 110.7, 110.1, 102.8, 100.6, 81.1, 65.4, 55.7, 54.7, 28.4, 21.7. HRMS (ESI) *m*/*z*: [M + H]^+^ calcd for C_29_H_33_N_2_O_5_S^+^, 521.2032; found, 521.2038. IR (cm^–1^): 3323, 1512, 1652, 1288.

#### *tert*-Butyl ((6-(Benzyloxy)-3-(tosylmethyl)-1*H*-indol-1-yl)­(phenyl)­methyl)­carbamate (**14ad**)

The title compound was prepared from gramine **8d** (67.2 mg, 0.24 mmol, 1.2 equiv) and α-amido sulfone **1a** (72 mg, 0.2 mmol, 1 equiv) according to the general procedure. Column chromatography performed using gradient elution: 10:1 to 5:1 hexane/ethyl acetate. White solid, mp 213–215 °C. Yield: 105 mg, 0.176 mmol, 88%. ^1^H NMR (300 MHz, CDCl_3_) δ 7.59:7.50 (m, 2H), 7.41:7.32 (m, 6H), 7.26:4H, 7.19:7.07 (m, 3H), 6.87:6.75 (m, 3H), 5.38:1H, 4.99:2H, 4.44:2H, 2.37:3H, 1.45 (s, 9H). ^13^C {^1^H} NMR (75 MHz, CDCl_3_) δ 156.0, 144.5, 137.4, 136.4, 135.4, 129.5, 129.1, 129.0, 128.8, 128.7, 128.0, 127.8, 126.4, 125.8, 122.8, 120.1, 111.4, 110.1, 103.2, 95.7, 81.2, 70.7, 65.1, 54.7, 28.4, 21.7. HRMS (ESI) *m*/*z*: [M + H]^+^ calcd for C_35_H_37_N_2_O_5_S^+^, 597.2345; found, 597.2349. IR (cm^–1^): 3333, 1534, 1620, 1293.

#### *tert*-Butyl ((4-Chloro-3-(tosylmethyl)-1*H*-indol-1-yl)­(phenyl)­methyl)­carbamate (**14ae**)

The title compound was prepared from gramine **8e** (50.1 mg, 0.24 mmol, 1.2 equiv) and α-amido sulfone **1a** (72 mg, 0.2 mmol, 1 equiv) according to the general procedure. Column chromatography performed using gradient elution: 10:1 to 5:1 hexane/ethyl acetate. White solid, mp 158–160 °C. Yield: 76.6 mg, 0.146 mmol, 73%. ^1^H NMR (300 MHz, CDCl_3_) δ 7.62–7.49 (m, 2H), 7.42–7.35 (m, 2H), 7.32 (s, 1H), 7.16 (td, *J* = 8.2, 5.2 Hz, 5H), 7.09–6.90 (m, 3H), 5.54 (s, 1H), 5.04–4.71 (m, 2H), 2.34 (s, 3H), 1.46 (s, 9H). ^13^C {^1^H} NMR (75 MHz, CDCl_3_) δ 164.8, 152.7, 144.4, 136.1, 129.4, 129.3, 128.9, 128.2, 126.3, 123.0, 121.9, 109.6, 94.2, 54.0, 28.4, 21.7. HRMS (ESI) *m*/*z*: [M + H]^+^ calcd for C_28_H_30_ClN_2_O_4_S^+^, 525.1537; found, 525.1538. IR (cm^–1^): 3321, 1672, 1528, 1455, 1298.

#### *tert*-Butyl ((5-Chloro-3-(tosylmethyl)-1*H*-indol-1-yl)­(phenyl)­methyl)­carbamate (**14af**)

The title compound was prepared from gramine **8f** (50.1 mg, 0.24 mmol, 1.2 equiv) and α-amido sulfone **1a** (72 mg, 0.2 mmol, 1 equiv) according to the general procedure. Column chromatography performed using gradient elution: 10:1 to 5:1 hexane/ethyl acetate. White solid, mp 160–162 °C. Yield: 78.8 mg, 0.15 mmol, 75%. ^1^H NMR (300 MHz, CDCl_3_) δ 7.52 (dd, *J* = 8.4, 2.4 Hz, 2H), 7.41–7.33 (m, 3H), 7.14 (ddd, *J* = 13.0, 7.3, 2.6 Hz, 6H), 7.08–6.96 (m, 2H), 5.50 (s, 1H), 4.42 (d, *J* = 2.4 Hz, 2H), 2.39 (d, *J* = 2.5 Hz, 3H), 1.45 (s, 9H). ^13^C {^1^H} NMR (75 MHz, CDCl_3_) δ 144.9, 137.2, 135.1, 134.0, 129.6, 129.3, 129.2, 128.9, 128.2, 126.6, 126.3, 123.0, 118.5, 111.7, 110.1, 103.0, 81.4, 65.4, 54.5, 28.4, 21.7. HRMS (ESI) *m*/*z*: [M + H]^+^ calcd for C_28_H_30_ClN_2_O_4_S^+^, 525.1537; found, 525.1539. IR (cm^–1^): 3320, 1672, 1527, 1455, 1299.

#### *tert*-Butyl ((6-Chloro-3-(tosylmethyl)-1*H*-indol-1-yl)­(phenyl)­methyl)­carbamate (**14ag**)

The title compound was prepared from gramine **8g** (50.1 mg, 0.24 mmol, 1.2 equiv) and α-amido sulfone **1a** (72 mg, 0.2 mmol, 1 equiv) according to the general procedure. Column chromatography performed using gradient elution: 10:1 to 5:1 hexane/ethyl acetate. White solid, mp 170–172 °C. Yield: 94.4 mg, 0.18 mmol, 90%. ^1^H NMR (300 MHz, CDCl_3_) δ 7.52 (dt, *J* = 8.3, 1.7 Hz, 2H), 7.40–7.31 (m, 5H), 7.17 (dd, *J* = 8.3, 2.7 Hz, 2H), 7.10 (dq, *J* = 9.5, 4.5 Hz, 2H), 7.05–6.91 (m, 2H), 5.48 (s, 1H), 4.46 (d, *J* = 2.7 Hz, 2H), 2.38 (d, *J* = 2.8 Hz, 3H), 1.45 (s, 9H). ^13^C {^1^H} NMR (75 MHz, CDCl_3_) δ 144.7, 137.1, 136.0, 135.2, 129.6, 129.3, 129.2, 128.9, 128.7, 127.5, 126.8, 126.3, 121.5, 120.2, 110.7, 110.1, 103.4, 77.4, 65.5, 54.4, 28.4, 21.7. HRMS (ESI) *m*/*z*: [M + H]^+^ calcd for C_28_H_30_ClN_2_O_4_S^+^, 525.1537; found, 525.15338. IR (cm^–1^): 3322, 16725, 1525, 1456, 1299.

#### *tert*-Butyl ((5,6-Difluoro-3-(tosylmethyl)-1*H*-indol-1-yl)­(phenyl)­methyl)­carbamate (**14aj**)

The title compound was prepared from gramine **8j** (50.4 mg, 0.24 mmol, 1.2 equiv) and α-amido sulfone **1a** (72 mg, 0.2 mmol, 1 equiv) according to the general procedure. Column chromatography performed using gradient elution: 10:1 to 5:1 hexane/ethyl acetate. Orange foam. Yield: 89.5 mg, 0.170 mmol, 85%. ^1^H NMR (300 MHz, CDCl_3_) δ 7.53 (dd, *J* = 8.4, 2.4 Hz, 2H), 7.46–7.34 (m, 3H), 7.19 (dd, *J* = 8.4, 2.3 Hz, 2H), 7.14 (s, 5H), 5.47 (s, 1H), 4.50–4.28 (m, 2H), 2.39 (d, *J* = 2.5 Hz, 3H), 1.47 (d, *J* = 9.2 Hz, 9H). ^13^C {^1^H} NMR (75 MHz, CDCl_3_) δ 148.2 (dd, *J* = 247.0, 16.0 Hz), 147.9 (dd, *J* = 243.7, 16.0 Hz), 147.8 (d, *J* = 22.5 Hz), 145.1 (d, *J* = 19.5 Hz), 144.9, 136.9, 135.2, 130.7 (d, *J* = 10.5 Hz), 130.2 (d, *J* = 3.7 Hz), 129.6, 129.4, 129.3, 128.8, 128.1 (d, *J* = 3.2 Hz), 126.3, 110.1, 106.1 (d, *J* = 19.5 Hz), 103.4, 99.0 (d, *J* = 22.5 Hz), 81.5, 65.7, 54.5, 28.4, 21.7. ^19^F NMR (471 MHz, CDCl_3_) δ −141.70 (dd, *J*_FF/FH_ = 10.8, 2.1 Hz), −145.76 (dd, *J*_FF/FH_ = 10.8, 2.2 Hz). HRMS (ESI) *m*/*z*: [M + H]^+^ calcd for C_28_H_29_F_2_N_2_O_4_S^+^, 527.1738; found, 527.1744. IR (cm^–1^): 3325, 1634, 1502, 1442, 1313.

#### Methyl 1-(((*tert*-butoxycarbonyl)­amino)­(phenyl)­methyl)-3-(tosylmethyl)-1*H*-indole-5-carboxylate (**14al**)

The title compound was prepared from gramine **8l** (55.8 mg, 0.24 mmol, 1.2 equiv) and α-amido sulfone **1a** (72 mg, 0.2 mmol, 1 equiv) according to the general procedure. Column chromatography performed using gradient elution: 10:1 to 5:1 hexane/ethyl acetate. White solid, mp 181–184 °C. Yield: 92.2 mg, 0.168 mmol, 84%. ^1^H NMR (300 MHz, CDCl_3_) δ 7.91 (s, 1H), 7.80 (dd, *J* = 8.7, 1.8 Hz, 2H), 7.59–7.48 (m, 3H), 7.37 (dq, *J* = 4.3, 2.4 Hz, 2H), 7.30 (s, 1H), 7.19 (d, *J* = 1.8 Hz, 1H), 7.14 (d, *J* = 7.3 Hz, 3H), 5.55 (s, 1H), 4.51 (s, 2H), 3.90 (d, *J* = 1.5 Hz, 3H), 2.33 (s, 3H), 1.53–1.33 (m, 9H). ^13^C {^1^H} NMR (75 MHz, CDCl_3_) δ 167.7, 144.8, 138.0, 137.1, 135.1, 129.6, 129.4, 129.3, 128.8, 128.1, 127.8, 126.2, 123.9, 122.8, 121.8, 110.4, 110.1, 104.7, 81.5, 65.5, 54.2, 52.0, 28.4, 21.6. HRMS (ESI) *m*/*z*: [M + H]^+^ calcd for C_30_H_33_N_2_O_6_S^+^, 549.1981; found, 549.1987. IR (cm^–1^): 3320, 1692, 1520, 1445, 1307.

#### *tert*-Butyl ((5-Cyano-3-(tosylmethyl)-1*H*-indol-1-yl)­(phenyl)­methyl)­carbamate (**14am**)

The title compound was prepared from gramine **8m** (47.8 mg, 0.24 mmol, 1.2 equiv) and α-amido sulfone **1a** (72 mg, 0.2 mmol, 1 equiv) according to the general procedure. Column chromatography performed using gradient elution: 10:1 to 5:1 hexane/ethyl acetate. White solid, mp 166–167 °C. Yield: 67.0 mg, 0.130 mmol, 65%. ^1^H NMR (300 MHz, CDCl_3_) δ 7.57–7.48 (m, 2H), 7.45 (s, 1H), 7.43–7.34 (m, 3H), 7.32 (d, *J* = 1.4 Hz, 1H), 7.29 (d, *J* = 2.8 Hz, 1H), 7.19 (s, 2H), 7.16 (s, 1H), 7.14–7.08 (m, 2H), 5.73 (d, *J* = 8.5 Hz, 1H), 4.45 (s, 2H), 2.39 (s, 3H), 1.54–1.32 (m, 9H). ^13^C {^1^H} NMR (75 MHz, CDCl_3_) δ 161.6, 154.4, 145.2, 137.1, 136.7, 135.0, 129.8, 129.4, 129.3, 129.2, 129.1, 128.7, 127.9, 126.2, 125.3, 124.6, 120.1, 111.6, 104.2, 81.6, 65.6, 54.2, 28.3, 21.7. HRMS (ESI) *m*/*z*: [M + H]^+^ calcd for C_29_H_30_N_3_O_4_S^+^, 516.1879; found, 516.1881. IR (cm^–1^): 3322, 1670, 1523, 1439, 1304.

#### *tert*-Butyl ((6-Cyano-3-(tosylmethyl)-1*H*-indol-1-yl)­(phenyl)­methyl)­carbamate (**14an**)

The title compound was prepared from gramine **8n** (47.8 mg, 0.24 mmol, 1.2 equiv) and α-amido sulfone **1a** (72 mg, 0.2 mmol, 1 equiv) according to the general procedure. Column chromatography performed using gradient elution: 10:1 to 5:1 hexane/ethyl acetate. White solid, mp 168–171 °C. Yield: 58.8 mg, 0.114 mmol, 57%. ^1^H NMR (300 MHz, acetone-*d*_6_) δ 8.00 (s, 1H), 7.79 (s, 1H), 7.70 (dd, *J* = 8.3, 0.8 Hz, 1H), 7.60–7.48 (m, 1H), 7.44 (d, *J* = 4.3 Hz, 1H), 7.40 (td, *J* = 5.3, 2.3 Hz, 4H), 7.37–7.32 (m, 2H), 7.30 (d, *J* = 1.2 Hz, 1H), 7.29–7.22 (m, 2H), 4.71 (d, *J* = 1.6 Hz, 2H), 2.38 (s, 3H), 1.41 (s, 9H). ^13^C {^1^H} NMR (75 MHz, acetone-*d*_6_) δ 145.2, 138.4, 136.7, 135.4, 132.4, 130.3, 129.7, 129.6, 129.4, 127.4, 123.3, 121.6, 120.7, 116.7, 110.9, 105.3, 105.2, 80.6, 66.5, 53.8, 28.4, 21.5. HRMS (ESI) *m*/*z*: [M + H]^+^ calcd for C_29_H_30_N_3_O_4_S^+^, 516.1879; found, 516.1883. IR (cm^–1^): 3324, 1672, 1521, 1441, 1304.

#### *tert*-Butyl ((6-Nitro-3-(tosylmethyl)-1*H*-indol-1-yl)­(phenyl)­methyl)­carbamate (**14ao**)

The title compound was prepared from gramine **8o** (52.6 mg, 0.24 mmol, 1.2 equiv) and α-amido sulfone **1a** (72 mg, 0.2 mmol, 1 equiv) according to the general procedure. Column chromatography performed using gradient elution: 10:1 to 5:1 hexane/ethyl acetate. White solid, mp 151–155 °C. Yield: 80.4 mg, 0.150 mmol, 75%. ^1^H NMR (300 MHz, CDCl_3_) δ 8.23 (d, *J* = 1.9 Hz, 1H), 7.93 (dd, *J* = 9.2, 1.9 Hz, 1H), 7.59–7.48 (m, 2H), 7.40 (tt, *J* = 5.2, 2.9 Hz, 4H), 7.18 (d, *J* = 8.0 Hz, 2H), 7.15–7.08 (m, 2H), 5.56 (s, 1H), 4.50 (s, 3H), 2.37 (s, 3H), 1.45 (s, 9H). ^13^C {^1^H} NMR (75 MHz, CDCl_3_) δ 145.0, 143.7, 136.5, 135.0, 132.2, 129.8, 129.7, 129.5, 128.7, 126.1, 119.3, 116.1, 110.1, 107.7, 98.7, 81.9, 66.3, 54.1, 28.3, 21.8. HRMS (ESI) *m*/*z*: [M + H]^+^ calcd for C_28_H_30_N_3_O_6_S^+^, 536.1777; found, 536.1779. IR (cm^–1^): 3315, 1666, 1518, 1452, 1298.

#### *tert*-Butyl ((7-Chloro-3-(tosylmethyl)-1*H*-indol-1-yl)­methyl)­carbamate (**14dh**)

The title compound was prepared from gramine **8h** (50.1 mg, 0.24 mmol, 1.2 equiv) and α-amido sulfone **1d** (57.0 mg, 0.2 mmol, 1 equiv) according to the general procedure. Column chromatography performed using gradient elution: 10:1 to 5:1 hexane/ethyl acetate. White solid, mp 168–171 °C. Yield: 71.8 mg, 0.160 mmol, 80%. ^1^H NMR (300 MHz, CDCl_3_) δ 7.61–7.53 (m, 2H), 7.36 (d, *J* = 8.0 Hz, 1H), 7.24–7.12 (m, 4H), 7.09–6.89 (m, 1H), 5.65 (d, *J* = 5.9 Hz, 1H), 5.59 (d, *J* = 7.0 Hz, 1H), 4.44 (s, 2H), 2.40 (s, 3H), 1.40 (s, 9H). ^13^C {^1^H} NMR (75 MHz, CDCl_3_) δ 155.3, 144.7, 135.4, 133.0, 131.5, 129.7, 128.7, 124.0, 121.4, 118.5, 116.1, 110.1, 102.7, 80.7, 54.2, 53.4, 28.4, 21.7. HRMS (ESI) *m*/*z*: [M + H]^+^ calcd for C_22_H_26_ClN_2_O_4_S^+^, 449.1224; found, 449.1227. IR (cm^–1^): 3347, 1528, 1603, 1285.

#### *tert*-Butyl ((2-Phenyl-3-(tosylmethyl)-1*H*-indol-1-yl)­methyl)­carbamate (**14ds**)

The title compound was prepared from gramine **8s** (60.0 mg, 0.24 mmol, 1.2 equiv) and α-amido sulfone **1d** (57.0 mg, 0.2 mmol, 1 equiv) according to the general procedure. Column chromatography performed using gradient elution: 10:1 to 5:1 hexane/ethyl acetate. White solid, mp 166–168 °C. Yield: 75.6 mg, 0.154 mmol, 77%. ^1^H NMR (300 MHz, CDCl_3_) δ 7.68 (d, *J* = 8.2 Hz, 1H), 7.59 (d, *J* = 7.9 Hz, 2H), 7.51–7.35 (m, 4H), 7.35–7.27 (m, 2H), 7.23–7.11 (m, 3H), 7.10–6.99 (m, 1H), 5.31 (d, *J* = 6.4 Hz, 2H), 4.78 (s, 1H), 4.41 (s, 2H), 2.43 (s, 3H), 1.40 (s, 9H). ^13^C {^1^H} NMR (75 MHz, CDCl_3_) δ 144.4, 136.2, 130.6, 129.8, 129.7, 129.6, 129.2, 128.8, 128.8, 128.2, 123.2, 121.2, 120.3, 120.2, 110.8, 110.5, 110.1, 80.5, 54.5, 49.7, 28.4, 21.7. HRMS (ESI) *m*/*z*: [M + H]^+^ calcd for C_28_H_31_N_2_O_4_S^+^, 491.1926; found, 491.1928. IR (cm^–1^): 3313, 1512, 1633, 1289.

(**Reaction run at 6 mmol scale**) To a solution of gramine **8s** (1.80 g, 7.2 mmol, 1.2 equiv) in acetone (10 mL), α-amido sulfone **1d** (1.71 g, 6 mmol, 1 equiv) was added. Then, the reaction was cooled to 0 °C (bath temperature), and ethyl propiolate **12** (942 mg, 9.6 mmol, 1.6 equiv) was added dropwise. After stirring the reaction mixture at 0 °C for 60 min, the solvent was evaporated and the crude product was purified by flash column chromatography (gradient elution: 10:1 to 5:1 hexane/ethyl acetate). Compound **14ds** was obtained as a white solid. Yield: 2.53 g (5.16 mmol, 86%).

## Supplementary Material



## Data Availability

The data underlying this study are available in the published article and its online Supporting Information.

## Notes

Experimental procedures, temperature-dependent NMR experiments, spectroscopic data of products, and ^1^H NMR, ^13^C NMR, and ^19^F NMR spectra (PDF)
