# Supplementary material for: Deconstructing α‑Amidoalkyl Sulfones as Dual d‑Sulfonyl/a‑Azomethine Synthons: Synthesis of 3‑Sulfonylmethylindole Aminals
Source: J Org Chem. 2025 Aug 7;90(33):11910–22. doi: 10.1021/acs.joc.5c01392 (PMC12381929; doi:10.1021/acs.joc.5c01392)

SUPPORTING INFORMATION

Deconstructing α-Amidoalkyl Sulfones as Dual *d*-Sulfonyl/ *a*-Azomethine Synthons: Synthesis of 3-Sulfonylmethylindole Aminals

Guillermo Domínguez, Anje Mujika, Iker Hernández, Vadim A. Soloshonok, Aitor Landa, * and Mikel Oiarbide*

Table of Contents

1. Materials and general techniques 2
2. Synthesis of the starting materials 3
3. Behaviour of the gramine analogs 9, 10 and 11 (Table SI-1). 9
4. Control experiments. 10
5. ^{1}H NMR temperature coefficients (ΔσHN/ΔT) for compounds 8a and 13aa 20
6. ^{1}H, ^{13}C and ^{19}F NMR spectra. 23

1. Materials and general techniques

All non-aqueous reactions were performed under inert atmosphere using oven-dried glassware and were magnetically stirred. Yields refer to chromatographically purified and spectroscopically pure compounds, unless otherwise stated.

Heat requiring reactions were performed using a hotplate with an oil bath and a condenser. Reactions requiring low temperatures were performed using cooling bath circulators Huber T100E and isopropanol baths.

Organic layers were washed with aqueous phases dried over $MgSO_4$ and filtered through filter paper. Organic solvents were evaporated under reduced pressure using Büchi rotary evaporators. For trace solvent removal, a vacuum pump (≈0.5 mmHg) was applied.

Solvents and reagents

Solvents and reagents were purchased from different commercial suppliers (Across, Alfa Aesar, Fluka, TCI, Merck, Fluorochem, BLDpharm, etc.), stored as specified by the manufacturer and used without previous purification, unless otherwise stated.

Et_3N was purified by distillation over NaOH. When anhydrous solvents were required, they were dried following established procedures; CH_2Cl_2 and CH_3CN were dried over CaH_2 and THF was distilled over sodium/benzophenone. Analytical reagent grade Et_2O and toluene were used without further drying.

Chromatography

Reactions and flash chromatographic columns were monitored by thin layer chromatography (TLC) using Merck silica gel 60 F254 plates and visualized by fluorescence quenching under UV light. In addition, TLC plates were stained with a dipping solution of potassium permanganate (1g) in 100 mL of water (limited lifetime), followed by heating.

Chromatographic purification was performed on ROCC 60 silica gel 40-63 μm as stationary phase and a suitable mixture of solvents (see experimental procedures for more details) as eluent.

Melting points

Melting points were determined in open capillaries in a Stuart SHP3 melting point apparatus.

NMR spectra

^{1}H NMR, ^{13}C NMR and ^{19}F NMR spectra were recorded at 300 MHz, 75 MHz and 471 MHz respectively. Chemical shifts (δ) are quoted in parts per million referenced to $CDCl_3$ (δ = 7.26) and DMSO-d_6 (δ = 2.50), Methanol-d_4 (δ = 3.31) and Acetone-d_6 (δ = 2.05) for ^{1}H NMR and relative to the central resonances of $CDCl_3$ (δ = 77.2), DMSO-d_6 (δ = 39.5), and Acetone-d_6 (δ = 29.8) for ^{13}C NMR. For all ^{19}F NMR spectra fluorobenzene (C_6H_5F) was used as reference. The multiplicity of each signal is designated using the following abbreviations: s, singlet; d, doublet; dd, doublet of doublets; ddd, doublet of doublets of doublets; td, triplet of doublets; dtd, doublet of triplets of doublets; tdd, triplet of doublets of doublets; qd, quartet of doublets; t, triplet; dt, doublet of triplets; ddt, doublet of doublets of triplets; tt, triplet of triplets; q, quartet; dq, doublet of quartets; tq, triplet of quartets; m, multiplet. Coupling constants (*J*) are reported in Hertz (*Hz*). MestReNova Mnova 11.0 program was used to process and edit the registered spectra.

Mass spectra

MS spectra were recorded on an ESI-ion trap Mass spectrometer (Agilent 1100 series LC/MSD, SL model). Mass spectrometry analysis was performed in the General Research Service (SGIker) of the University of the Basque Country (UPV/EHU).

IR spectra

Infrared spectra were measured employing a Bruker ALPHA-P compact FT-IR spectrometer.

2. Synthesis of the starting materials

Starting indoles, carbamates and aldehydes were purchased from commercial suppliers and used without further purification, unless otherwise stated.

2.1. Synthesis of α-amido sulfones 1

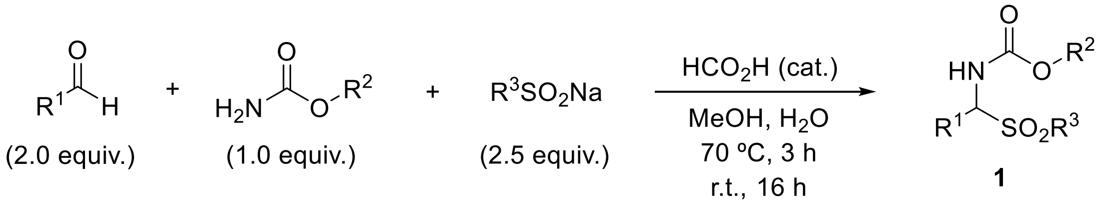

To a solution of the corresponding carbamate (50 mmol) in water (100 mL) the corresponding sodium sulfinate (124 mmol), and a solution of the aldehyde (100 mmol)

in methanol (50 mmol) were added. After that, formic acid (20 mL) was added, and the mixture was stirred for 3 hours at 70 ºC and was left stirring at room temperature overnight. Afterwards, the mixture was filtered under vacuum and the obtained solid was washed several times with water, hexane and Et_2O. The solid was dissolved in CH_2Cl_2 (50 mL) and dried with $MgSO_4$. Finally, the solvent was evaporated under reduced pressure to yield **1** as white solids.[1]

***tert*-Butyl (phenyl(tosyl)methyl) carbamate (1a)**

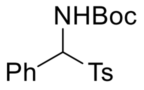

The title compound was prepared from tert-butyl carbamate (5.9 g, 50 mmol, 1.0 equiv.), sodium *p*-toluene sulfinate (22.1 g, 124 mmol, 2.5 equiv.), and benzaldehyde (10.6 g, 100 mmol, 2.0 equiv.) according to the general procedure. White solid. Yield: 13.37 g, 37.0 mmol, 74 %. ^{1}H NMR (300 MHz, $CDCl_3$) δ 7.78 (d, *J* = 8.0 Hz, 2H), 7.42 (s, 5H), 7.32 (d, *J* = 8.0 Hz, 2H), 5.88 (d, *J* = 10.9 Hz, 1H), 5.69 (d, *J* = 11.0 Hz, 1H), 2.43 (s, 3H), 1.27 (s, 9H). All spectroscopy data was coincident with those previously reported in the literature.[2]

Benzyl (phenyl(tosyl)methyl) carbamate (1a')

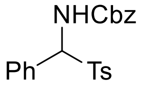

The title compound was prepared from benzyl carbamate (7.6 g, 50 mmol, 1.0 equiv.), sodium p-toluene sulfinate (22.1 g, 124 mmol, 2.5 equiv.), and benzaldehyde (10.6 g, 100 mmol, 2.0 equiv.) according to the general procedure. White solid. Yield: 16.02 g, 40.5 mmol, 81 %. ^{1}H NMR (300 MHz, $CDCl_3$) δ 7.75–7.65 (dd, 2H), 7.44–7.31 (m, 8H), 7.23 (dd, 2H), 5.91 (s, 2H), 4.97 (s, 2H), 2.43 (s, 3H).[2]

***tert*-Butyl (1-tosylpropyl) carbamate (1b)**

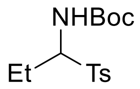

The title compound was prepared from tert-butyl carbamate (5.9 g, 50 mmol, 1.0 equiv.), sodium *p*-toluene sulfinate (22.1 g, 124 mmol, 2.5 equiv.), and propionaldehyde (5.8 g, 100 mmol, 2.0 equiv.) according to the general procedure. White solid. Yield: 9.72 g, 31.0 mmol, 62 %. ^{1}H NMR (300 MHz, $CDCl_3$) δ 7.78 (d, *J* = 8.2 Hz, 2H), 7.32 (d, *J* = 8.1 Hz, 2H), 4.92 (d, *J* = 10.9 Hz, 1H), 4.73 (td, *J* = 10.7, 3.6 Hz, 1H), 2.41 (s, 3H), 2.33–1.71 (m, 2H), 1.23 (s, 9H), 1.08 (t, *J* = 7.4 Hz, 3H). All spectroscopy data were coincident with those previously reported in the literature.[3]

[1] Pearson, W. H.; Lindbeck, A. C.; Kampf, J. W. *J. Am. Chem. Soc*. **1993**, *115*, 2622–2636.
[2] Starck, J.; Michelet, V. *Org. Lett.* **2020**, *22*, 7064–7067.
[3] Schwieter, K. E. ; Johnston, J. N. *ACS Catal.* **2015**, *5*, 6559–6562.

tert-Butyl (cyclohexyl(phenylsulfonyl)methyl) carbamate (1c)

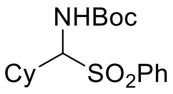

The title compound was prepared from tert-butyl carbamate (5.9 g, 50 mmol, 1.0 equiv.), sodium phenyl sulfinate (20.4 g, 124 mmol, 2.5 equiv.) and cyclohexanecarbaldehyde (11.2 g, 100 mmol, 2.0 equiv.) according to the general procedure. White solid. Yield: 14.0 g, 39.5 mmol, 79 %. ^{1}H NMR (300 MHz, $CDCl_3$) δ 7.97–7.82 (m, 2H), 7.70–7.44 (m, 3H), 5.14 (d, *J* = 11.2 Hz, 1H), 4.93–4.49 (m, 1H), 2.46 (td, *J* = 11.8, 3.3 Hz, 1H), 2.14 (d, *J* = 12.6 Hz, 1H), 1.72 (d, *J* = 31.6 Hz, 5H), 1.39–1.32 (m, 1H), 1.22 (s, 9H), 1.16–1.03 (m, 3H). All spectroscopy data were coincident with those previously reported in the literature.[4]

tert-Butyl (tosylmethyl)carbamate (1d)

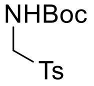

The title compound was prepared from tert-butyl carbamate (5.9 g, 50 mmol, 1.0 equiv.), sodium *p*-toluene sulfinate (22.1 g, 124 mmol, 2.5 equiv.), and formaldehyde (37 % w/v in H_2O / 7.8 ml, 100 mmol, 2.0 equiv.) according to the general procedure. White solid. Yield: 10.27 g, 36.0 mmol, 72 %. ^{1}H NMR (300 MHz, $CDCl_3$) δ 7.79 (d, *J* = 8.1 Hz, 2H), 7.34 (d, *J* = 8.0 Hz, 2H), 5.31 (d, *J* = 9.0 Hz, 1H), 4.49 (d, *J* = 7.0 Hz, 2H), 2.43 (s, 3H), 1.27 (s, 9H). All spectroscopy data were coincident with those previously reported in the literature.[5]

tert-Butyl ((4-chlorophenyl)(tosyl)methyl)carbamate (1e)

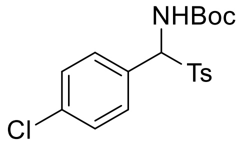

The title compound was prepared from tert-butyl carbamate (5.9 g, 50 mmol, 1.0 equiv.), sodium *p*-toluene sulfinate (22.1 g, 124 mmol, 2.5 equiv.), and 4-chlorobenzaldehyde (14.1 g, 100 mmol, 2.0 equiv.) according to the general procedure. White solid. Yield: 15.2 g, 38.5 mmol, 77 %. ^{1}H NMR (300 MHz, $CDCl_3$) δ 7.85 – 7.72 (m, 2H), 7.38 (s, 4H), 7.34 (d, *J* = 8.0 Hz, 2H), 5.86 (d, *J* = 10.9 Hz, 1H), 5.66 (d, *J* = 10.8 Hz, 1H), 2.43 (s, 3H), 1.26 (s, 9H). All spectroscopy data were coincident with those previously reported in the literature.[6]

[4] Petrini, M.; Mecozzi, T. *J. Org. Chem.* **1999**, *64*, 8970–8972
[5] Zwierzak, A. *Tetrahedron Lett.* **2002**, *43*, 1079–1080.
[6] Bull, J. A.; Boultwood, T.; Taylor, T. A. *Chem. Commun.* **2012**, *48*, 12246–12248

tert-Butyl ((4-methoxyphenyl)(tosyl)methyl)carbamate (1f)

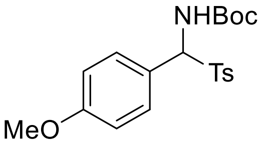

The title compound was prepared from tert-butyl carbamate (5.9 g, 50 mmol, 1.0 equiv.), sodium *p*-toluene sulfinate (22.1 g, 124 mmol, 2.5 equiv.), and 4-methoxybenzaldehyde (13.6 g, 100 mmol, 2.0 equiv.) according to the general procedure. White solid. Yield: 14.3 g, 36.5 mmol, 73 %. ^{1}H NMR (300 MHz, $CDCl_3$) δ 7.85 – 7.69 (m, 2H), 7.43 – 7.28 (m, 4H), 6.98 – 6.89 (m, 2H), 5.83 (d, *J* = 10.8 Hz, 1H), 5.65 (d, *J* = 10.8 Hz, 1H), 3.82 (s, 3H), 2.42 (s, 3H), 1.26 (s, 9H). All spectroscopy data were coincident with those previously reported in the literature.[7]

2.2. Synthesis of gramines 8 and 15

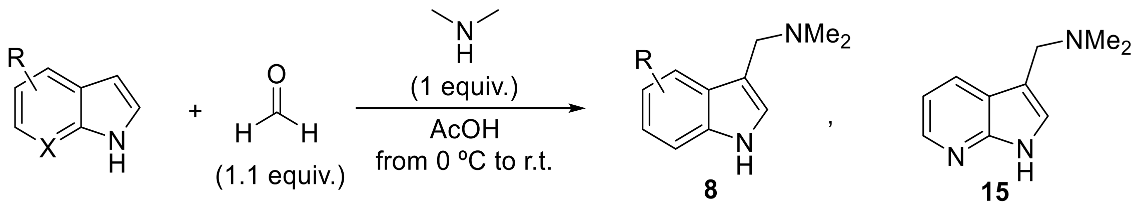

R:

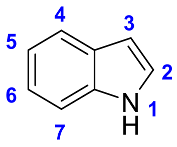

8a R = H
8b R = 5-Me
8c R = 5-MeO
8d R = 6-OBn
8e R = 4-Cl
8f R = 5-Cl
8g R = 6-Cl
8h R = 7-Cl
8i R = 6-F
8j R = 5,6-F
8k R = 5-CF_3
8l R = 5-CO_2Me
8m R = 5-CN
8n R = 6-CN
8o R = 6-NO_2
8p R =5-Bpin
8r R = 2-Me
8s R = 2-Ph
8t R = N-Me

To a solution of dimethylamine (2.0 M in THF, 10.2 mL, 20.4 mmol) in a 50 mL round bottom flask at 0 ºC, formaldehyde (37 % w/v in H_2O, 1.725 mL, 22.4 mmol, 1.1 equiv.) and glacial acetic acid (1.8 mL, 47.5 mmol, 2.3 equiv.) were added and the solution was stirred for 10 min. To this mixture, the corresponding indole (20.4 mmol) dissolved in glacial acetic acid (2.4 mL) was added dropwise. Once the addition was completed, the reaction mixture was slowly brought to room temperature and then stirred overnight. The reaction solution was then poured into water and brought to an alkaline pH by the addition of aqueous NaOH 10% solution. The resulting precipitate was filtered off, washed with water and hexane and dried under reduced pressure until all solvents and water were removed. All spectroscopy data of compounds **8a, 8b, 8c, 8d, 8e, 8f, 8g, 8h, 8i, 8j, 8k, 8l, 8m, 8o, 8p, 8r, 8s** were coincident with those previously reported in the literature.[8]

---

[7] Wenzel, A.G.; Jacobsen, E. N. *J. Am. Chem. Soc.* **2002**, *124*, 12964-12965.

[8] a) Pillaiyar, T.; Gorska, E.; Schnakenburg, G.; Müller, C. E. *J. Org. Chem.* **2018**, *83*, 17, 9902–9913. b) Bandini, M.; Eichholzer, A. *Angew. Chem. Int. Ed.* **2009**, *48*, 9533–9537.

3-((Dimethylamino)methyl)-1*H*-indole-6-carbonitrile (8n)

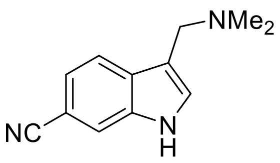

The title compound was prepared from 1*H*-indole-6-carbonitrile (2.9 g, 20.4 mmol, 1.0 equiv.) according to the general procedure. Pale yellow solid. Yield: 3.1 g, 15.5 mmol, 76%. ^{1}H NMR (300 MHz, $CDCl_3$) δ 8.68 (s, 1H), 7.79–7.54 (m, 2H), 7.34 (dt, *J* = 7.2, 1.3 Hz, 2H), 3.62 (s, 2H), 2.27 (d, *J* = 0.9 Hz, 6H). All spectroscopy data were coincident with those previously reported in the literature.[9]

***N,N*-Dimethyl-1-(1-methyl-1*H*-indol-3-yl)methanamine (8t)**

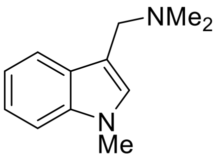

The title compound was prepared from 1-methyl-1*H*-indole (2.7 g, 20.4 mmol, 1.0 equiv.) according to the general procedure. White solid. Yield: 3.1 g, 16.53 mmol, 81%. ^{1}H NMR (300 MHz, $CDCl_3$) δ 7.69 (dd, *J* = 8.0, 1.1 Hz, 1H), 7.35–7.18 (m, 2H), 7.12 (ddt, *J* = 7.9, 6.9, 0.9 Hz, 1H), 7.00 (s, 1H), 3.77 (d, *J* = 0.6 Hz, 3H), 3.62 (s, 2H), 2.28 (s, 6H). All spectroscopy data were coincident with those previously reported in the literature.[10]

***N,N*-Dimethyl-1-(1*H*-pyrrolo[2,3-b]pyridin-3-yl)methanamine (15)**

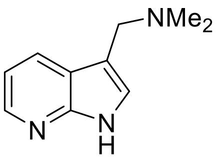

The title compound was prepared from 1*H*-pyrrolo[2,3-b]pyridine (2.4 g, 20.4 mmol, 1.0 equiv.) according to the general procedure. White solid. Yield: 2.43 g, 13.87 mmol, 68%. ^{1}H NMR (300 MHz, Methanol-*d*$_4$) δ 7.99 (dd, *J* = 4.7, 1.6 Hz, 1H), 7.78 (dd, *J* = 7.8, 1.7 Hz, 1H), 7.14 (s, 1H), 6.94–6.64 (m, 1H), 3.33 (s, 2H), 1.93 (s, 6H). ^{13}C {^{1}H} NMR (75 MHz, DMSO-*d*$_6$) δ 148.8, 142.5, 127.3, 125.0, 119.8, 115.1, 110.5, 54.3, 44.8. All spectroscopy data were coincident with those previously reported in the literature.[11]

2.3. Synthesis of the gramine analogs 9, 10 and 11.

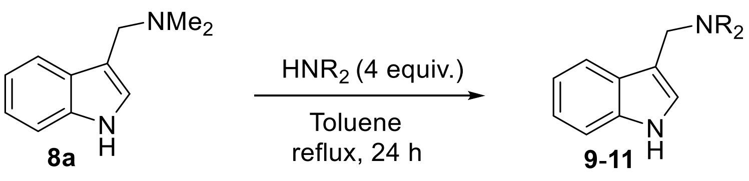

[9] Hernández, I.; Domínguez, G.; Soloshonok, V. A.; Landa, A.; Oiarbide, M. *J. Org. Chem.* **2024**, *89*, 23, 17291–17309.
[10] Stanek, F.; Pawłowski, R.; Mlynarski, J.; Stodulski, M. *Eur. J. Org. Chem.* **2018**, 6624–6628.
[11] Garder, E.D.; Johnson, B. P.; Dimas, D. A.; McClurg, H. E.; Severance, Z. C.; Burgett, A. W.; Singh, S. *ChemCatChem*. **2023**, *15*, e202300650.

To a solution of gramine **8a** (1.74 g, 10 mmol, 1 equiv.) in dry toluene (75 mL) at room temperature the corresponding amine (40 mmol, 4 equiv.) was added, and the solution was refluxed overnight. Once the reaction was completed, the solvent was removed in vacuo and the obtained crude material was purified by recrystallization in Et_2O.[12]

3-(Pyrrolidin-1-ylmethyl)-1*H*-indole (9)

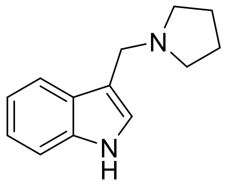

The title compound was prepared from pyrrolidine (3.3 mL, 40 mmol, 4.0 equiv.) according to the general procedure. White solid. Yield: 1.88 g, 9.41 mmol, 94%. ^{1}H NMR (300 MHz, $CDCl_3$) δ 8.23 (s, 1H), 7.72 (d, J = 7.7 Hz, 1H), 7.34 (dd, J = 7.9, 2.2 Hz, 1H), 7.24–7.06 (m, 3H), 3.85 (d, *J* = 1.8 Hz, 2H), 2.60 (tq, *J* = 4.2, 2.2 Hz, 4H), 1.92–1.68 (m, 4H). All spectroscopy data were coincident with those previously reported in the literature.[13]

3-(Piperidin-1-ylmethyl)-1*H*-indole (10)

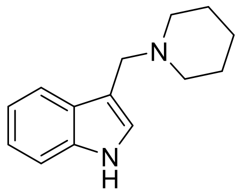

The title compound was prepared from piperidine (3.95 mL, 40 mmol, 4.0 equiv.) according to the general procedure. White solid. Yield: 2.07 g, 9.7 mmol, 97%. ^{1}H NMR (300 MHz, $CDCl_3$) δ 8.29 (s, 1H), 7.74 (d, *J* = 7.7 Hz, 1H), 7.35 (d, *J* = 7.9 Hz, 1H), 7.22–7.07 (m, 3H), 3.72 (s, 2H), 2.48 (t, *J* = 5.3 Hz, 4H), 1.58 (t, *J* = 5.7 Hz, 4H), 1.42 (h, *J* = 5.7, 4.8 Hz, 2H). All spectroscopy data were coincident with those previously reported in the literature.[11]

4-((1*H*-Indol-3-yl)methyl)morpholine (11)

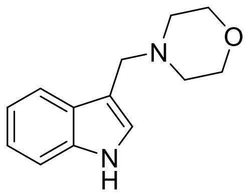

The title compound was prepared from morpholine (3.45 mL, 40 mmol, 4.0 equiv.) according to the general procedure. White solid. Yield: 1.97 g, 9.1 mmol, 91%. ^{1}H NMR (300 MHz, $CDCl_3$) δ 8.07 (s, 1H), 7.77 (dd, *J* = 7.8, 1.3 Hz, 1H), 7.41–7.33 (m, 1H), 7.29–7.09 (m, 3H), 3.71 (q, *J* = 4.2, 3.6 Hz, 6H), 2.51 (t, *J* = 4.7 Hz, 4H). All spectroscopy data were coincident with those previously reported in the literature.[11]

---

[12] Adapted from: Yin, X.-J.; Huang, X.-Y.; Ma, Y.-B.; Geng, C.-A.; Li, T.-Z.; Chen, X.-L.; Yang, T.-H.; Zhou, J.; Zhang, X.-M.; Chen, J.-J. *J. Asian Nat. Prod. Res*. **2017**, *19*, 610–622.
[13] Sakai, N.; Shimamura, K.; Ikeda, R.; Konakahara, T. *J. Org. Chem*. **2010**, *75*, 3923–3926.

3. Behaviour of the gramine analogs 9, 10 and 11 (Table SI-1).

Table SI-1. Comparison of gramines with varying NR_2 groups on the coupling reaction with α-amido sulfone 1a.[a]

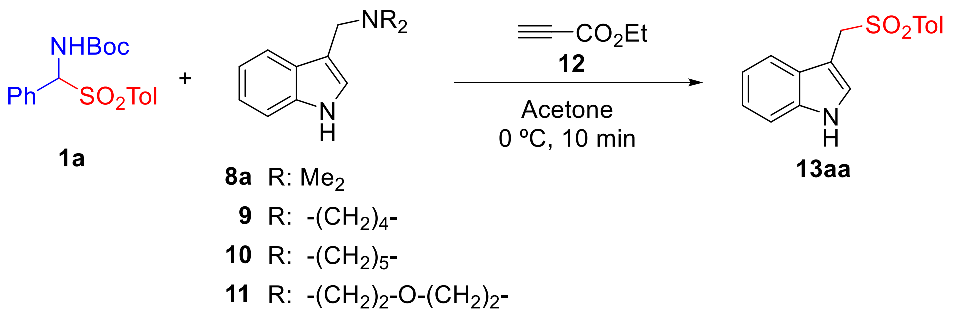

Entry	Gramine	Conv. (%)[b]	Yield (%)[c]
1	**8a**	99	98
2	**9**	96	94
3	**10**	97	96
4	**11**	60	58

[a] Reactions conducted at 0.1 mmol scale using 2.0 mL of acetone as solvent. Mol ratio of **1a**/gramine/**12** 1.0/2.0/1.6. [b] Conversion determined by ^{1}H NMR. [c] Yield of product **13aa** isolated after column chromatography

4. Control experiments.

5.1) Gramine **8a** + α-amido sulfone **1a**

To a solution containing α-amido sulfone **1a** (36.1 mg, 0.1 mmol, 1 equiv.) in acetone at 0 ºC, gramine **8a** was added (17.4 mg, 0.1 mmol, 1 equiv.). The solution was stirred for 10 min. Then an aliquot was taken, the solvent removed under vacuum and the residue analyzed by ^{1}H NMR (300 MHz, $CDCl_3$).

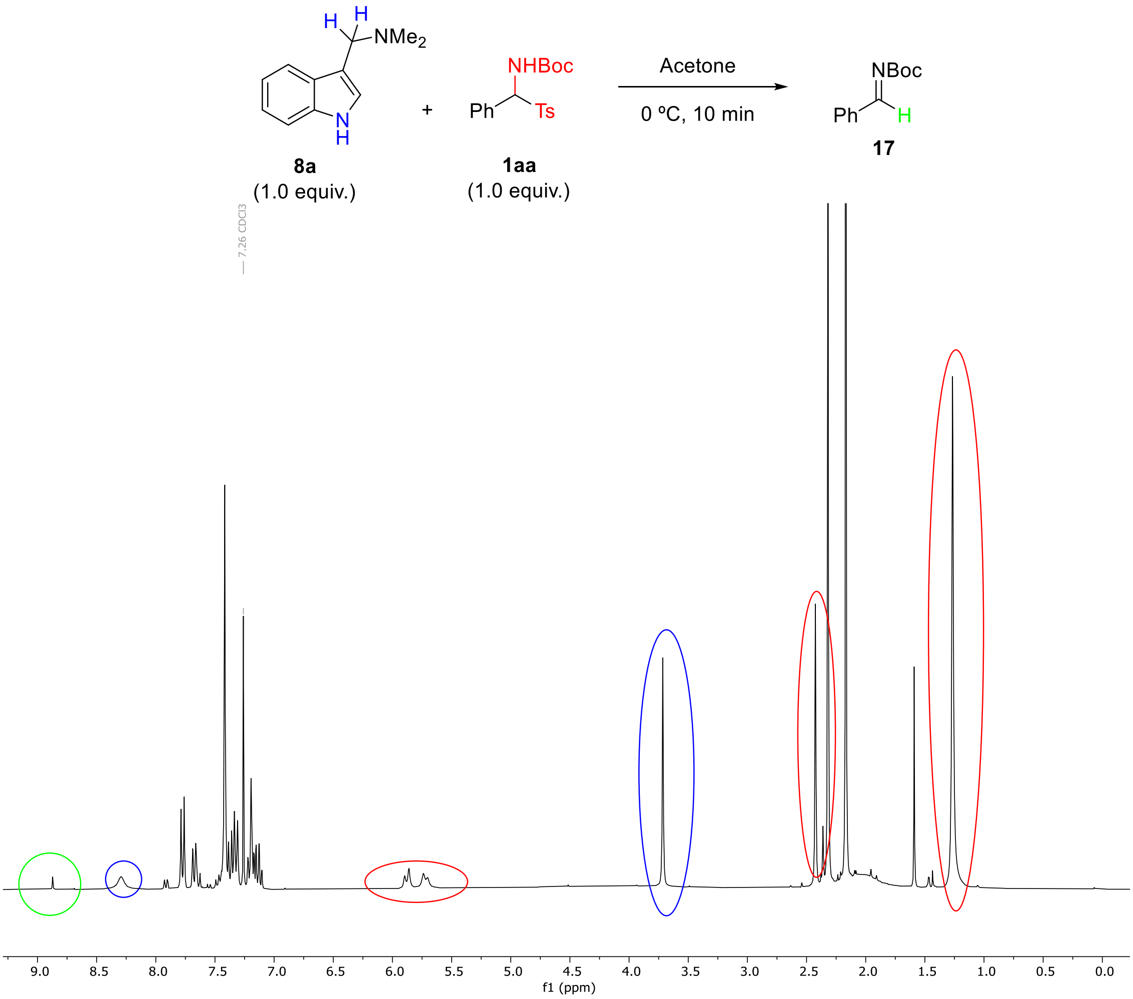

5.2) Enamine **20** + α-amido sulfone **1aa**

To a solution containing α-amido sulfone **1a** (36.1 mg, 0.1 mmol, 1 equiv.) in acetone at 0 ºC, enamine **20** was added (14.3 mg, 0.1 mmol, 1 equiv.). The solution was stirred for 10 min. Then an aliquot was taken, the solvent removed under vacuum and the residue analyzed by ^{1}H NMR (300 MHz, $CDCl_3$).

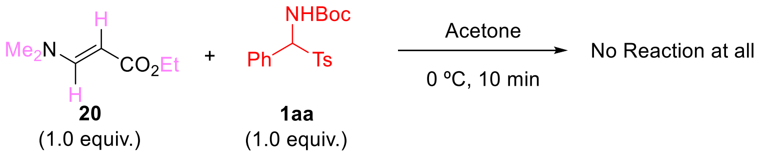

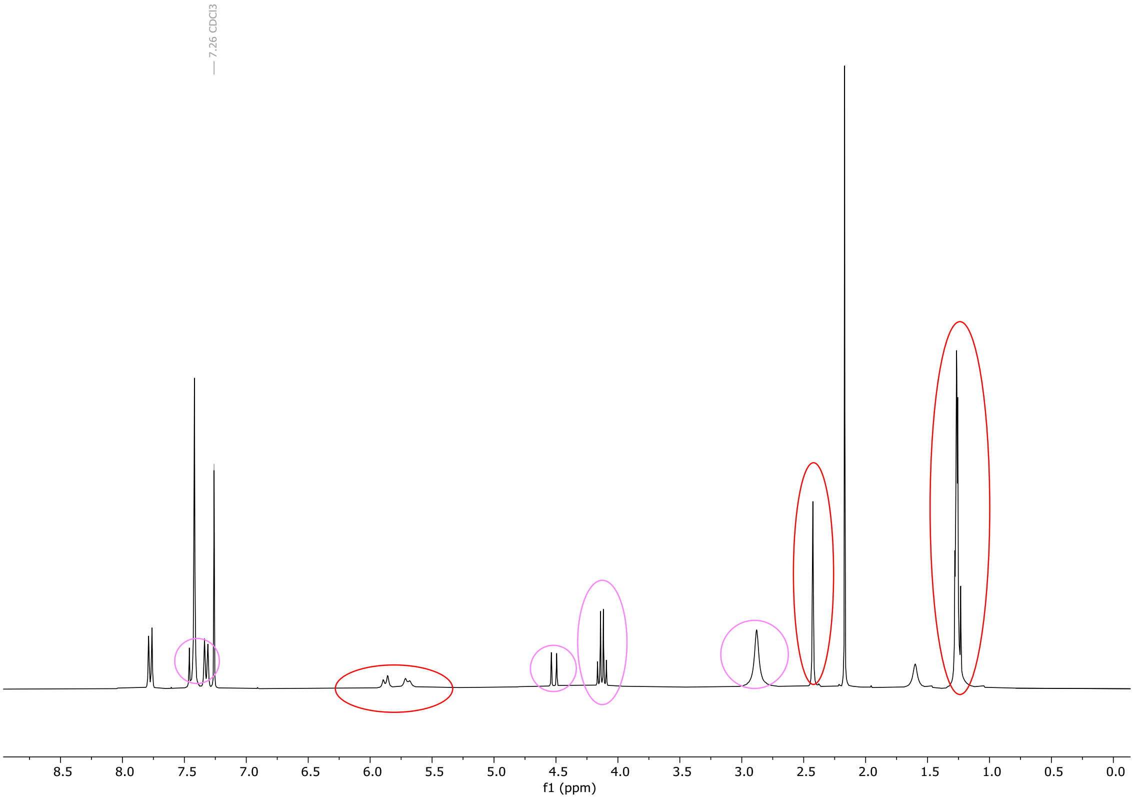

5a) 3-Sulfonylmethylindole **13aa** + imine **17**

To a solution containing 3-sulfonylmethylindole **13aa** (28.5 mg, 0.1 mmol, 1 equiv.) in acetone at 0 ºC, imine **17** (20.5 mg, 0.1 mmol, 1 equiv.) was added. The solution was stirred for 10 min. Then an aliquot was taken, the solvent removed under vacuum and the residue analyzed by ^{1}H NMR (300 MHz, $CDCl_3$).

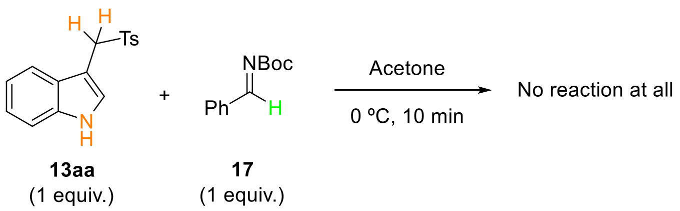

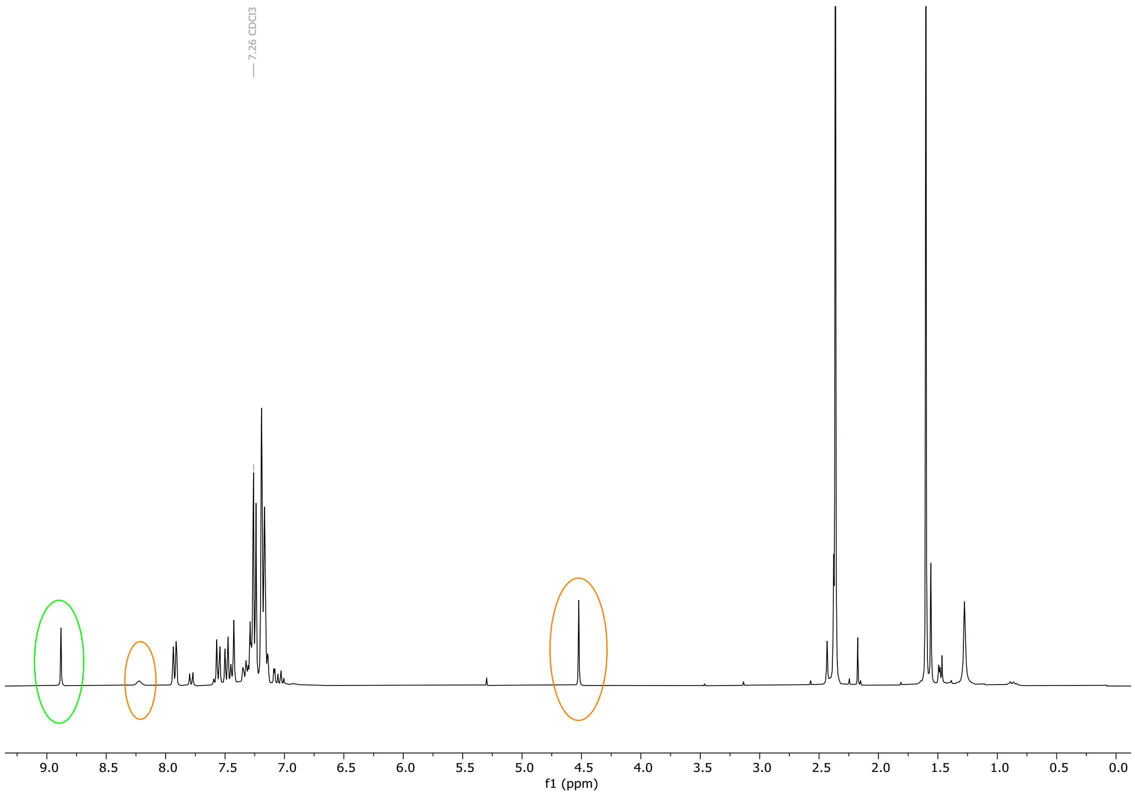

5b) 3-Sulfonylmethylindole **13aa** + imine **17** + gramine **8a**

To a solution containing 3-sulfonylmethylindole **13aa** (28.5 mg, 0.1 mmol, 1 equiv.) in acetone at 0 ºC, imine **17** (20.5 mg, 0.1 mmol, 1 equiv.) and gramine **8a** (17.4 mg, 0.1 mmol, 1 equiv.) were added. The solution was stirred for 10 min. Then an aliquot was taken, the solvent removed under vacuum and the residue analyzed by ^{1}H NMR (300 MHz, $CDCl_3$).

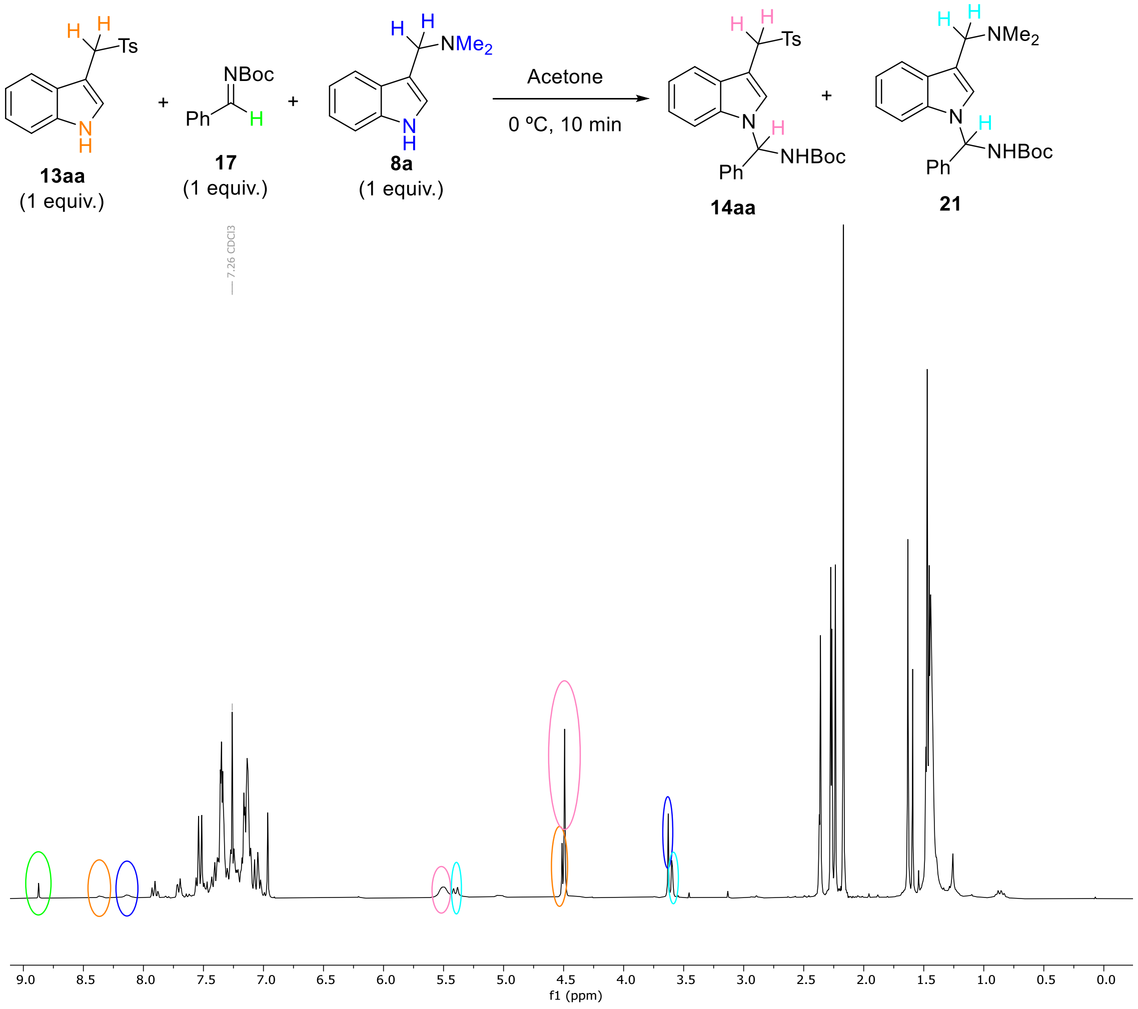

5c) 3-Sulfonylmethylindole **13aa** + imine **17** + gramine **18**

To a solution containing 3-sulfonylmethylindole **13aa** (28.5 mg, 0.1 mmol, 1 equiv.) in acetone at 0 ºC, imine **17** (20.5 mg, 0.1 mmol, 1 equiv.) and gramine **18** (18.8 mg, 0.1 mmol, 1 equiv.) were added. The solution was stirred for 10 min. Then an aliquot was taken, the solvent removed under vacuum and the residue analyzed by ^{1}H NMR (300 MHz, $CDCl_3$).

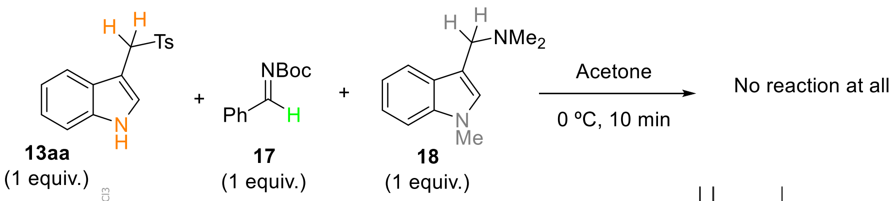

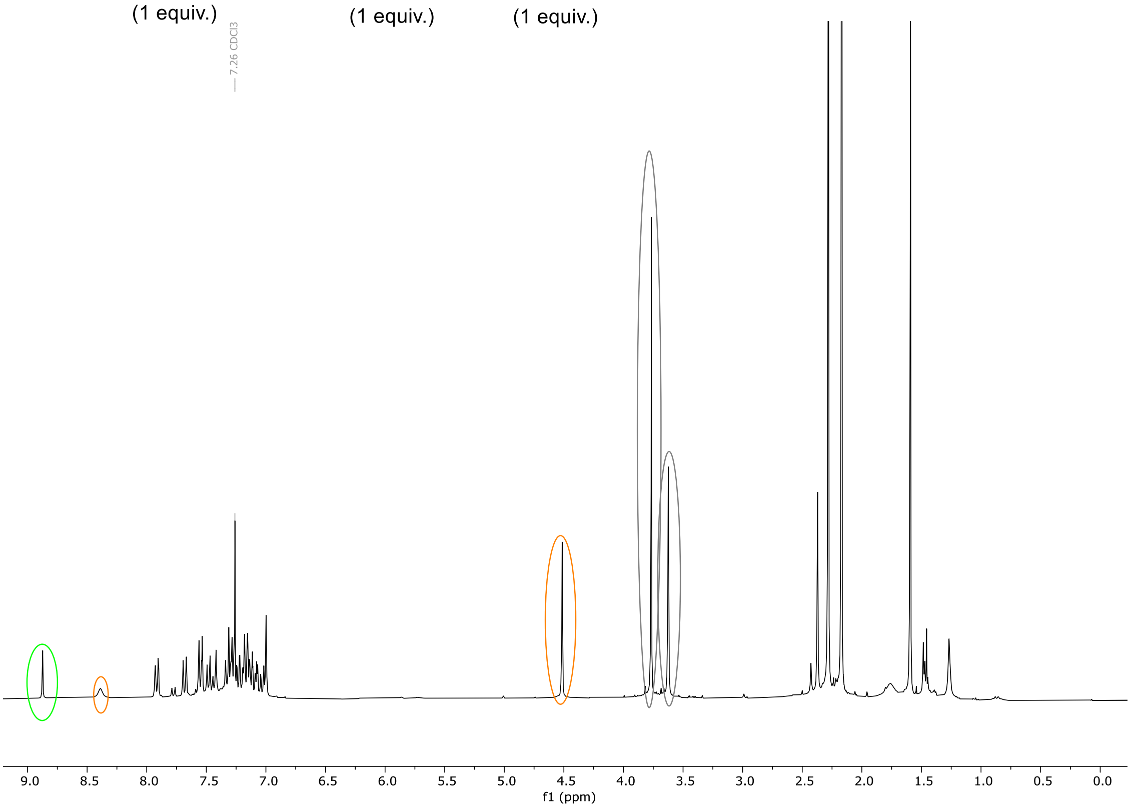

5d) 3-Sulfonylmethylindole **13aa** + imine **17** + indole **19**

To a solution containing 3-sulfonylmethylindole **13aa** (28.5 mg, 0.1 mmol, 1 equiv.) and indole **19** (13.1 mg, 0.1 mmol, 1 equiv.) in acetone and at 0 ºC, imine **17** (20.5 mg, 0.1 mmol, 1 equiv.) was added. The solution was stirred for 10 min. Then an aliquot was taken, the solvent removed under vacuum and the residue analyzed by ^{1}H NMR (300 MHz, $CDCl_3$).

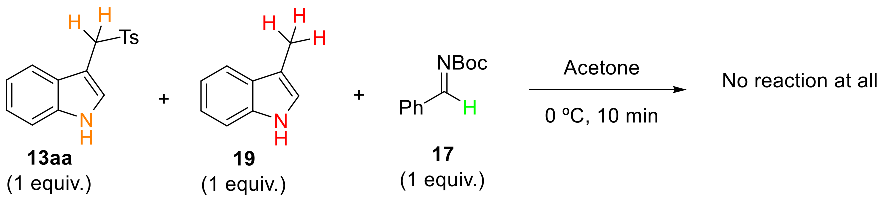

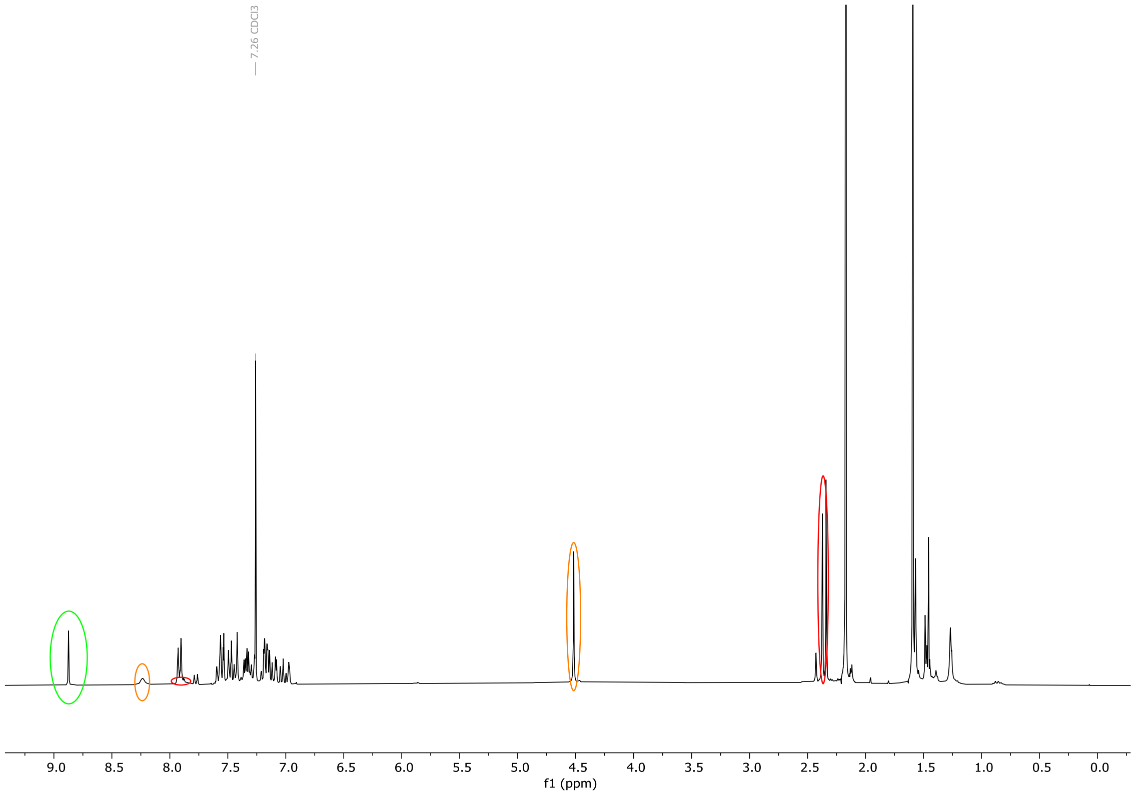

5e) 3-sulfonylmethylindole **13aa** + imine **17** + triethylamine

To a solution containing 3-sulfonylmethylindole **13aa** (28.5 mg, 0.1 mmol, 1 equiv.) in acetone and at 0 ºC, imine **17** (20.5 mg, 0.1 mmol, 1 equiv.) and triethylamine (2 mg, 0.02 mmol, 0.2 equiv.) were added. The solution was stirred for 10 min. Then an aliquot was taken, the solvent removed under vacuum and the residue analyzed by ^{1}H NMR (300 MHz, $CDCl_3$).

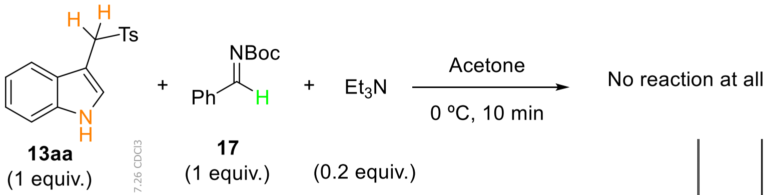

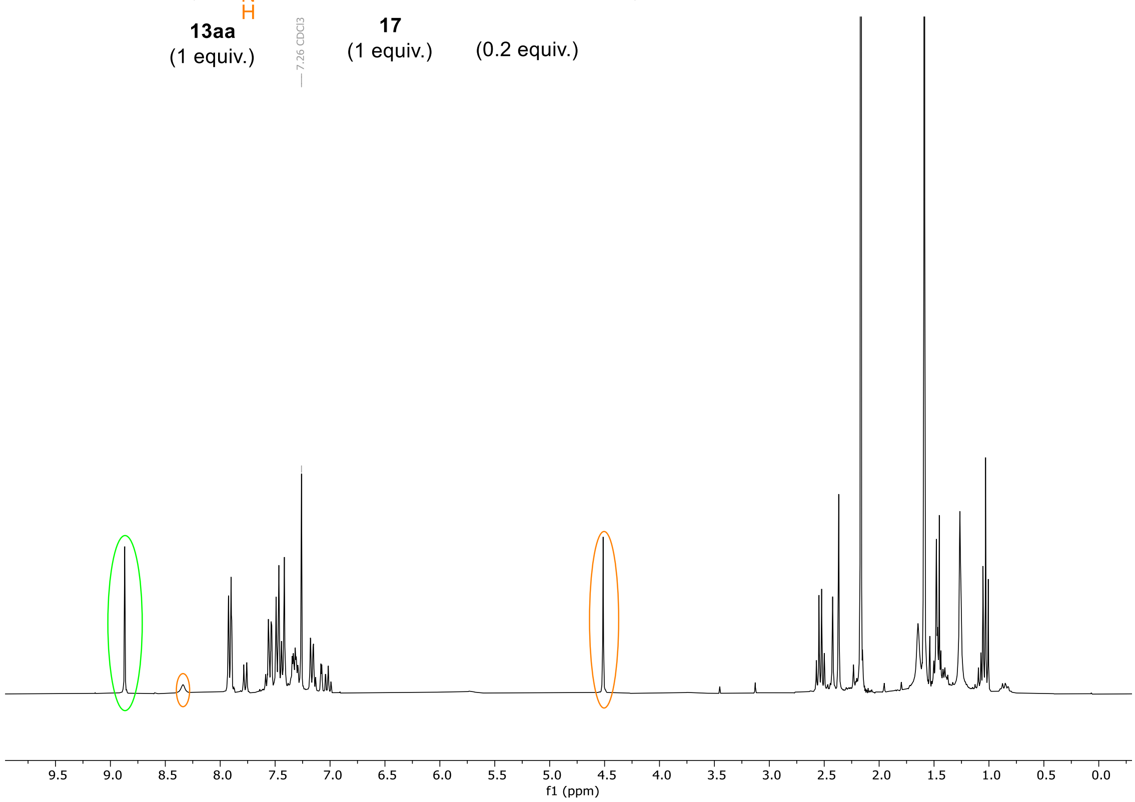

5f) 3-sulfonylmethylindole **13aa** + imine **17** + **C1** catalyst

To a solution containing 3-sulfonylmethylindole **13aa** (28.5 mg, 0.1 mmol, 1 equiv.) in toluene and at 0 ºC, imine **17** (20.5 mg, 0.1 mmol, 1 equiv.) and Takemoto's catalyst **C1** (9.0 mg, 0.02 mmol, 0.2 equiv.) were added. The solution was stirred for 10 min. Then an aliquot was taken, the solvent removed under vacuum and the residue analyzed by ^{1}H NMR (300 MHz, $CDCl_3$).

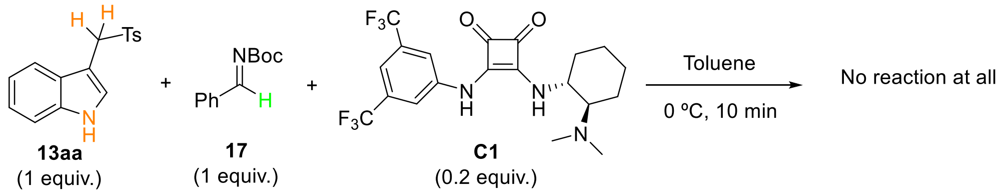

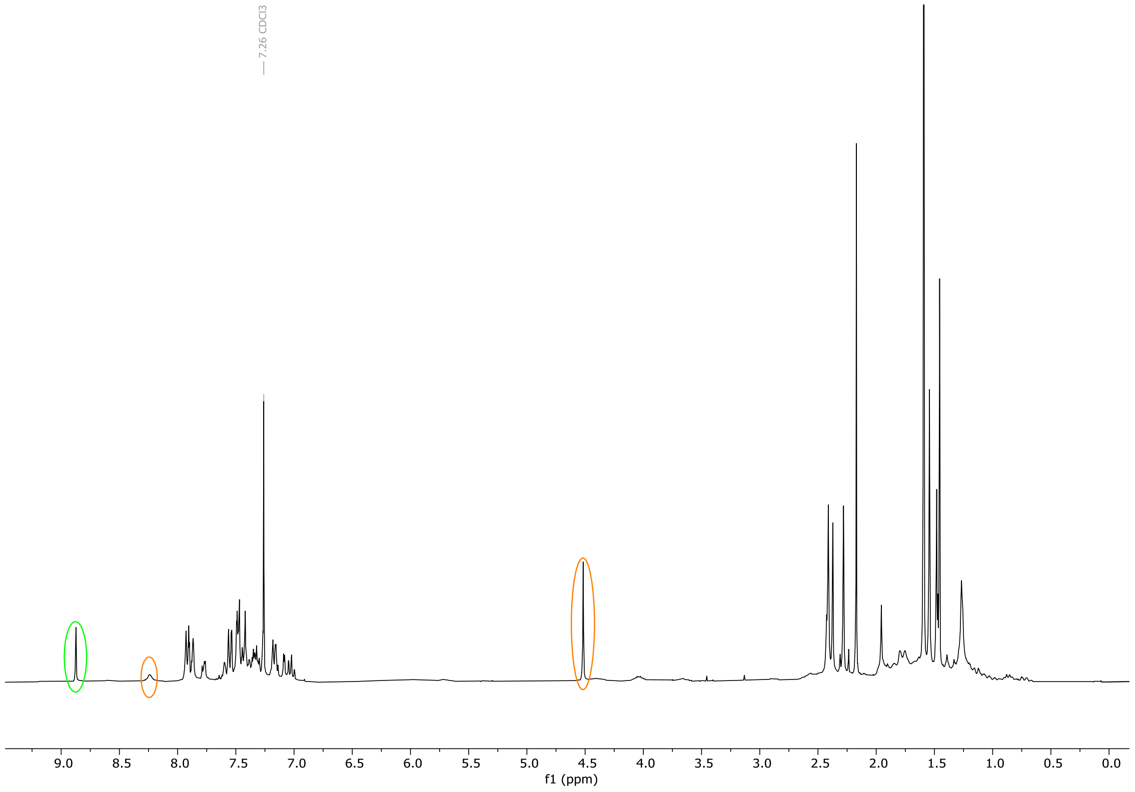

5g) 3-Sulfonylmethylindole **13aa** + imine **17** + gramine **8a** + **C1** catalyst

To a solution containing 3-sulfonylmethylindole **13aa** (28.5 mg, 0.1 mmol, 1 equiv.) in toluene and at 0 ºC, imine **17** (20.5 mg, 0.1 mmol, 1 equiv.), gramine **8a** (17.4 mg, 0.1 mmol, 1 equiv.) and catalyst **C1** (9.0 mg, 0.02 mmol, 0.2 equiv.) were added. The solution was stirred for 10 min. Then an aliquot was taken, the solvent removed under vacuum and the residue analyzed by ^{1}H NMR (300 MHz, $CDCl_3$).

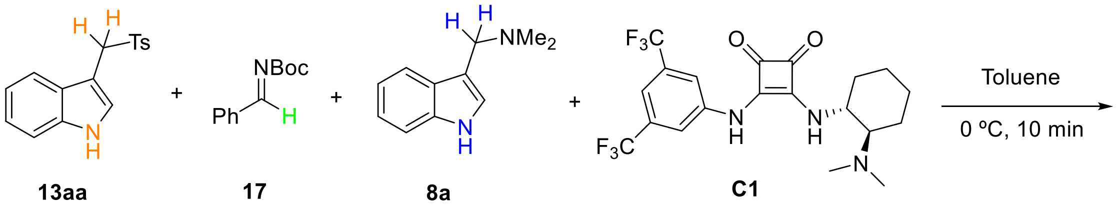

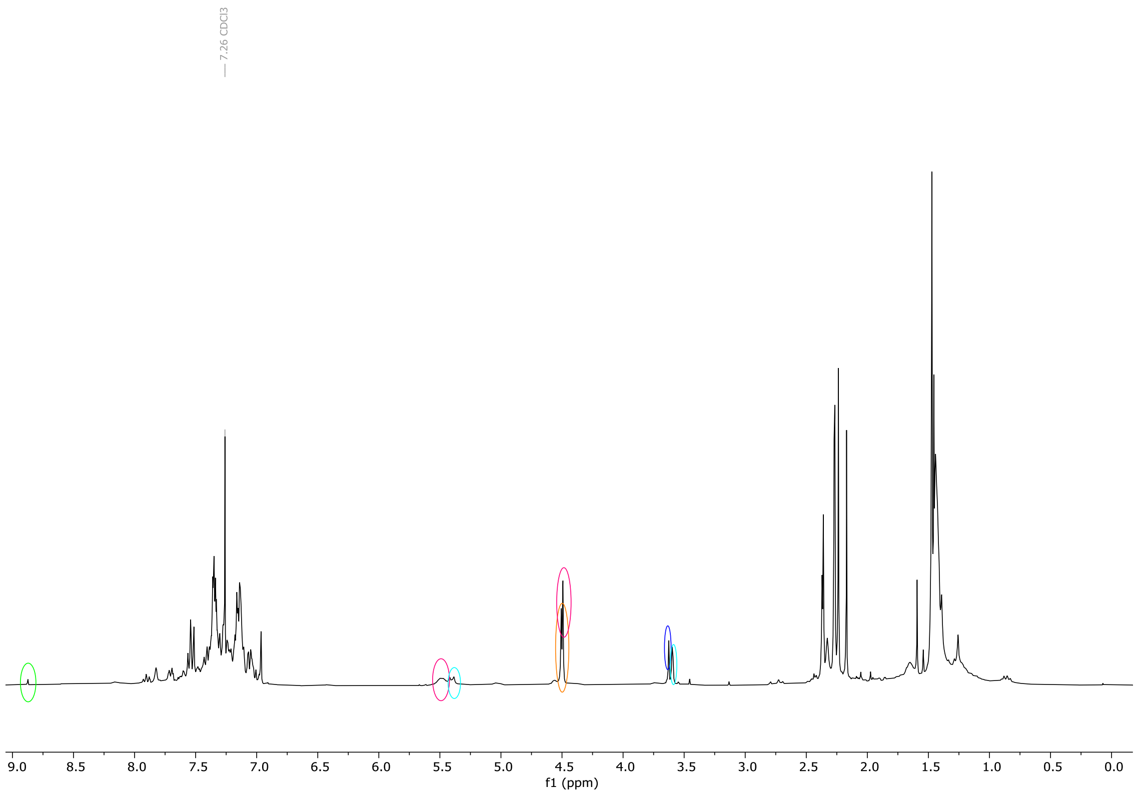

5h) 3-sulfonylmethylindole **13aa** + imine **17** + enamine **20**

To a solution containing 3-sulfonylmethylindole **13aa** (28.5 mg, 0.1 mmol, 1 equiv.) in acetone and at 0 ºC, imine **17** (20.5 mg, 0.1 mmol, 1 equiv.) and enamine **20** (14.3 mg, 0.1 mmol, 1 equiv.) were added. The solution was stirred for 10 min. Then an aliquot was taken, the solvent removed under vacuum and the residue analyzed by ^{1}H NMR (300 MHz, $CDCl_3$).

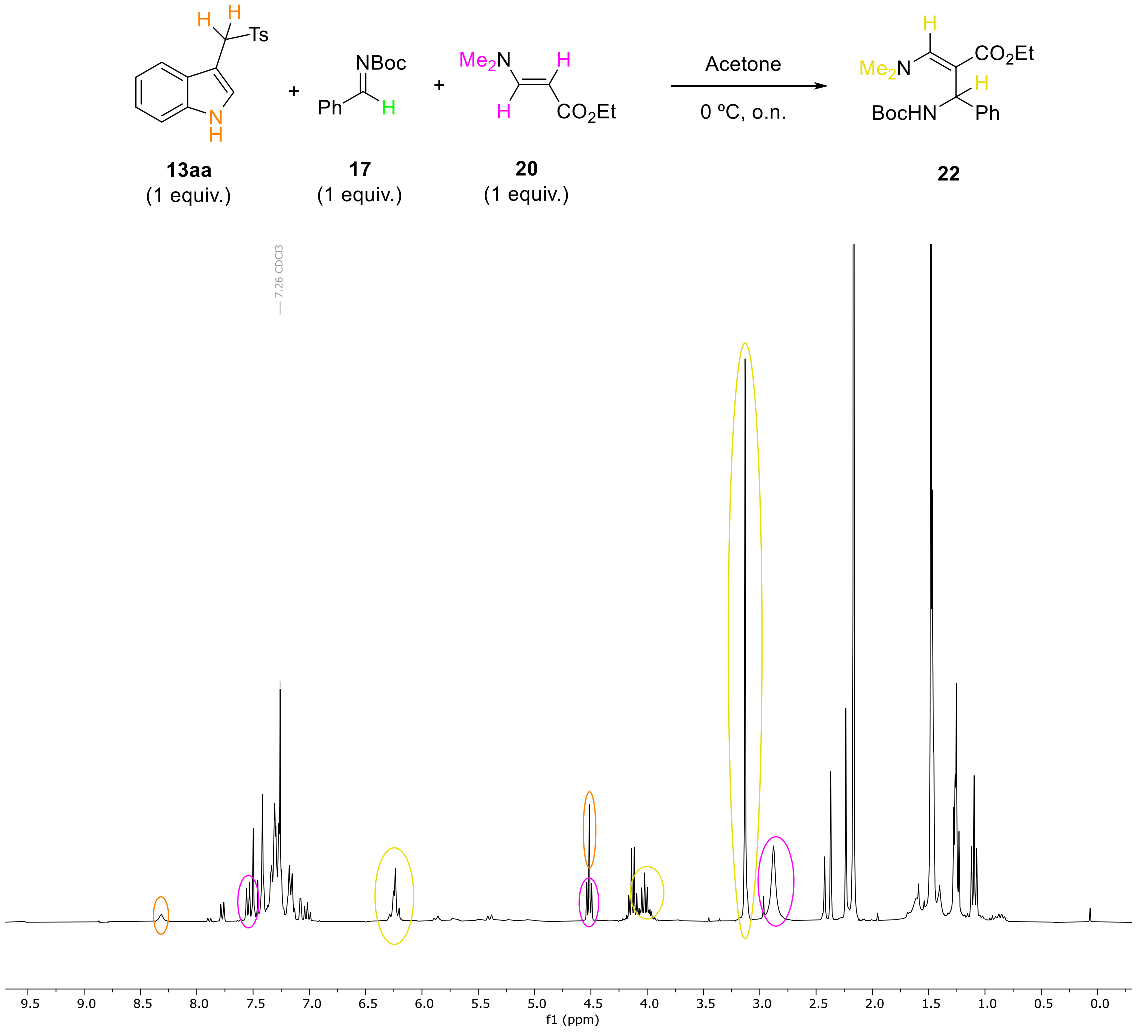

5. ^{1}H NMR temperature coefficients (ΔσHN/ΔT) for compounds 8a and 13aa

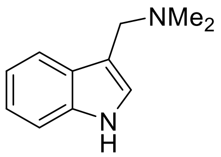

8a

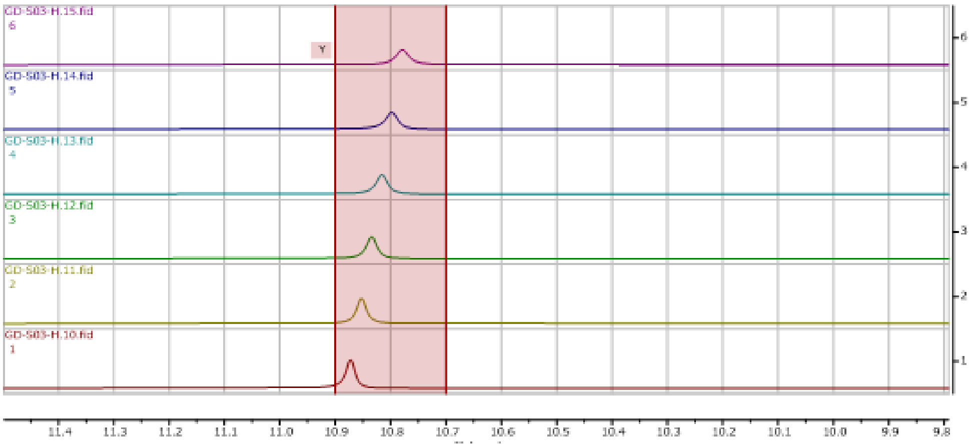

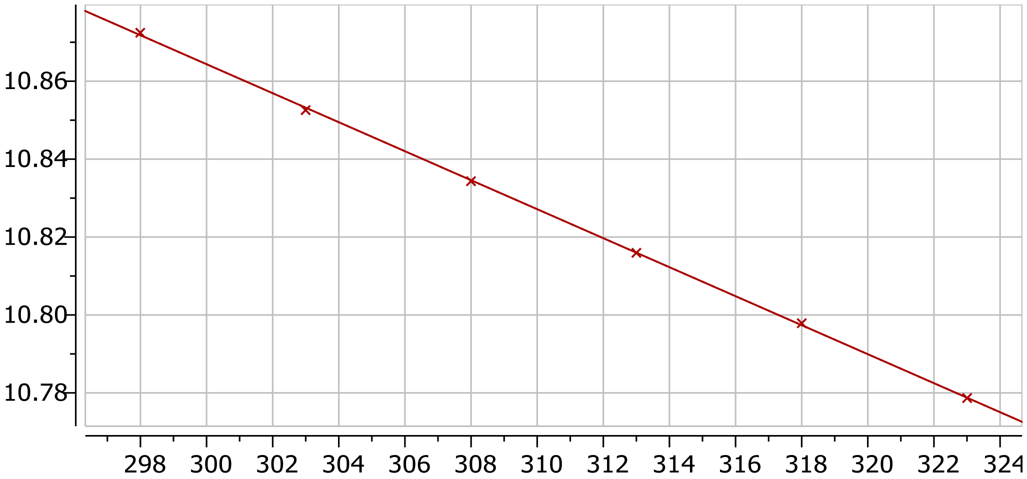

Equation of correlation straight: Y = 11.98-0.003721*X

Figure SI-1. Variation of ^{1}H-NMR NH peak of gramine **8a** alone in DMSO: $\Delta\delta/\Delta T(H_N)$ = –3.7

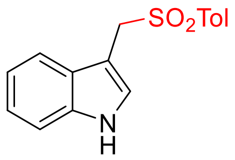

13aa

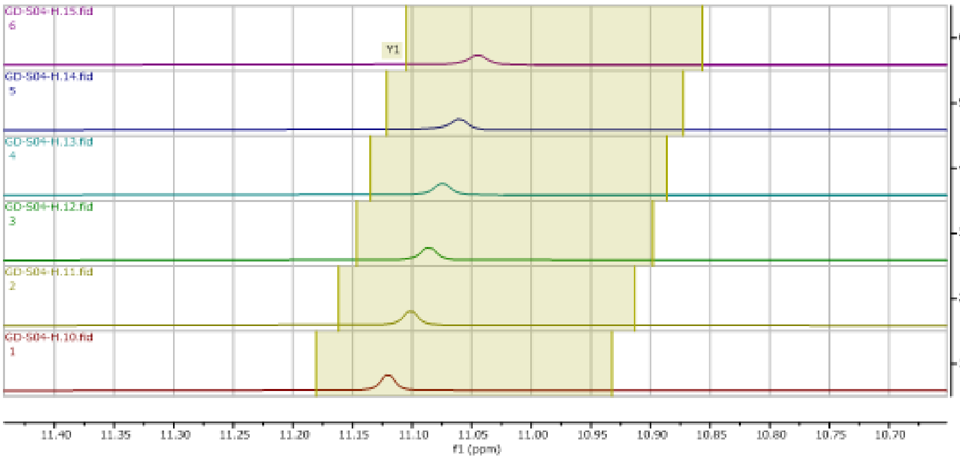

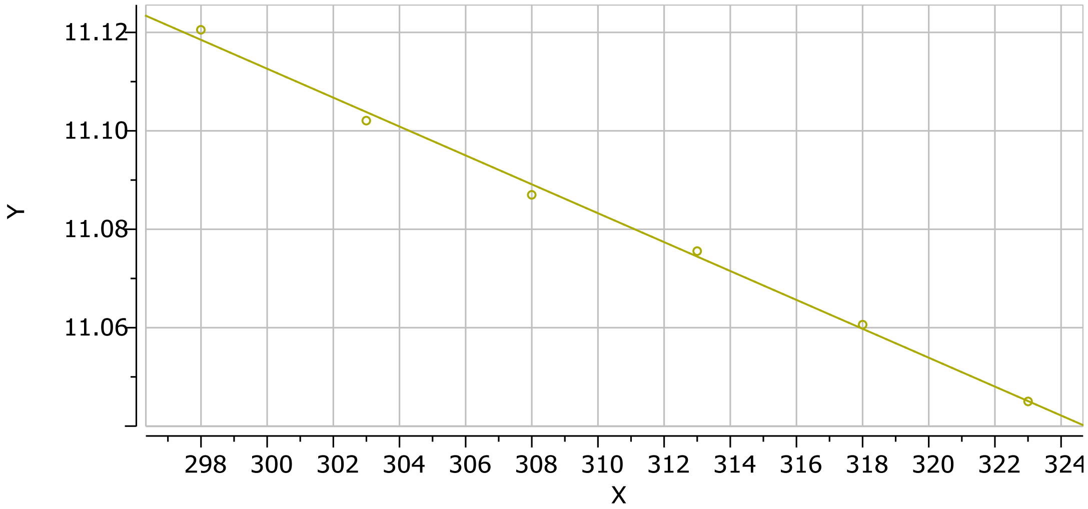

Equation of correlation straight: Y = 11.99-0.002936*X

Figure SI-2. Variation of ^{1}H-NMR NH peak of sulfone **13aa** alone in DMSO: $\Delta\delta/\Delta T(H_N)$ = –2.9

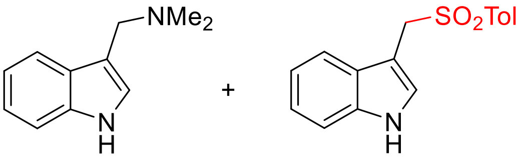

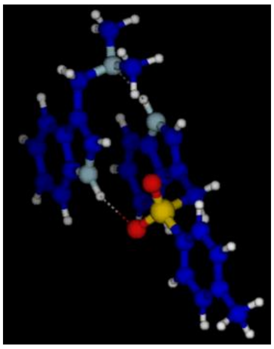

Equation of correlation straight for

Gramine **8a**: Y = 12.0901-0.00289296*X

Sulfone **13aa**: Y = 12.6748-0.00243207*X

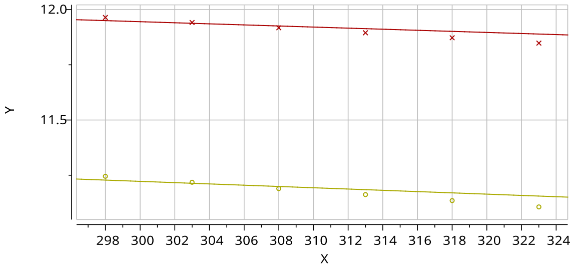

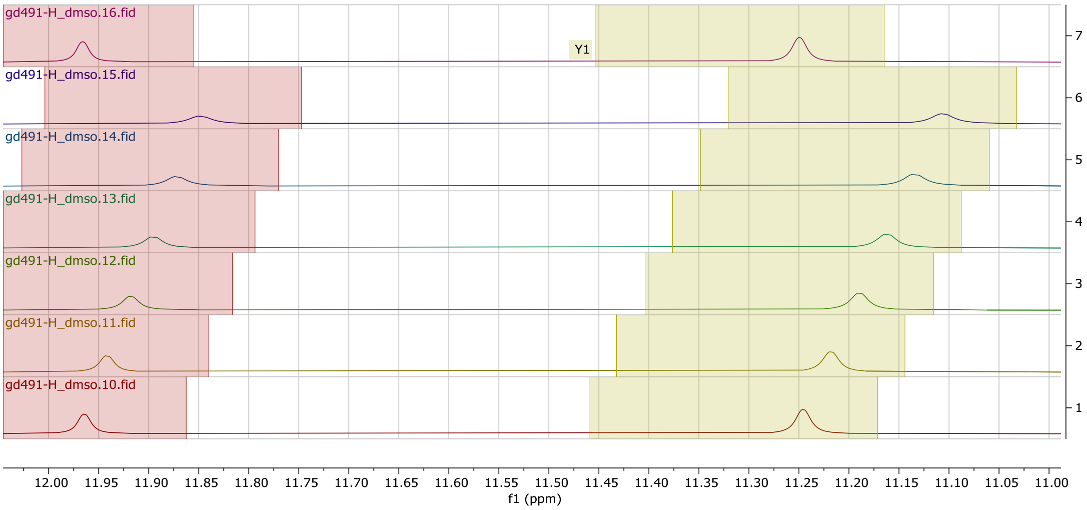

Y MaxPeakPos(11.46,11.03)

	X(I)	Y(X)	Y'(X)	Y1(X)	Y1'(X)
Modelo	ARR_DATA(I)	MaxPeakPos(12.119202,11.747302)	A+B*x A= 12.6748 B= -0.00243207	MaxPeakPos(11.459913,11.032227)	A+B*x A= 12.0901 B= -0.00289296
1	298	11.9643	11.95	11.2458	11.228
2	303	11.9419	11.9379	11.2183	11.2135
3	308	11.9181	11.9257	11.1897	11.199
4	313	11.8955	11.9135	11.1623	11.1846
5	318	11.8724	11.9014	11.1345	11.1701
6	323	11.8486	11.8892	11.1064	11.1557

Figure SI-3. Variation of ^{1}H-NMR NH peaks after admixing gramine **8a** and sulfone **13aa** (one equivalent each) in DMSO: $\Delta\delta/\Delta T(H_N) = -2.4$ (sulfone) and -2.9 (gramine).

6. ^{1}H, ^{13}C and ^{19}F NMR spectra.

^{1}H NMR (300 MHz, $CDCl_3$) of **1a**

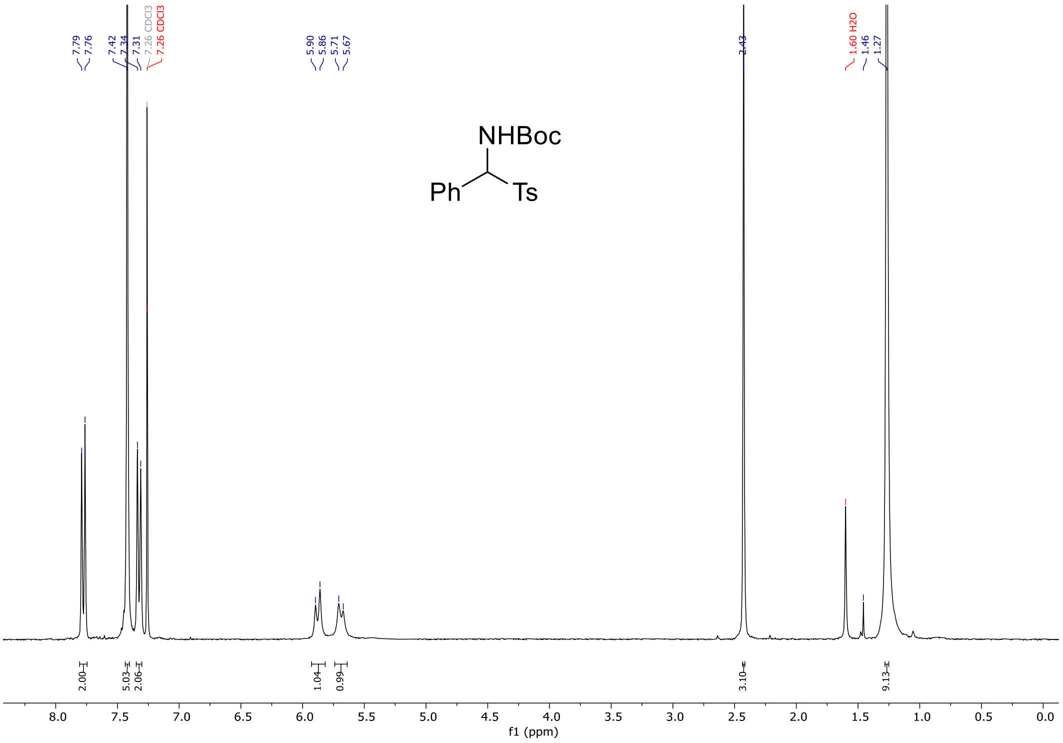

^{1}H NMR (300 MHz, $CDCl_3$) of **1a'**

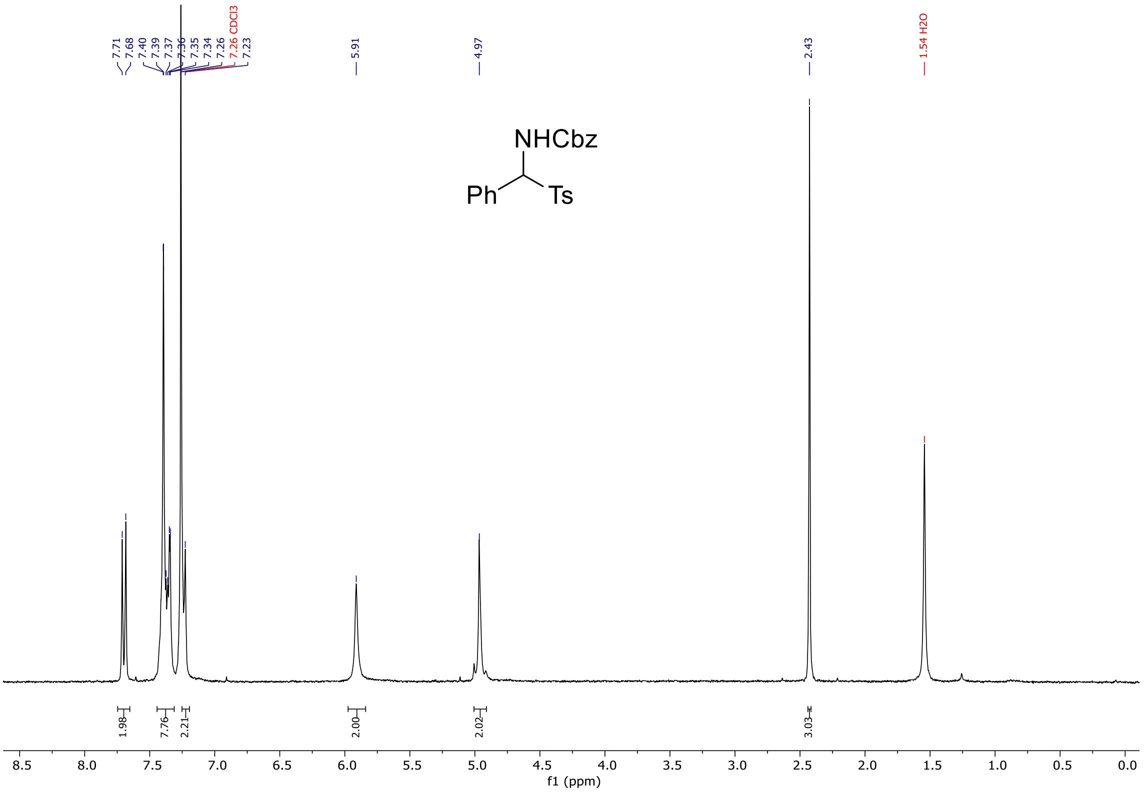

^{1}H NMR (300 MHz, $CDCl_3$) of **1b**

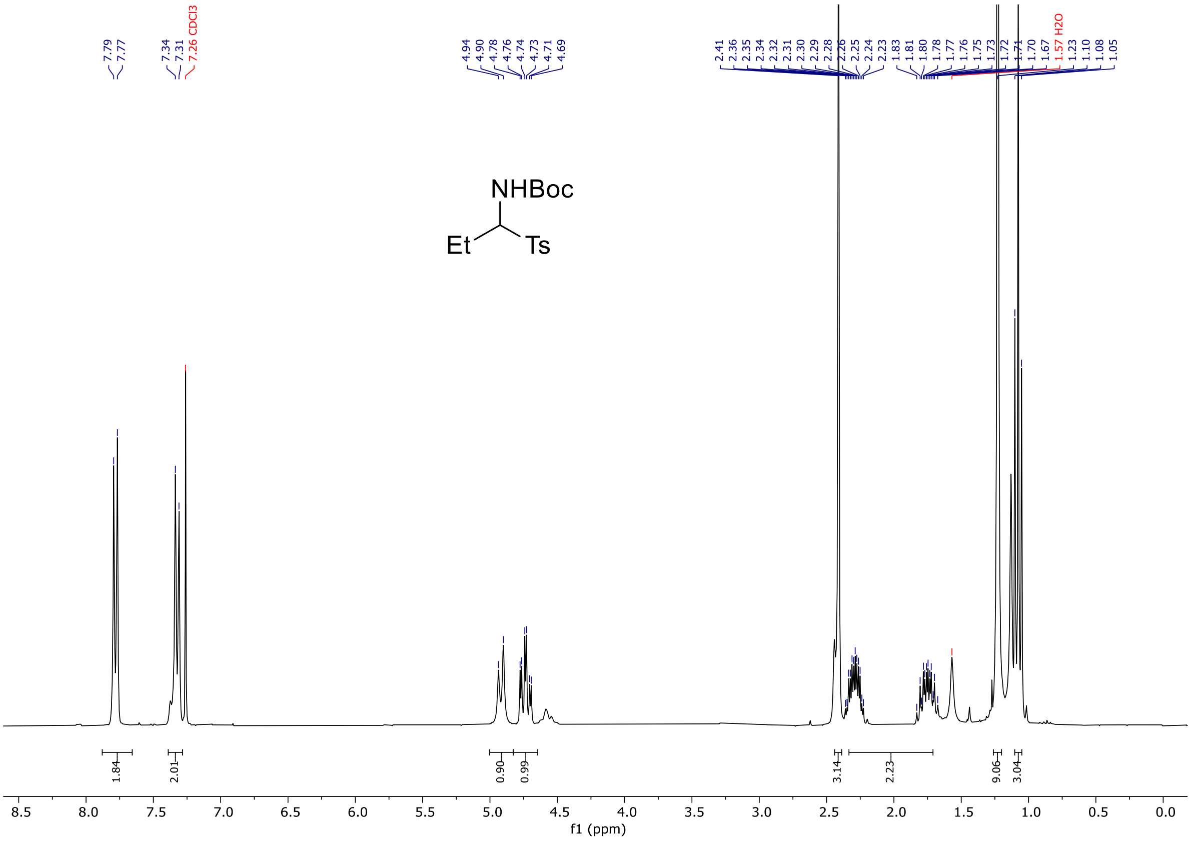

^{1}H NMR (300 MHz, $CDCl_3$) of **1c**

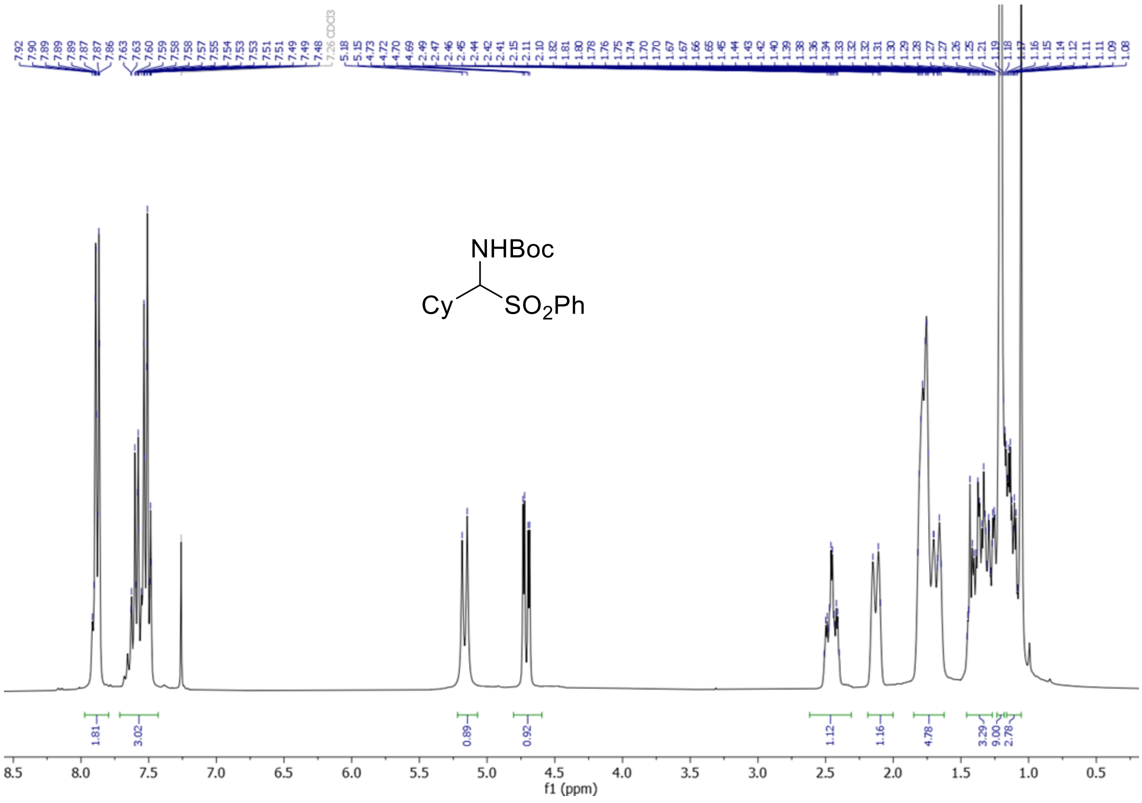

^{1}H NMR (300 MHz, $CDCl_3$) of **1d**

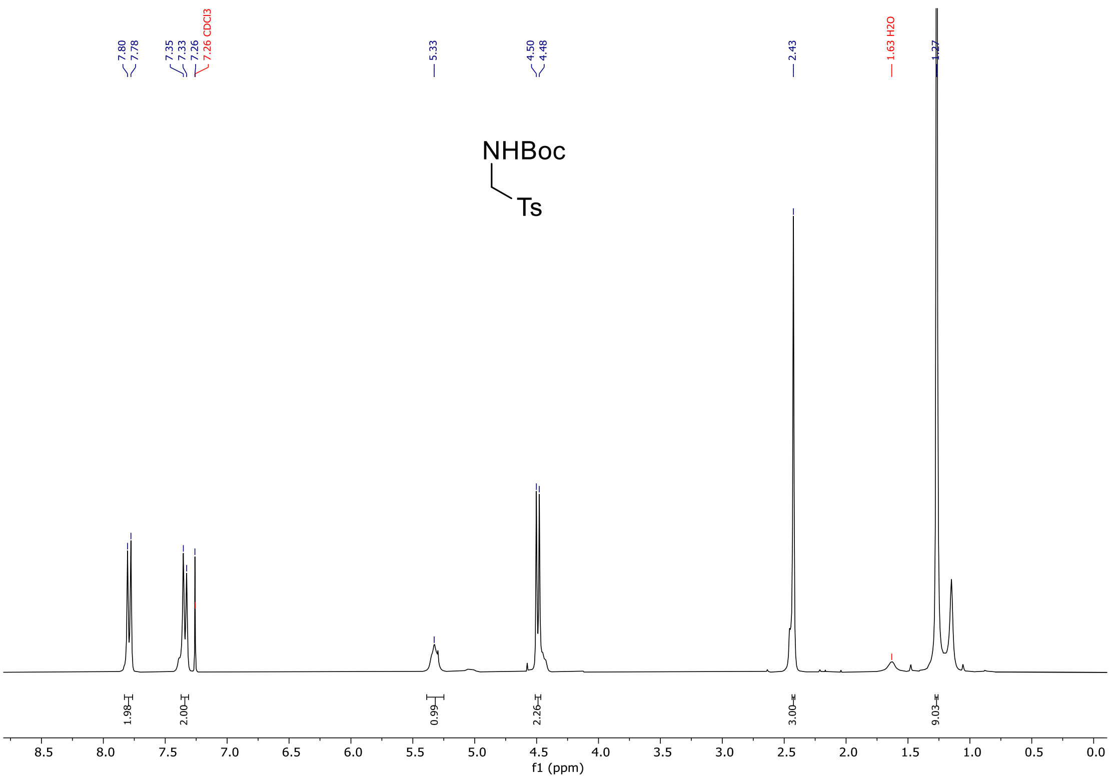

^{1}H NMR (300 MHz, $CDCl_3$) of **1e**

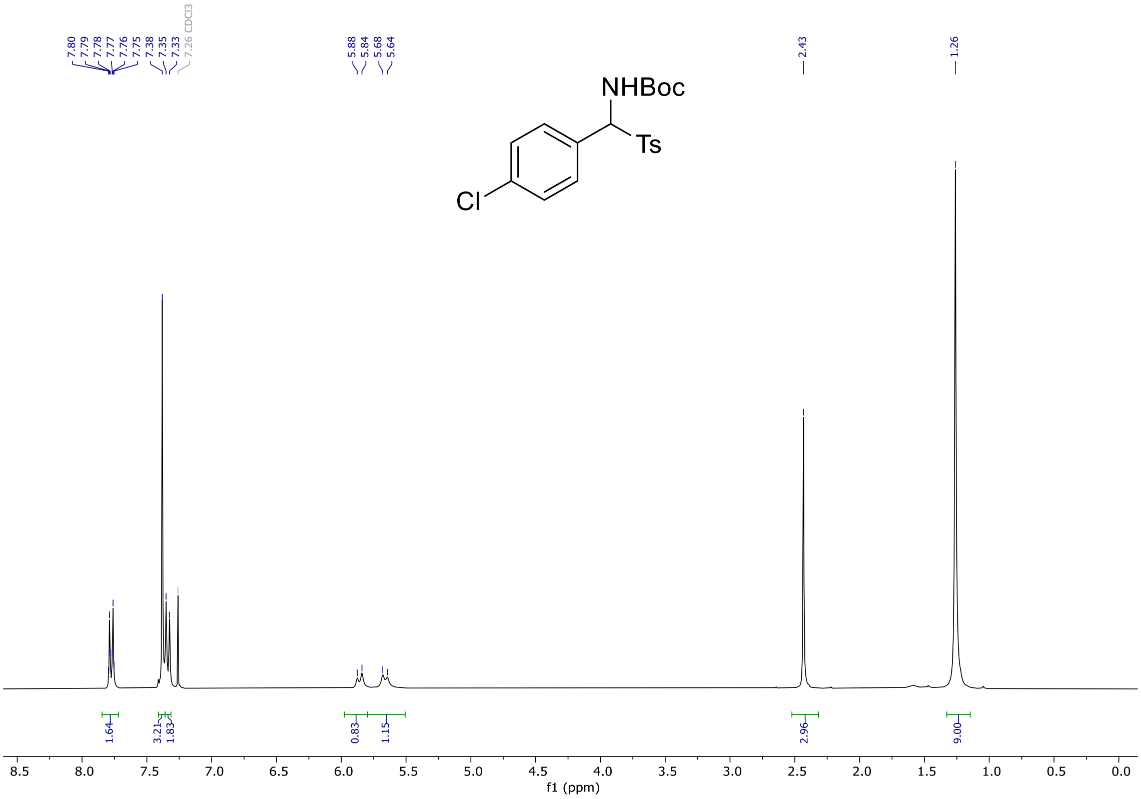

^{1}H NMR (300 MHz, $CDCl_3$) of **1f**

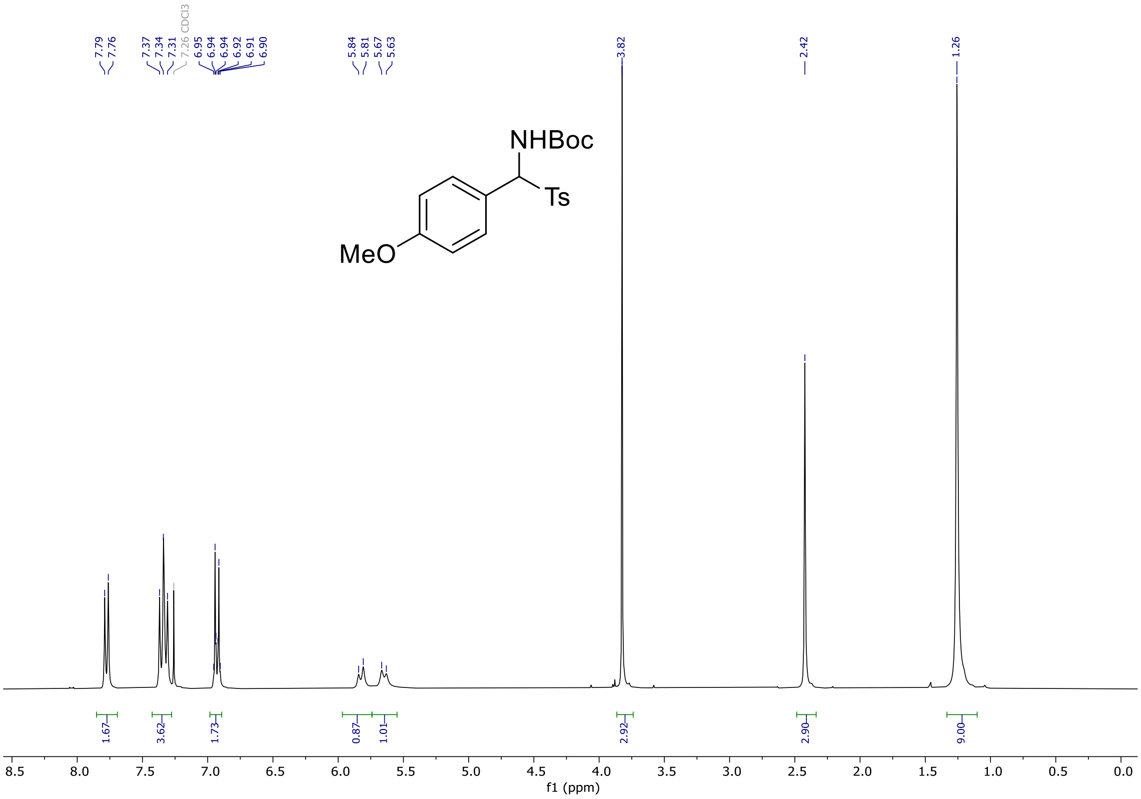

^{1}H NMR (300 MHz, $CDCl_3$) of **8n**

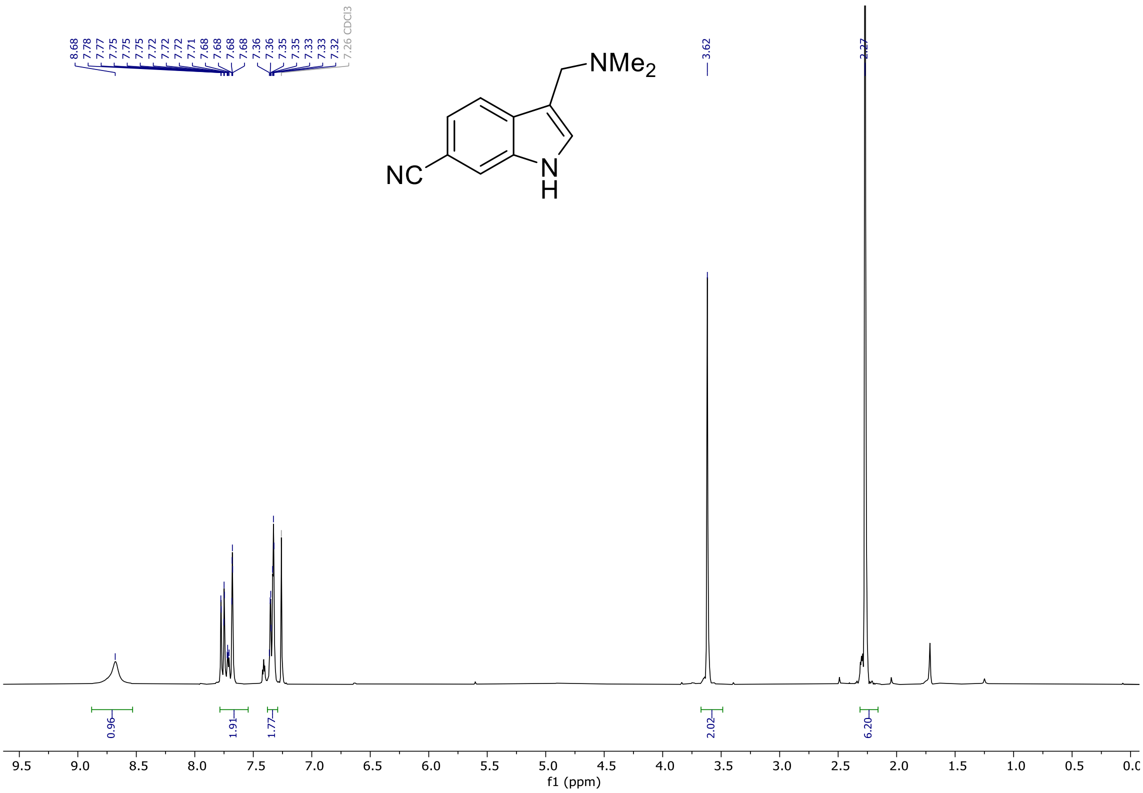

^{1}H NMR (300 MHz, $CDCl_3$) of **8t**

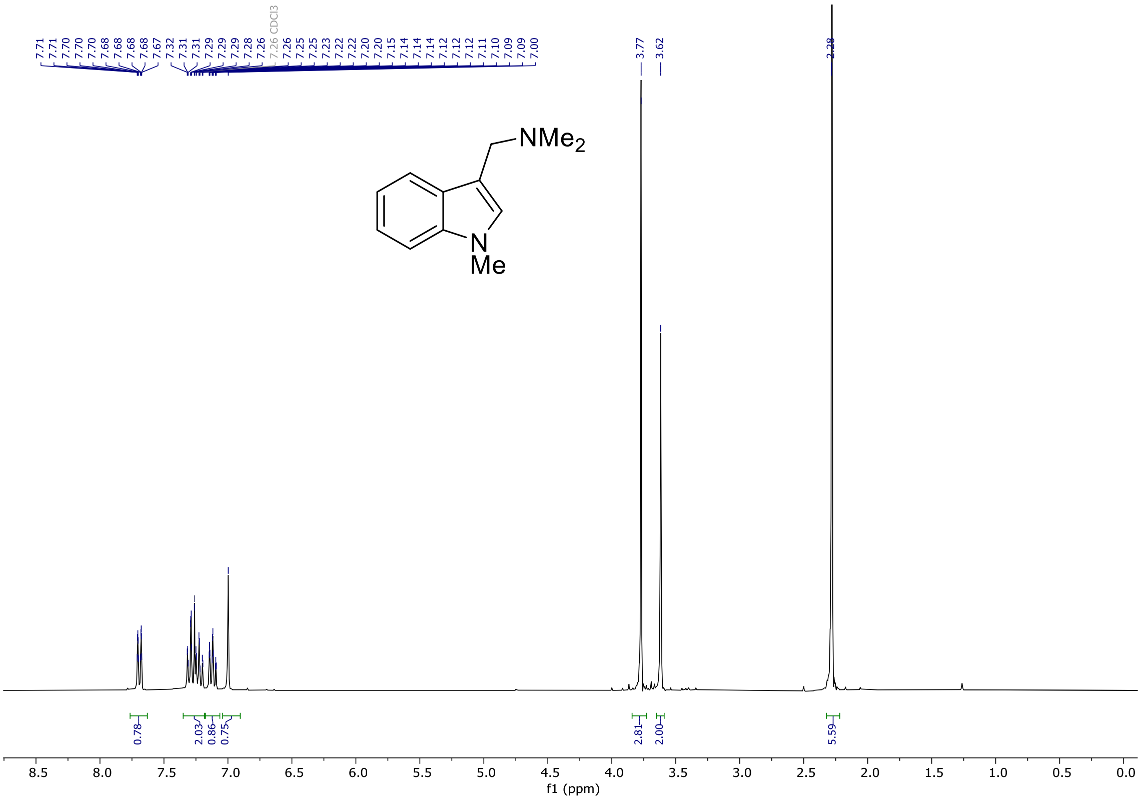

^{1}H NMR (300 MHz, Methanol-d_4) of **15**

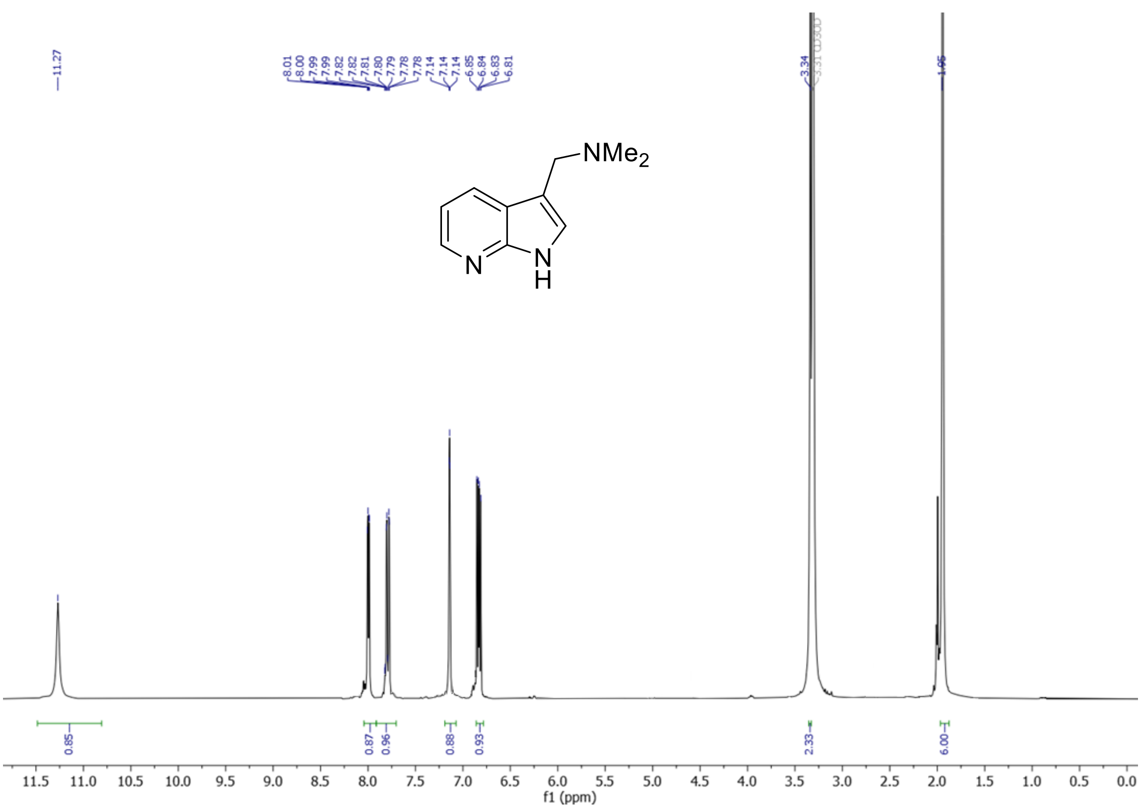

$^{13}C\{^{1}H\}$ NMR (75 MHz, DMSO-d_6) of **15**

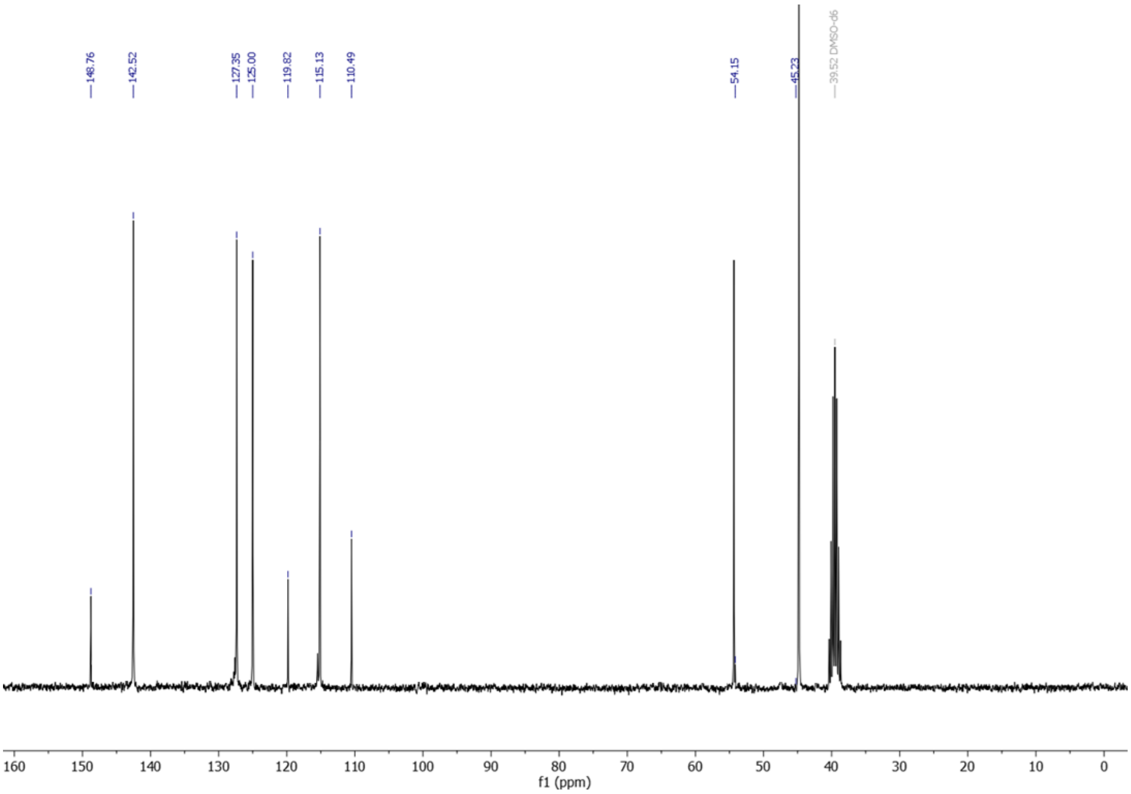

^{1}H NMR (300 MHz, $CDCl_3$) of **13aa**

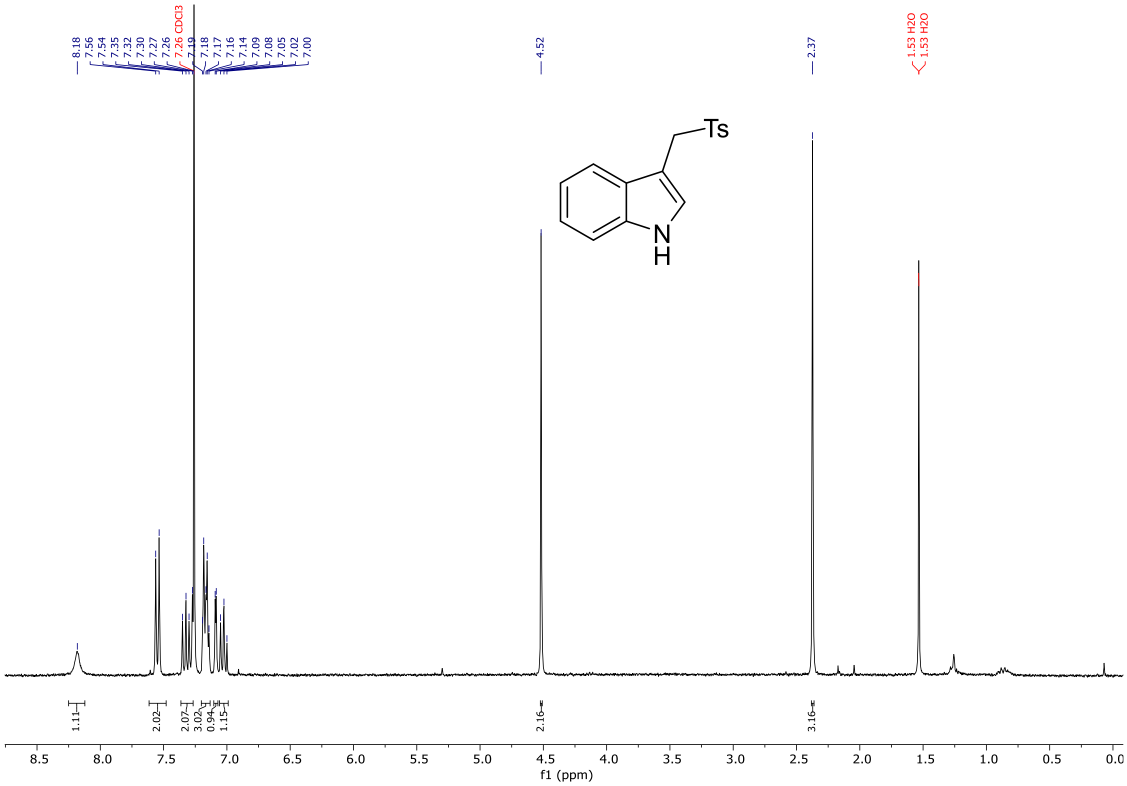

^{1}H NMR (300 MHz, $CDCl_3$) of **13ab**

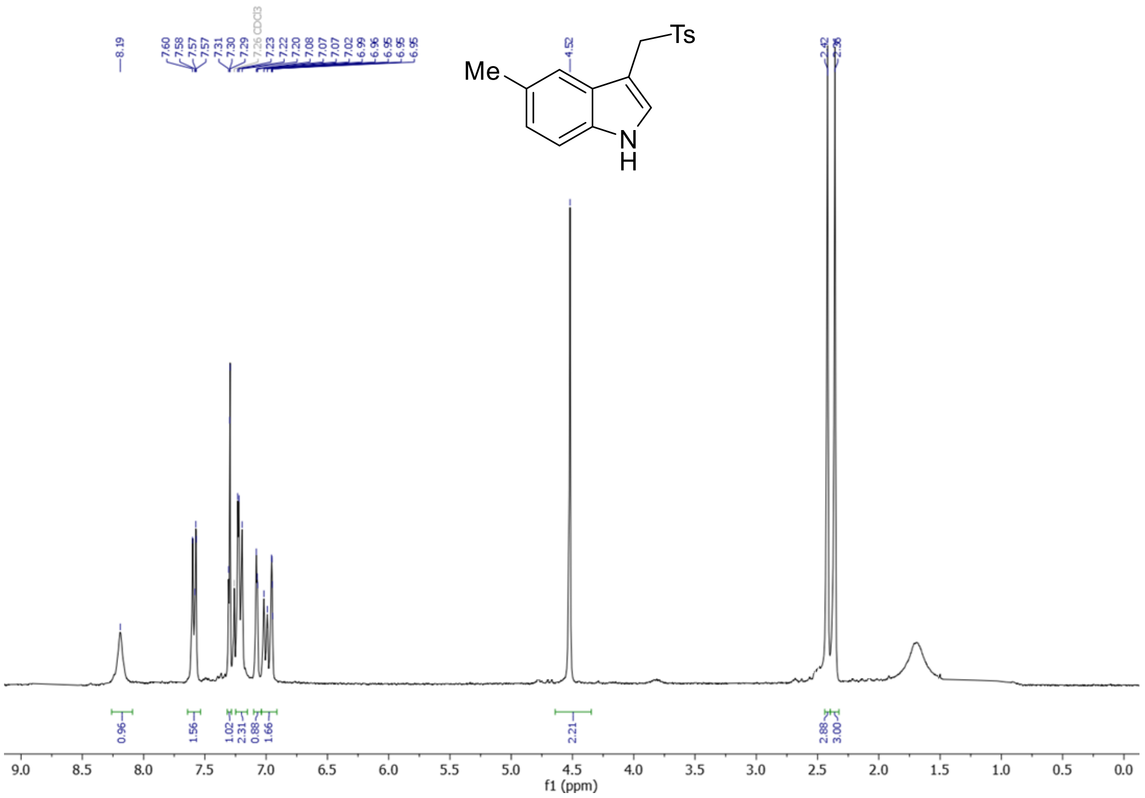

^{1}H NMR (300 MHz, $CDCl_3$) of **13ac**

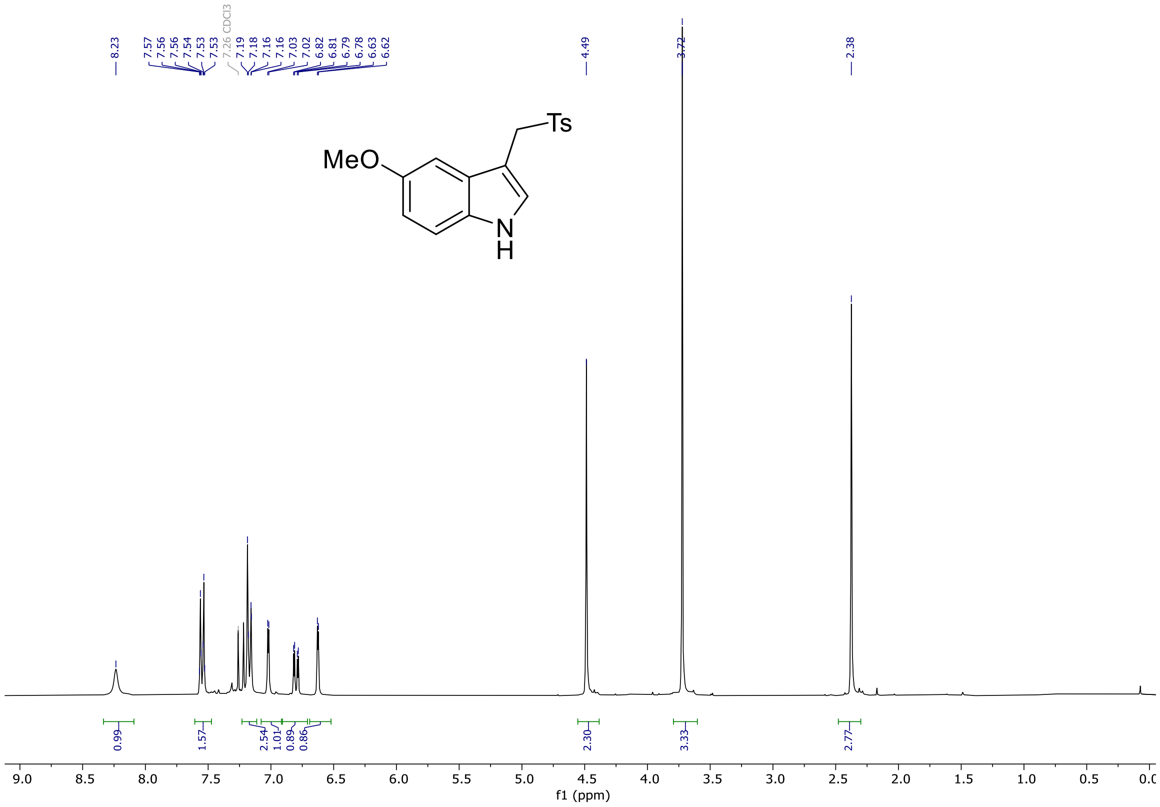

^{1}H NMR (300 MHz, $CDCl_3$) of **13ad**

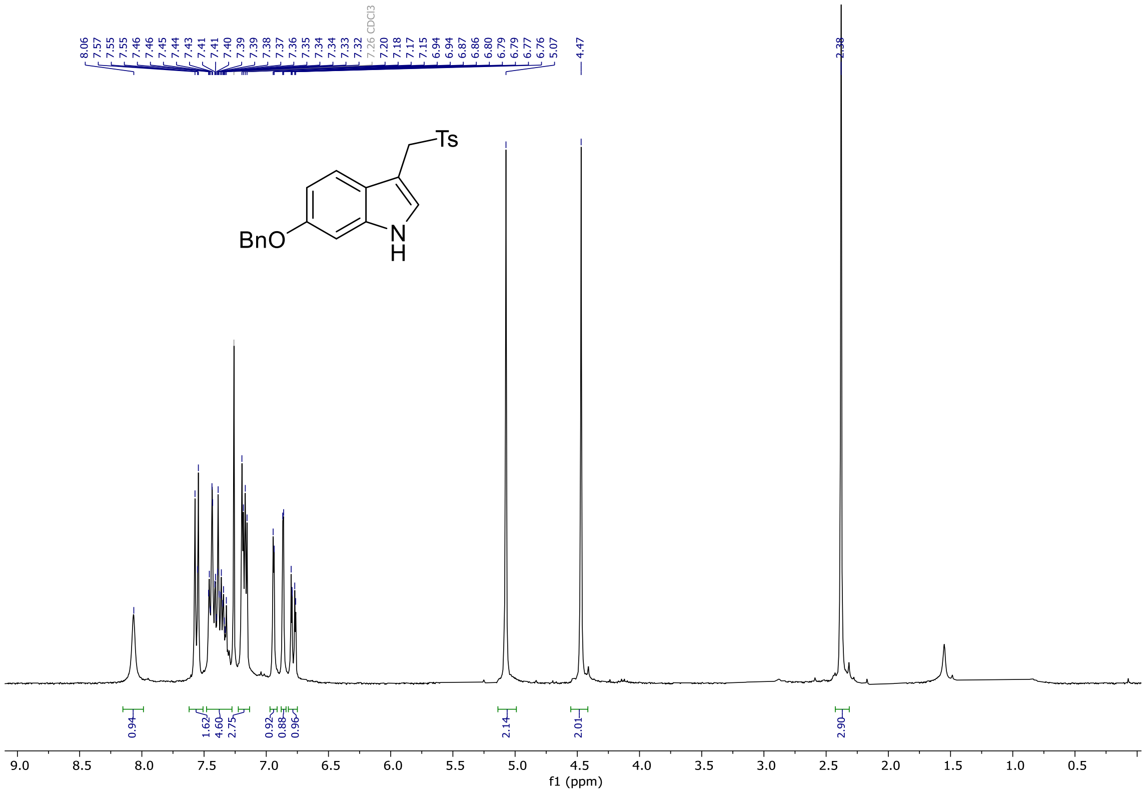

^{1}H NMR (300 MHz, $CDCl_3$) of **13ae**

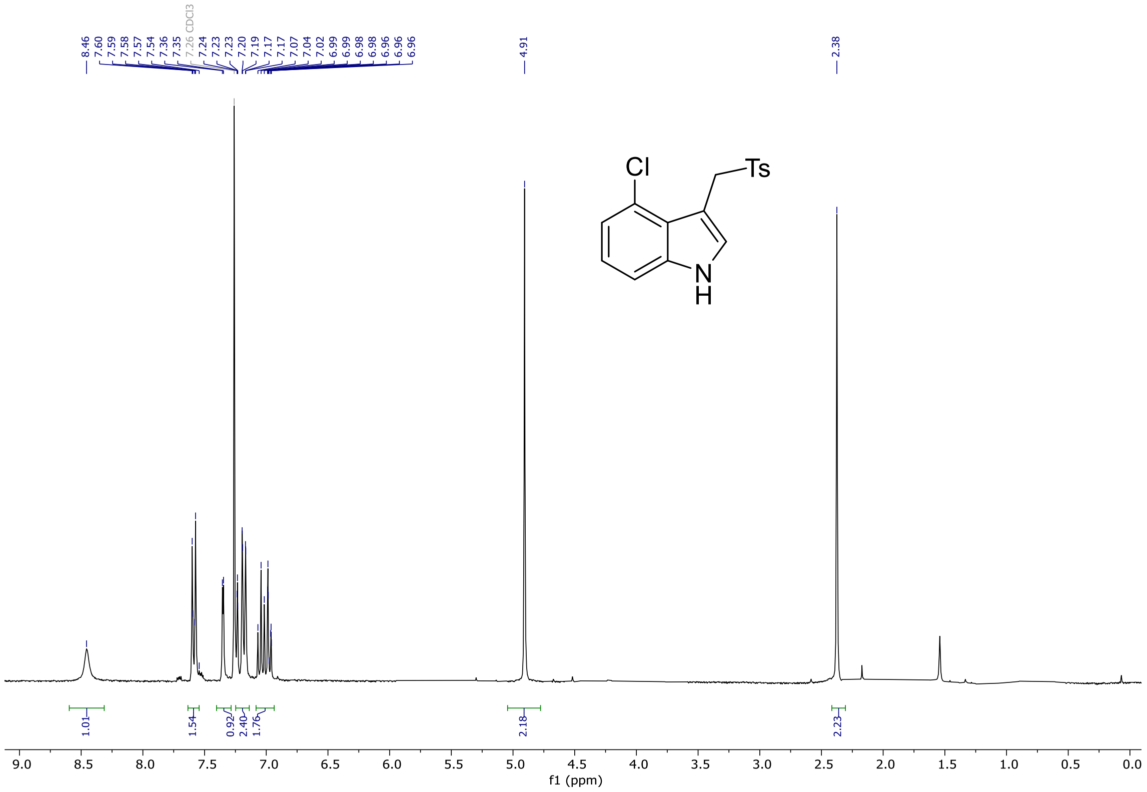

^{1}H NMR (300 MHz, DMSO-d_6) of **13af**

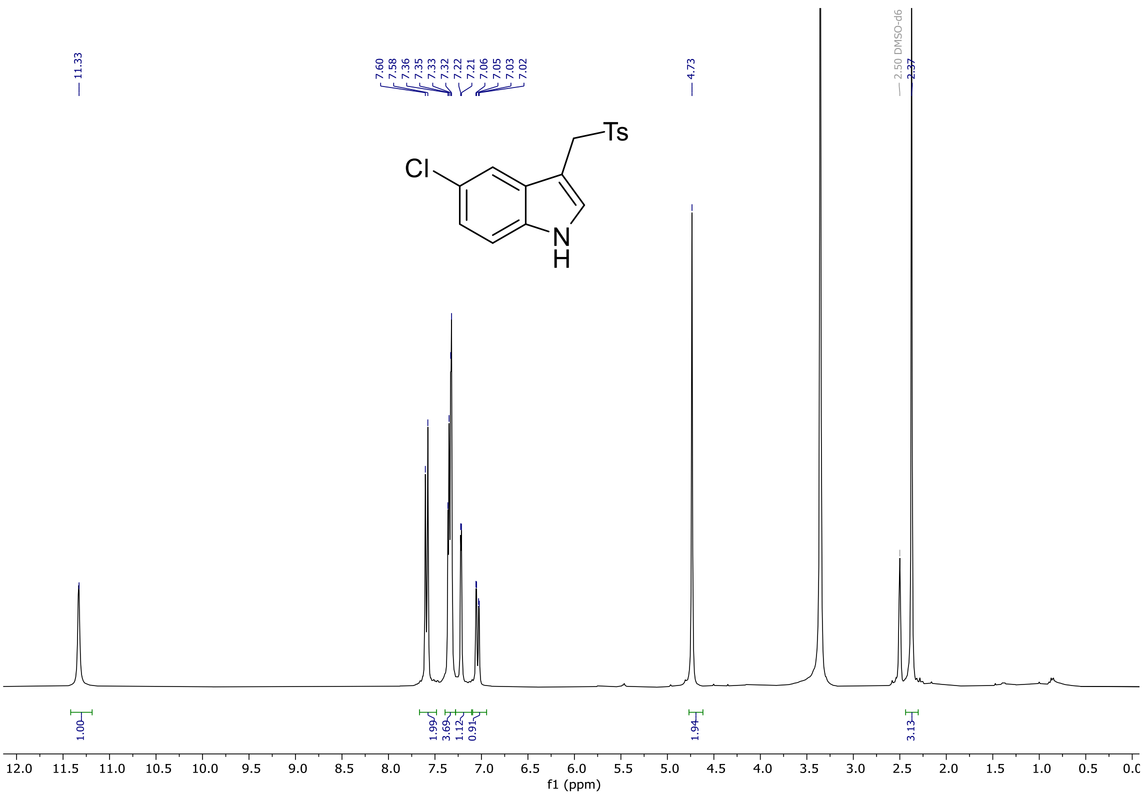

$^{13}C\{^{1}H\}$ NMR (75 MHz, DMSO-d_6) of **13af**

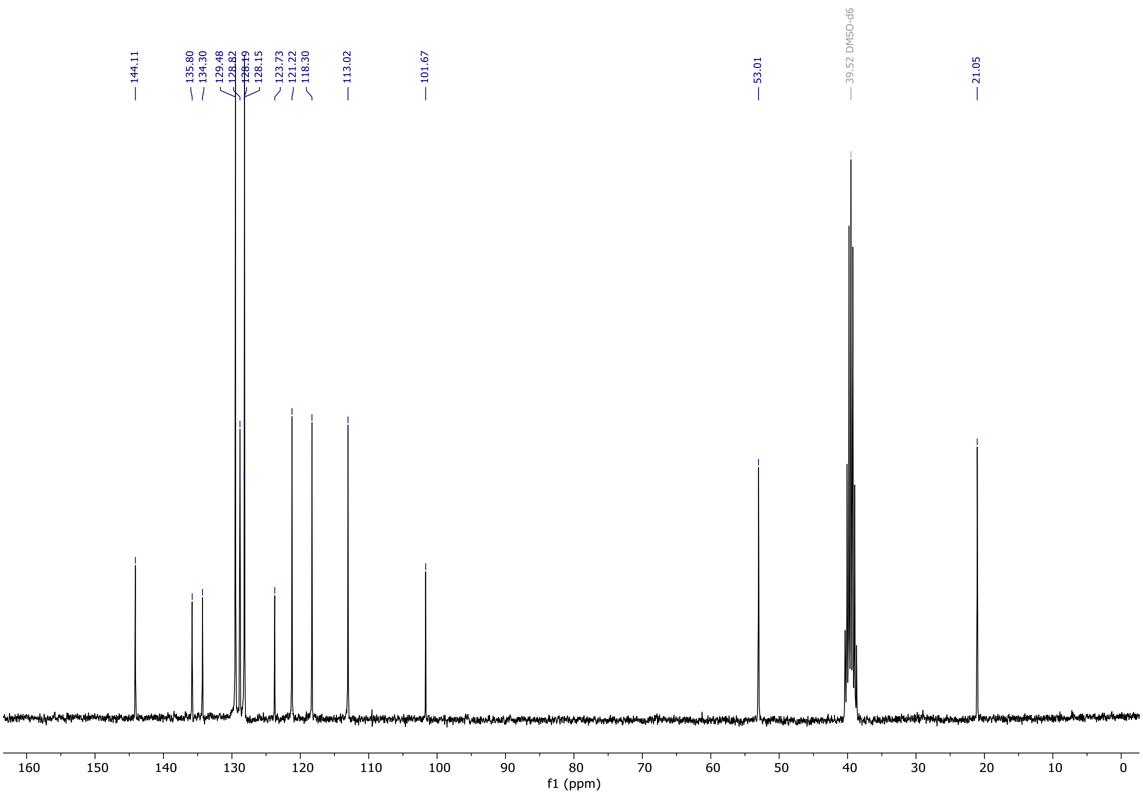

^{1}H NMR (300 MHz, $CDCl_3$) of **13ag**

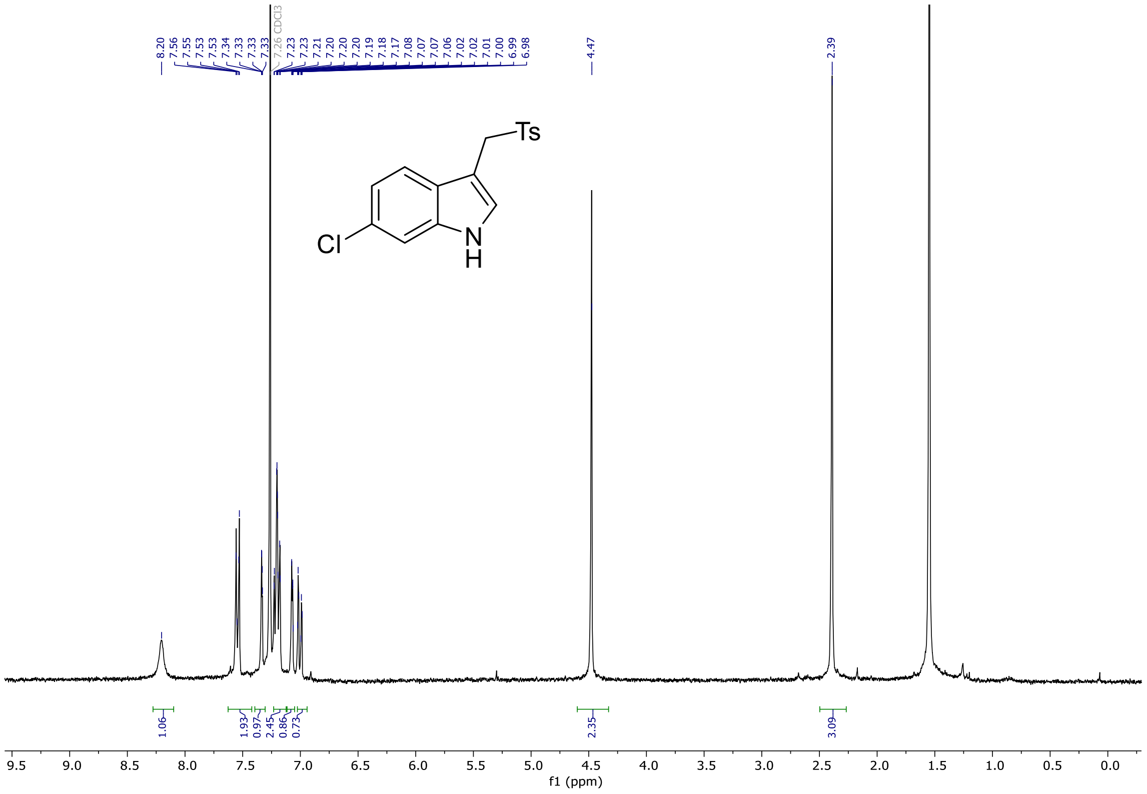

^{1}H NMR (300 MHz, $CDCl_3$) of **13ah**

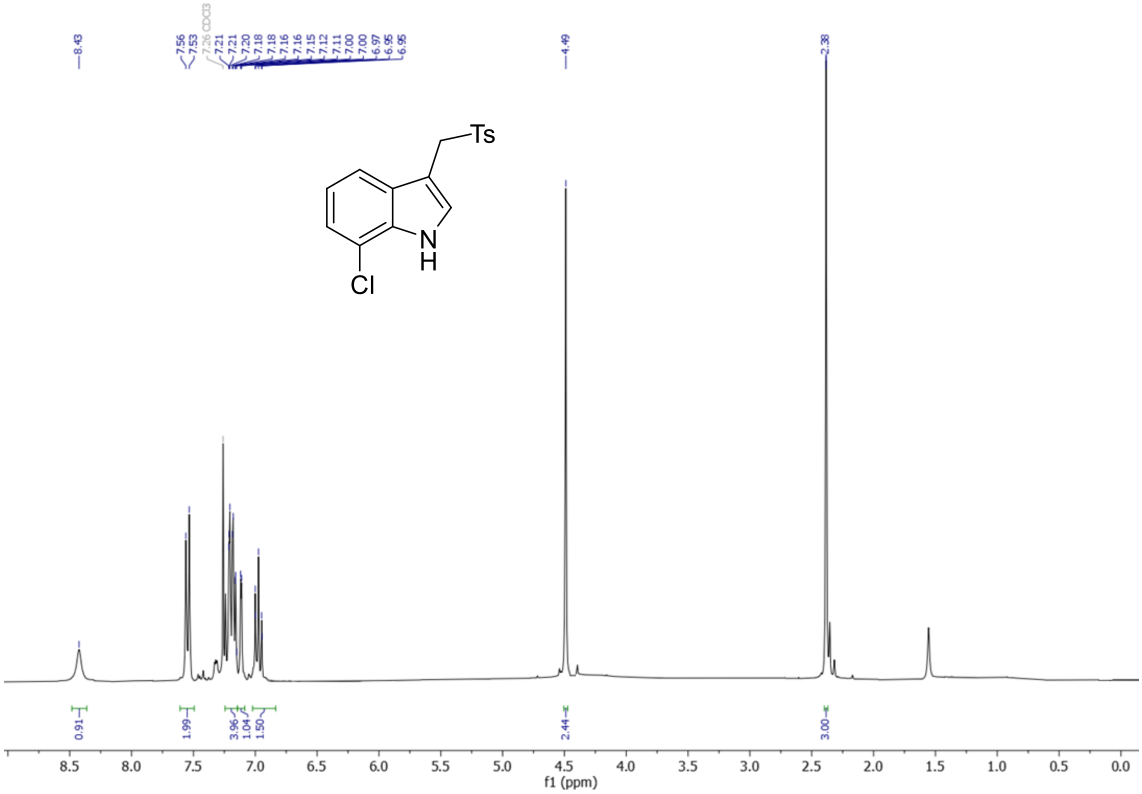

^{13}C{^{1}H} NMR (75 MHz, $CDCl_3$) of **13ah**

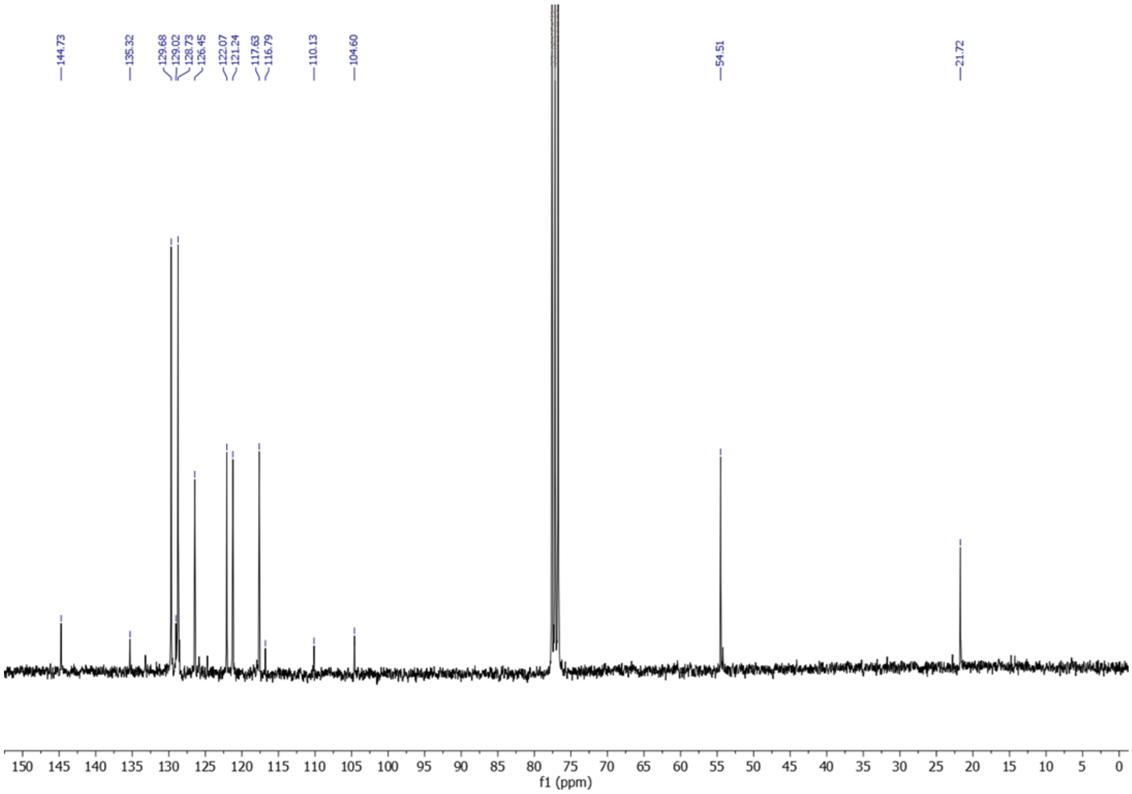

^{1}H NMR (300 MHz, Methanol-d_4) of **13ai**

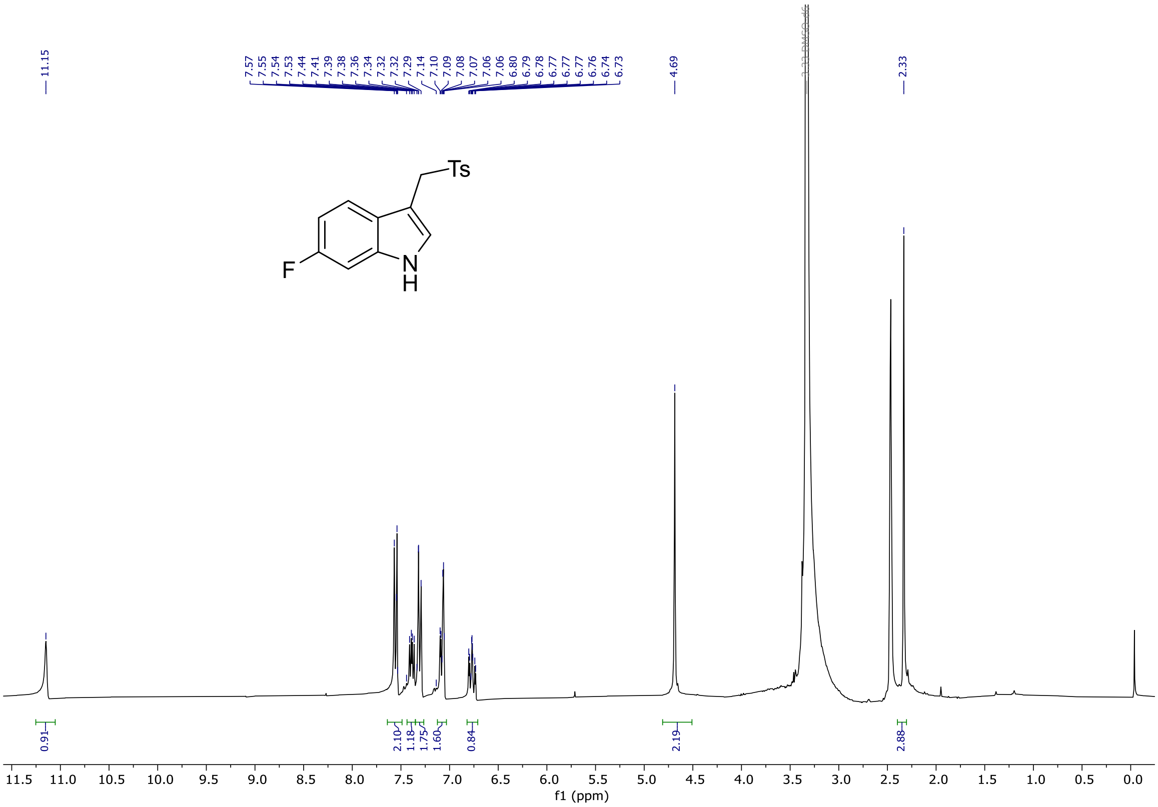

^{19}F NMR (471 MHz, DMSO-d_6) of **13ai**

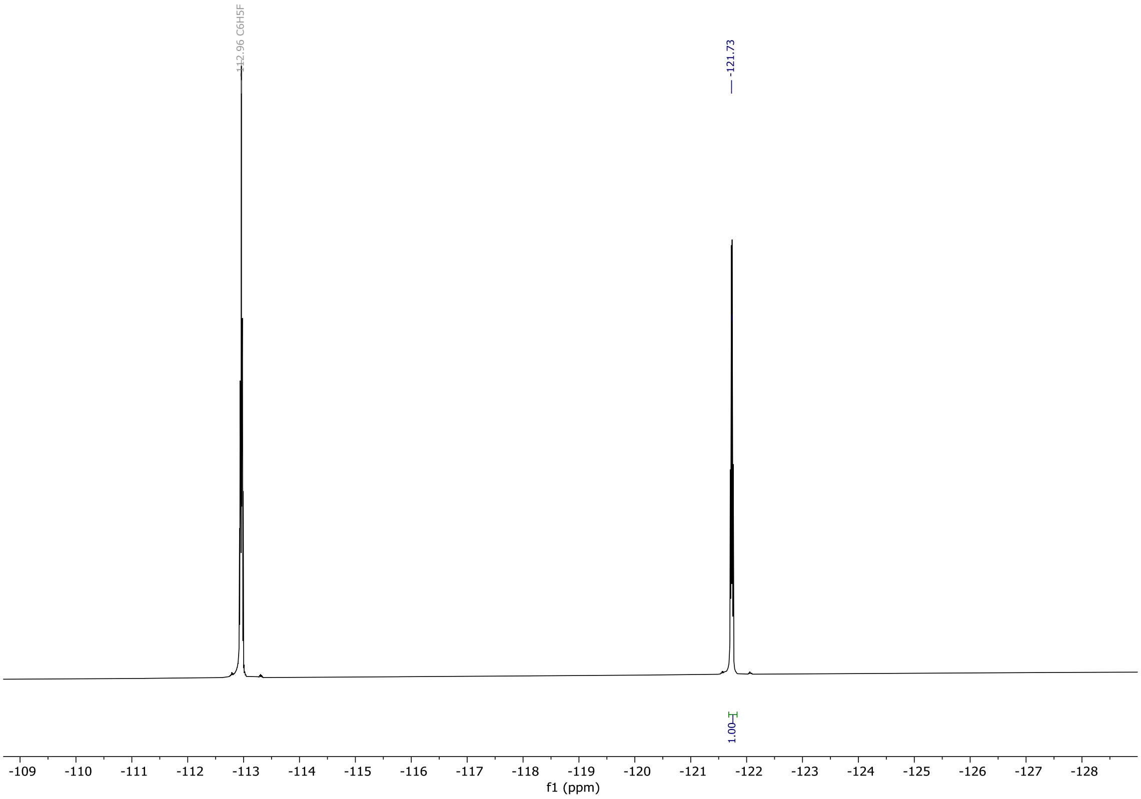

^{1}H NMR (300 MHz, DMSO-d_6) of **13aj**

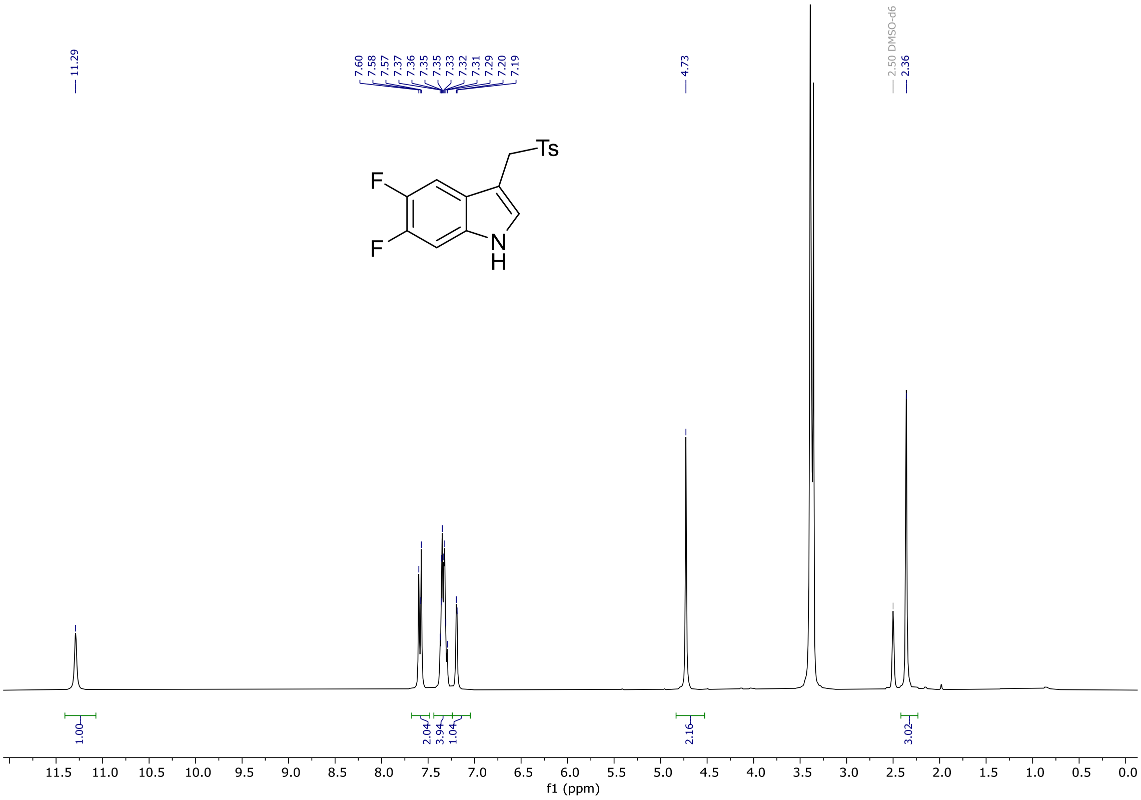

^{13}C{^{1}H} NMR (75 MHz, DMSO-d_6) of **13aj**

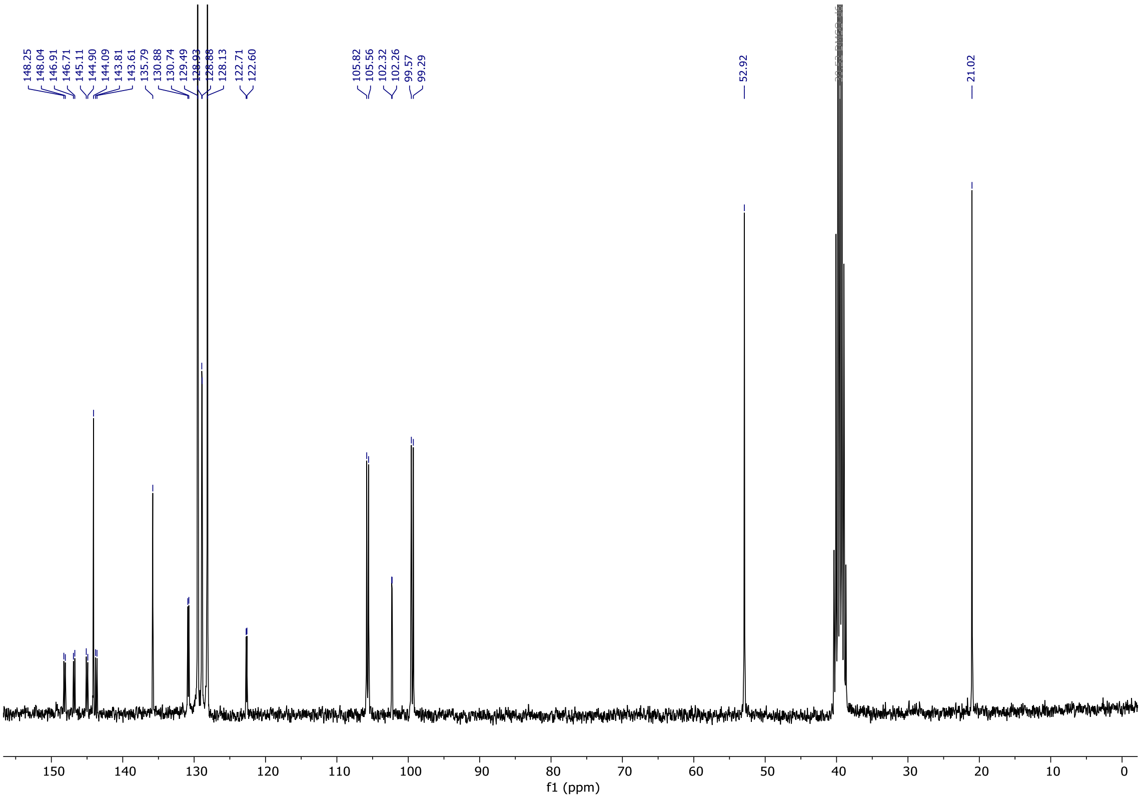

^{19}F NMR (471 MHz, DMSO-d_6) of **13aj**

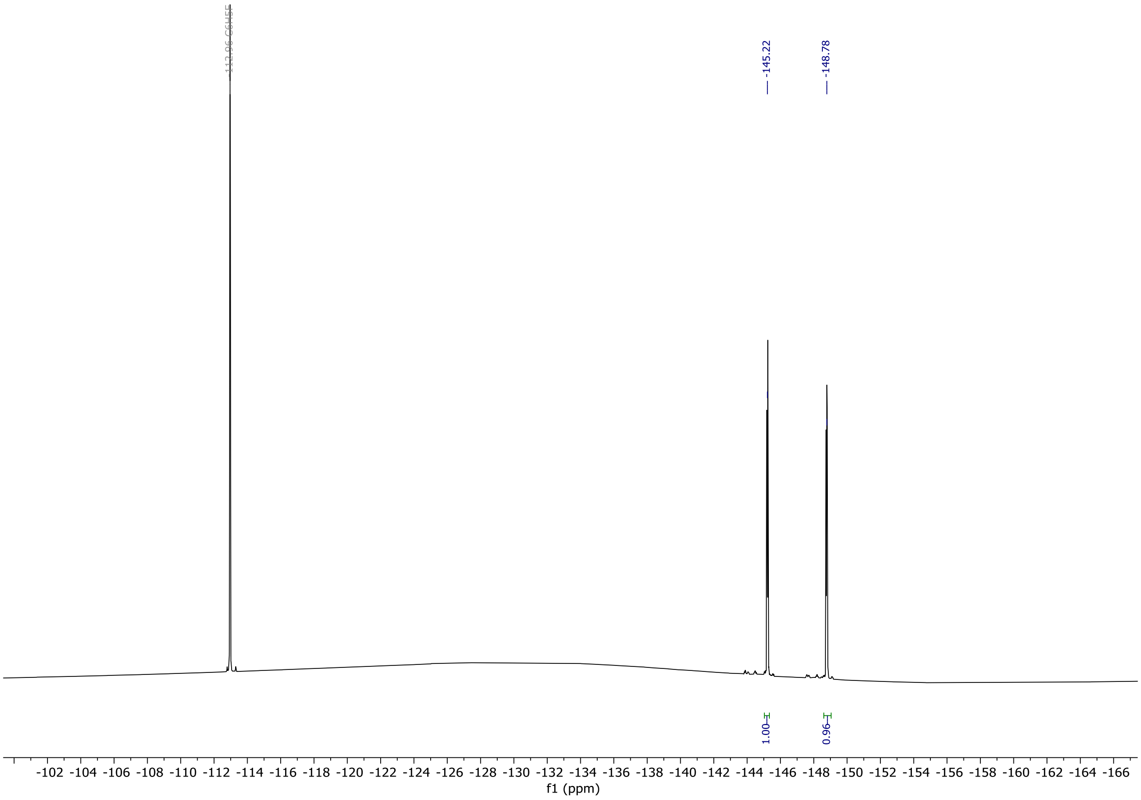

^{1}H NMR (300 MHz, Acetone-d_6) of **13ak**

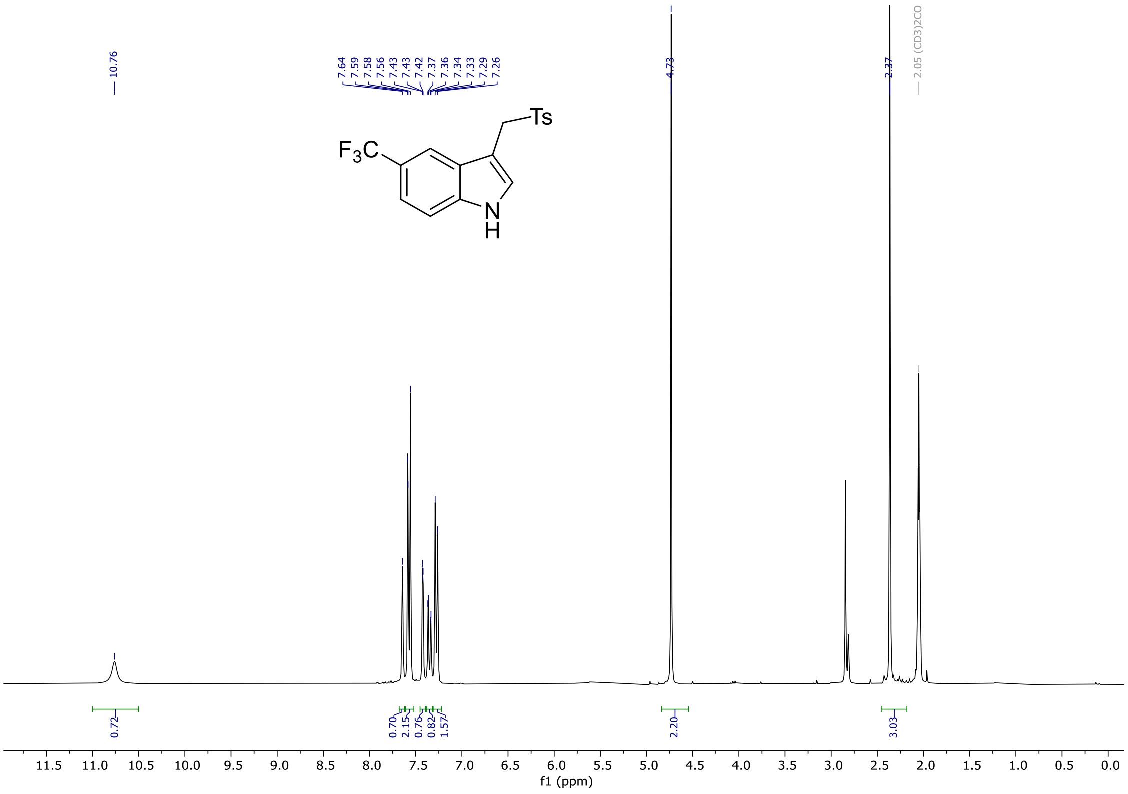

^{13}C{^{1}H} NMR (75 MHz, Acetone-d_6) of **13ak**

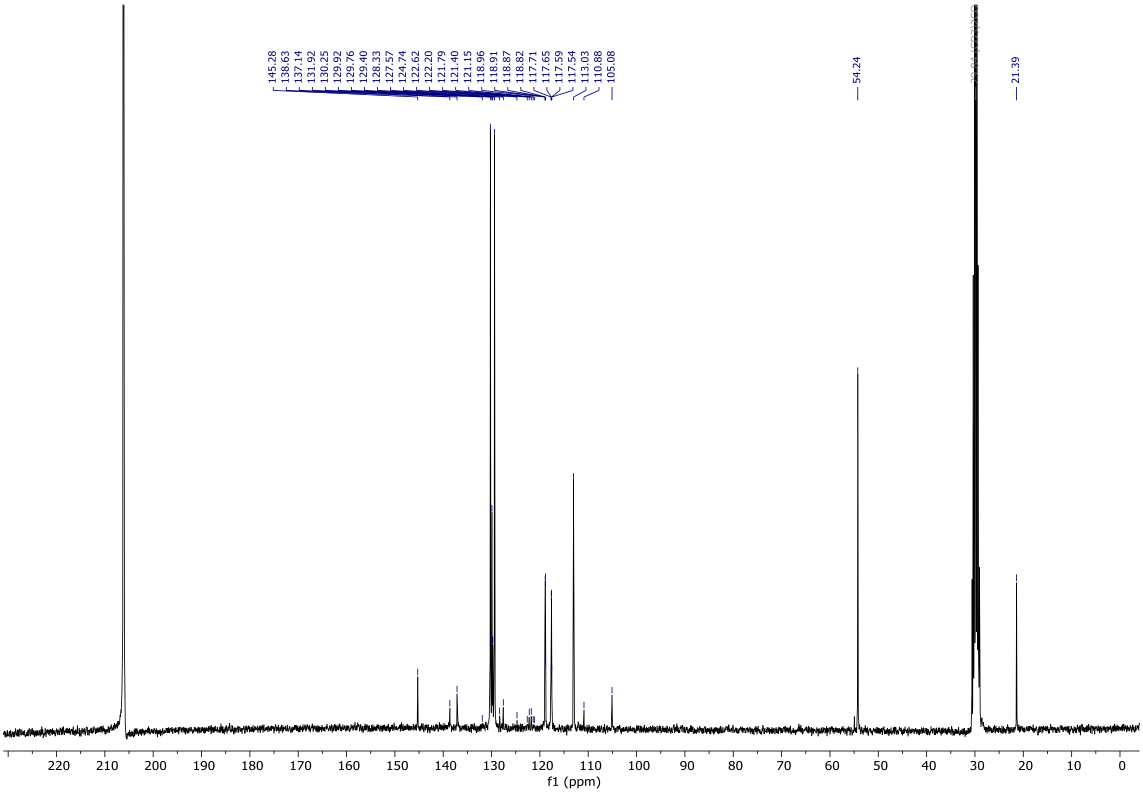

^{19}F NMR (471 MHz, Acetone-d_6) of **13ak**

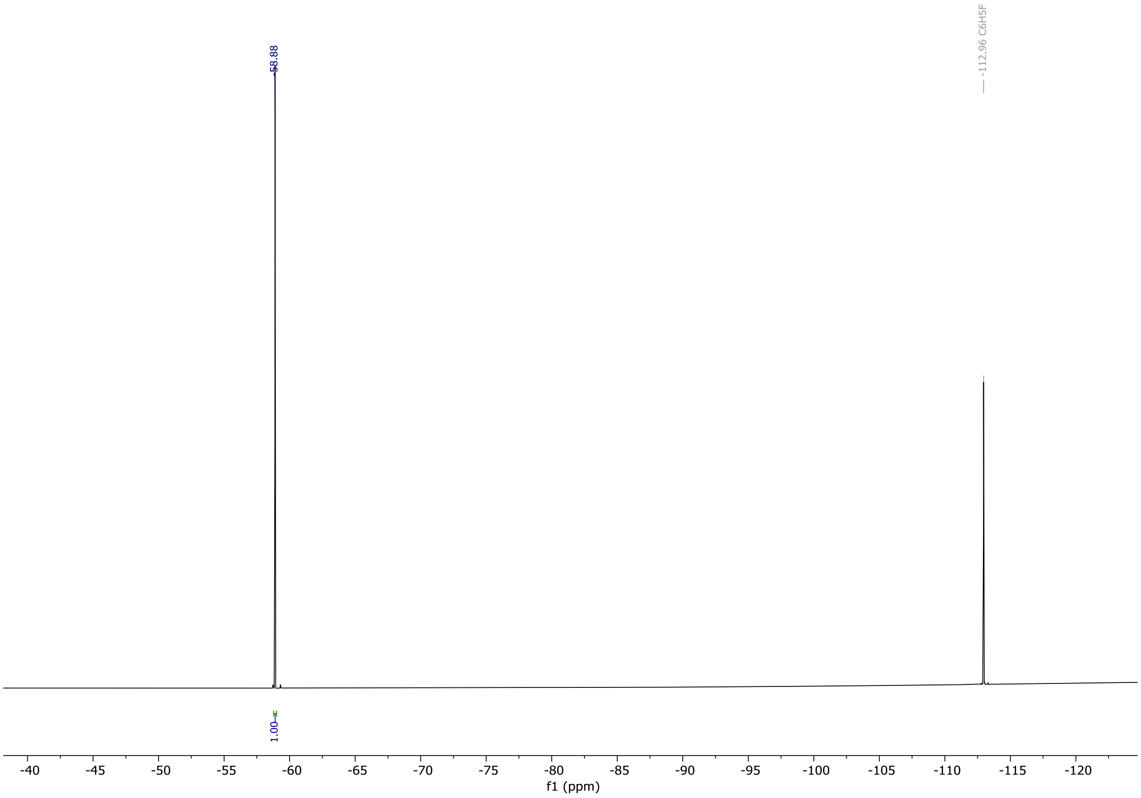

^{1}H NMR (300 MHz, $CDCl_3$) of **13al**

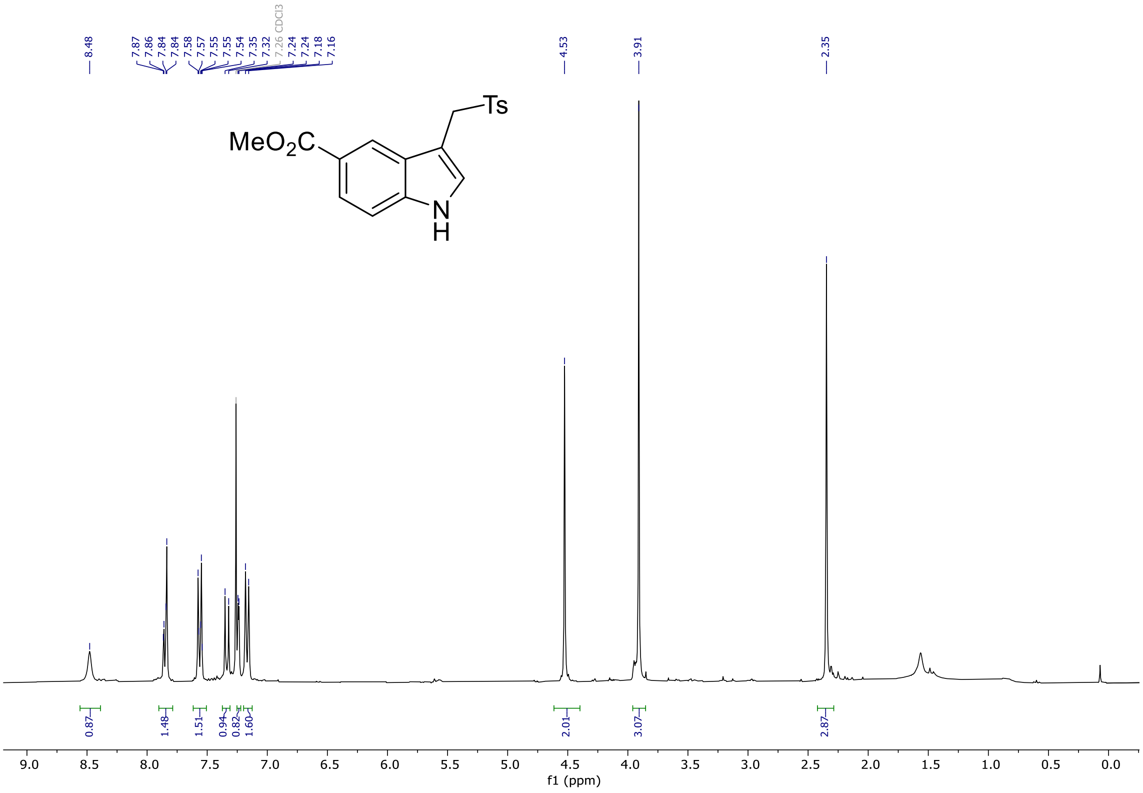

$^{13}C\{^{1}H\}$ NMR (75 MHz, $CDCl_3$) of **13al**

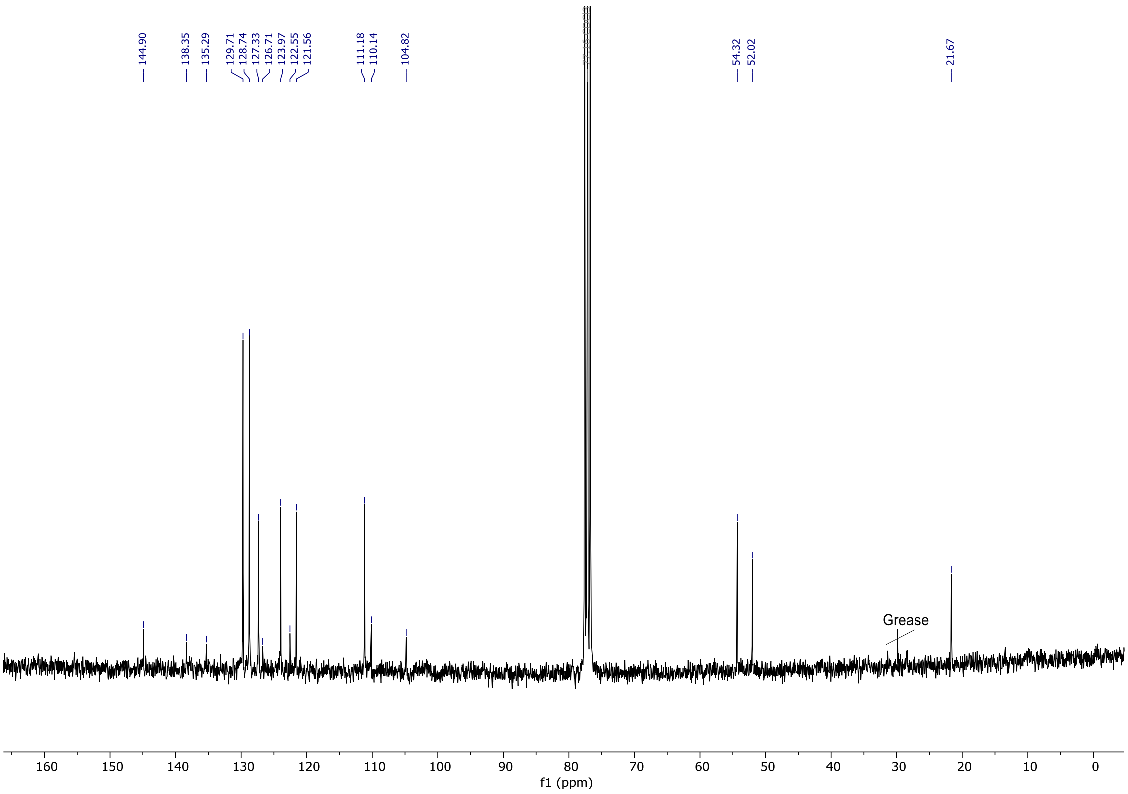

^{1}H NMR (300 MHz, $CDCl_3$) of **13am**

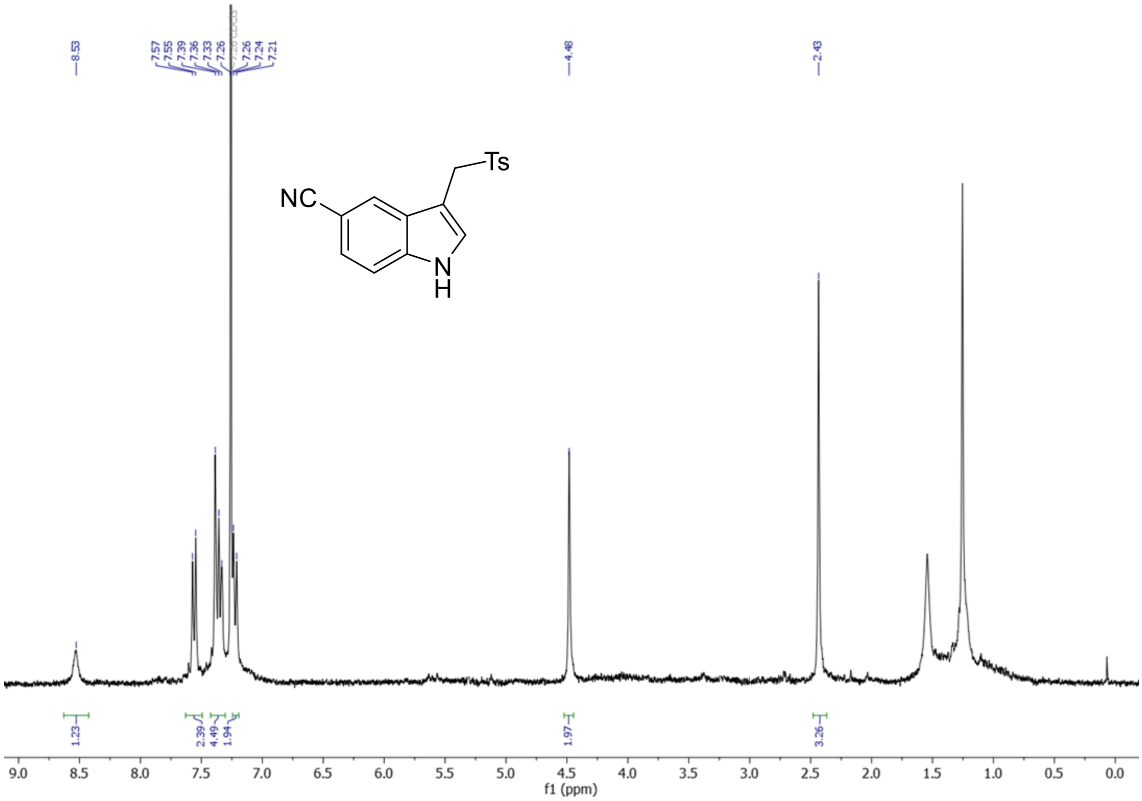

^{1}H NMR (300 MHz, Acetone-d_6) of **13an**

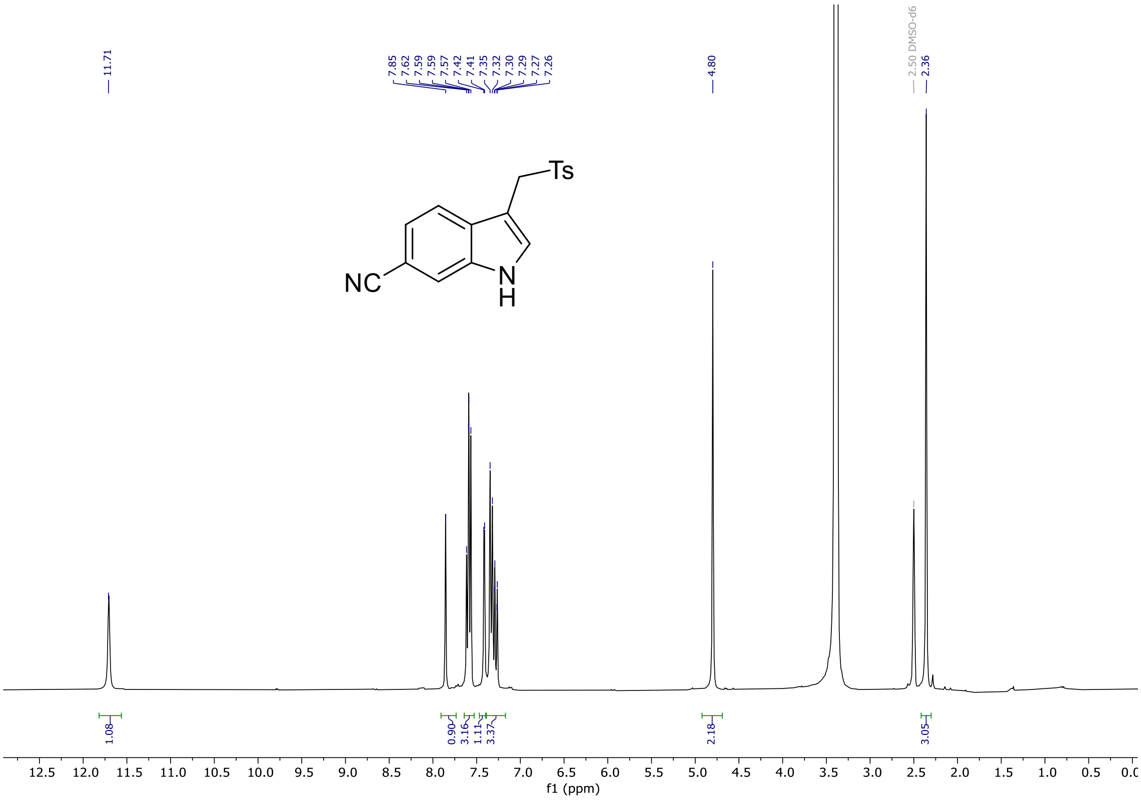

^{13}C{^{1}H} NMR (75 MHz, Acetone-d_6) of **13an**

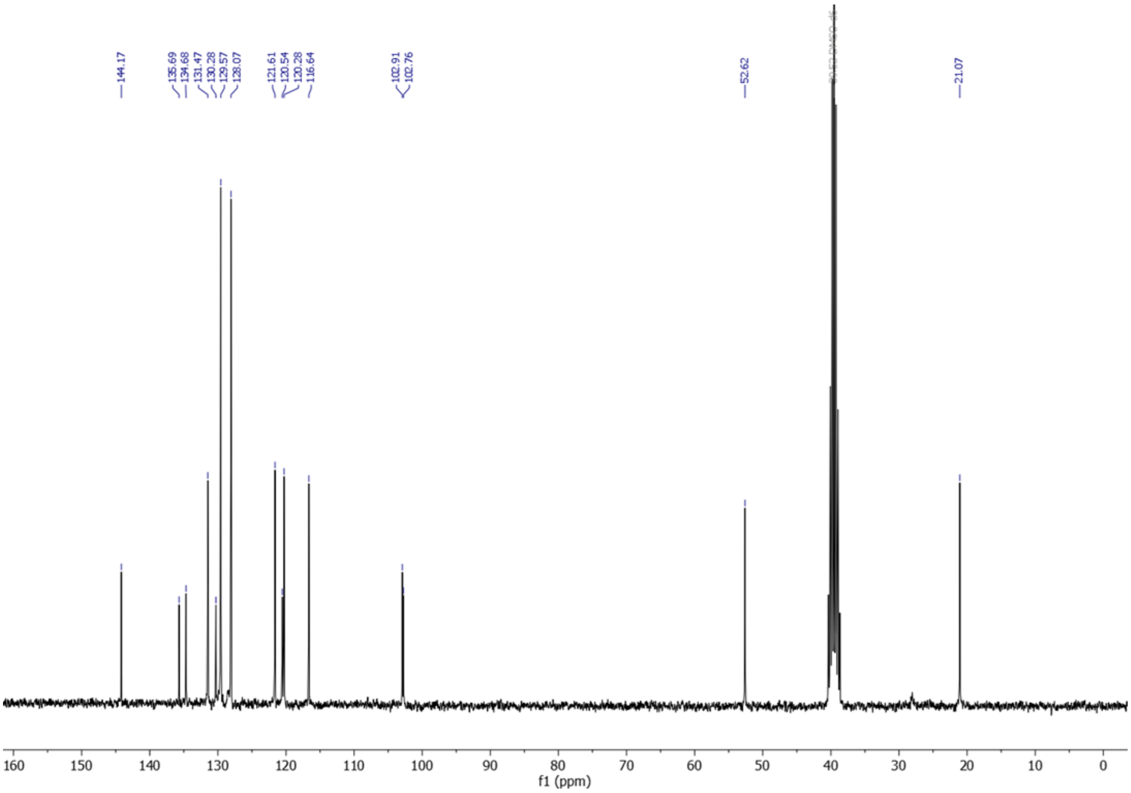

^{1}H NMR (300 MHz, $CDCl_3$) of **13ap**

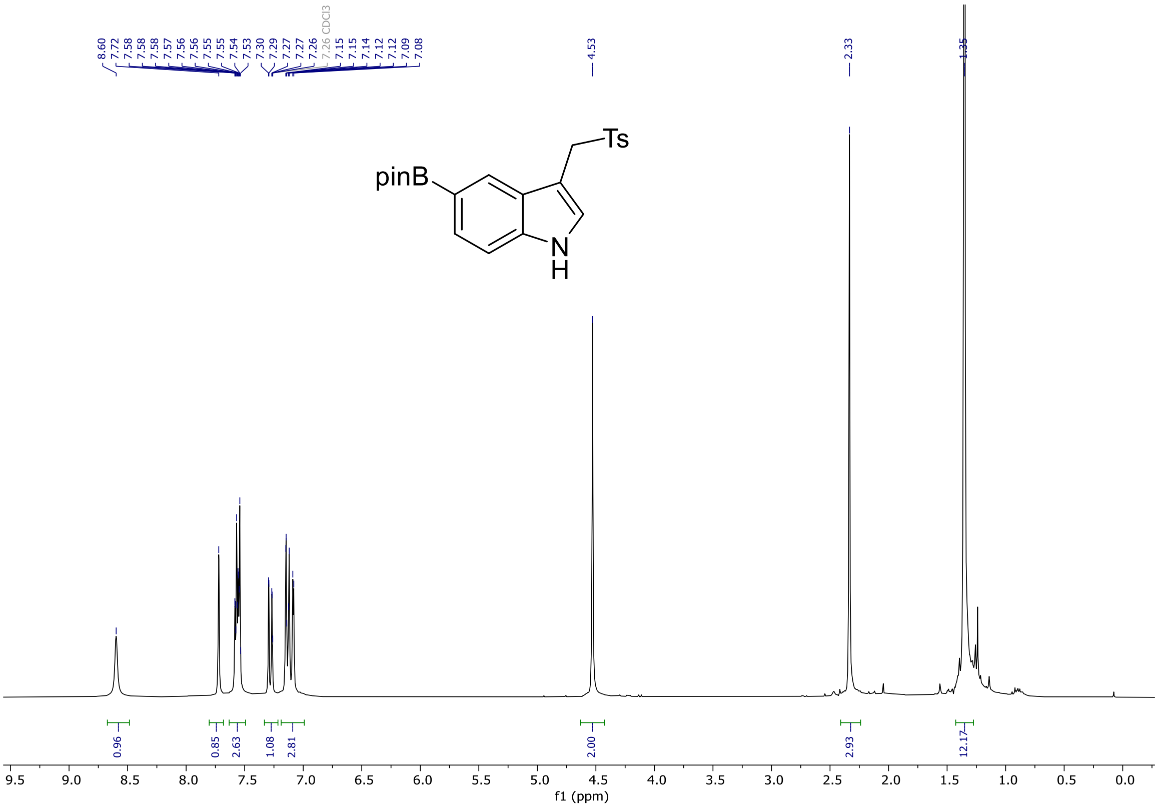

$^{13}C\{^{1}H\}$ NMR (75 MHz, $CDCl_3$) of **13ap**

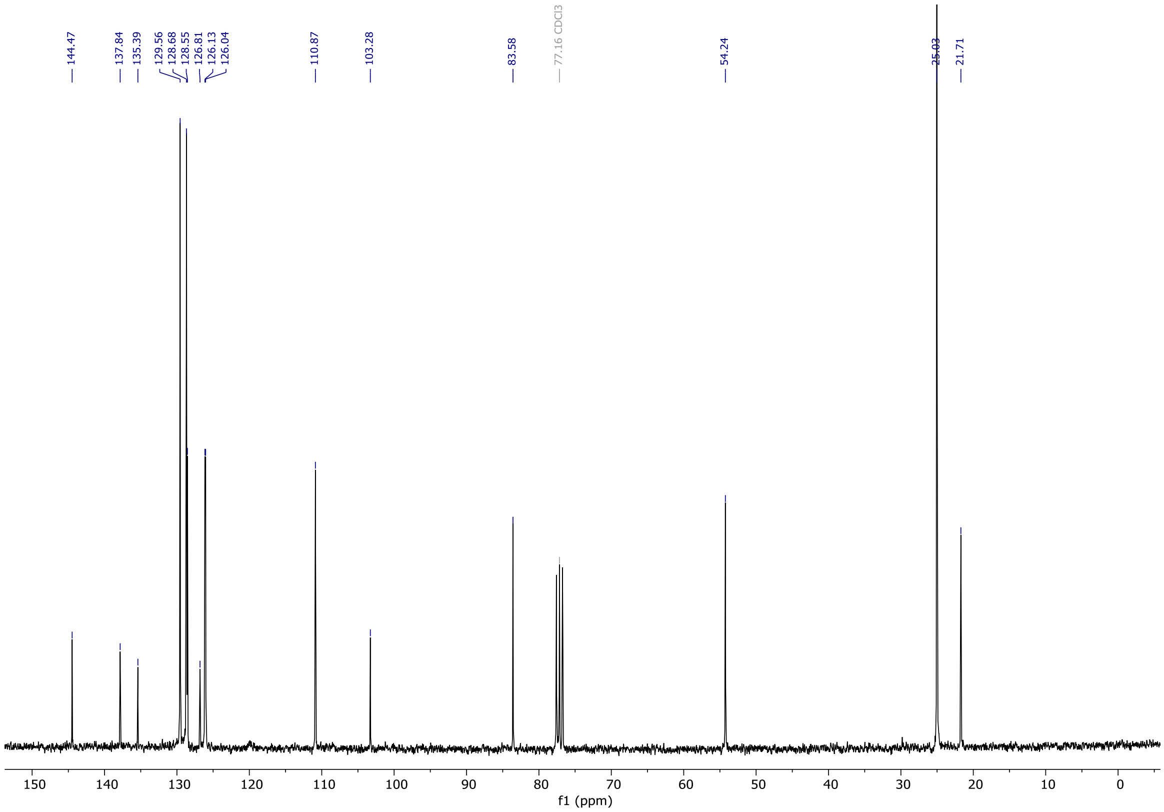

^{1}H NMR (300 MHz, $CDCl_3$) of **16aq**

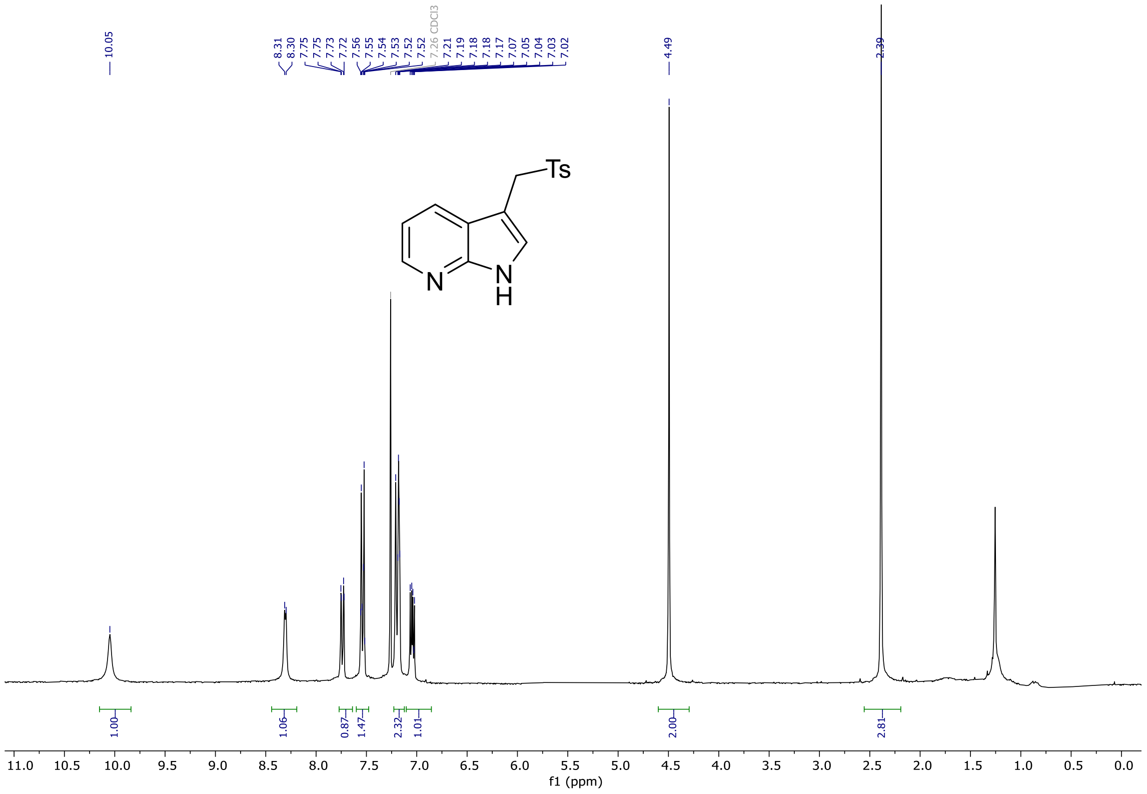

^{13}C{^{1}H} NMR (75 MHz, $CDCl_3$) of **16aq**

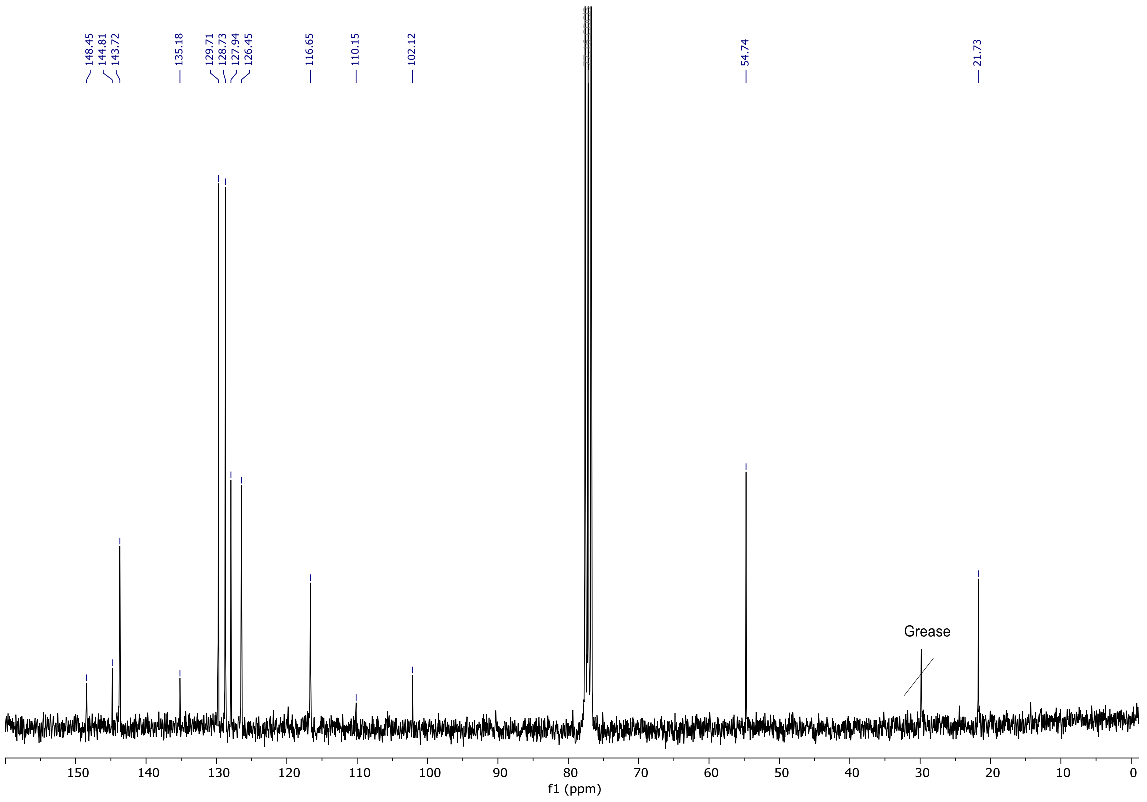

^{1}H NMR (300 MHz, Acetone-d_6) of **13ar**

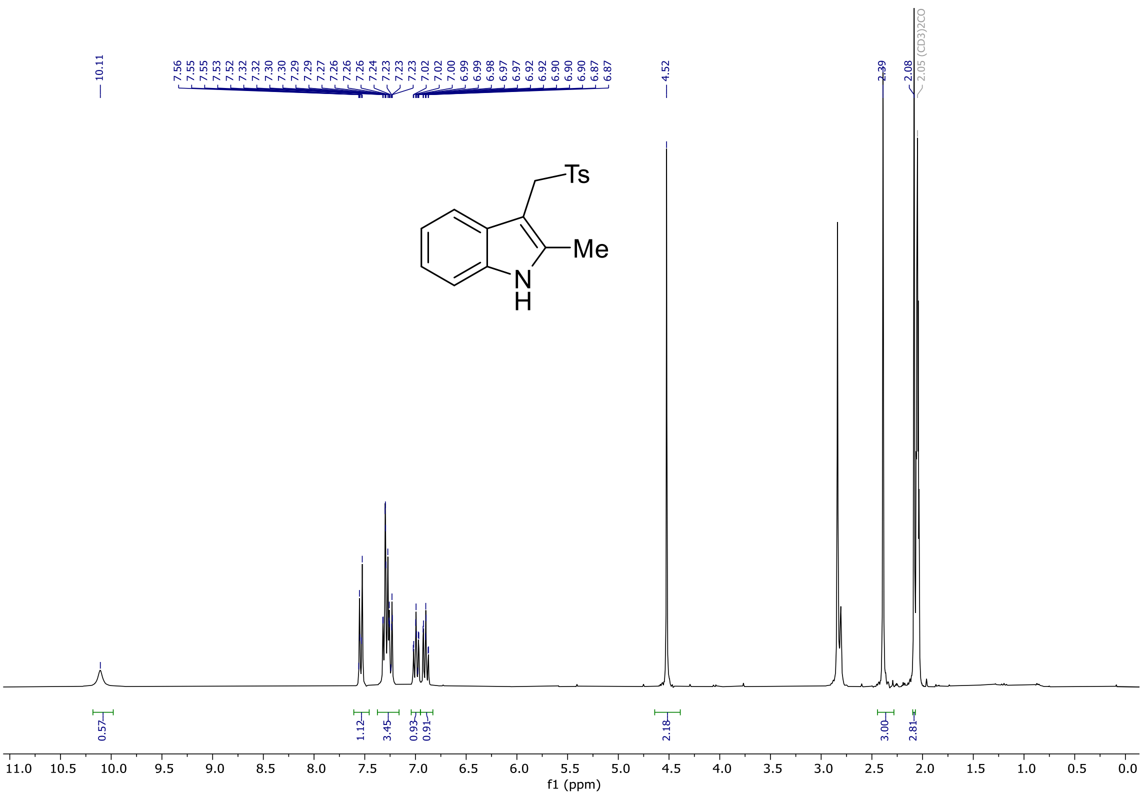

^{1}H NMR (300 MHz, $CDCl_3$) of **13as**

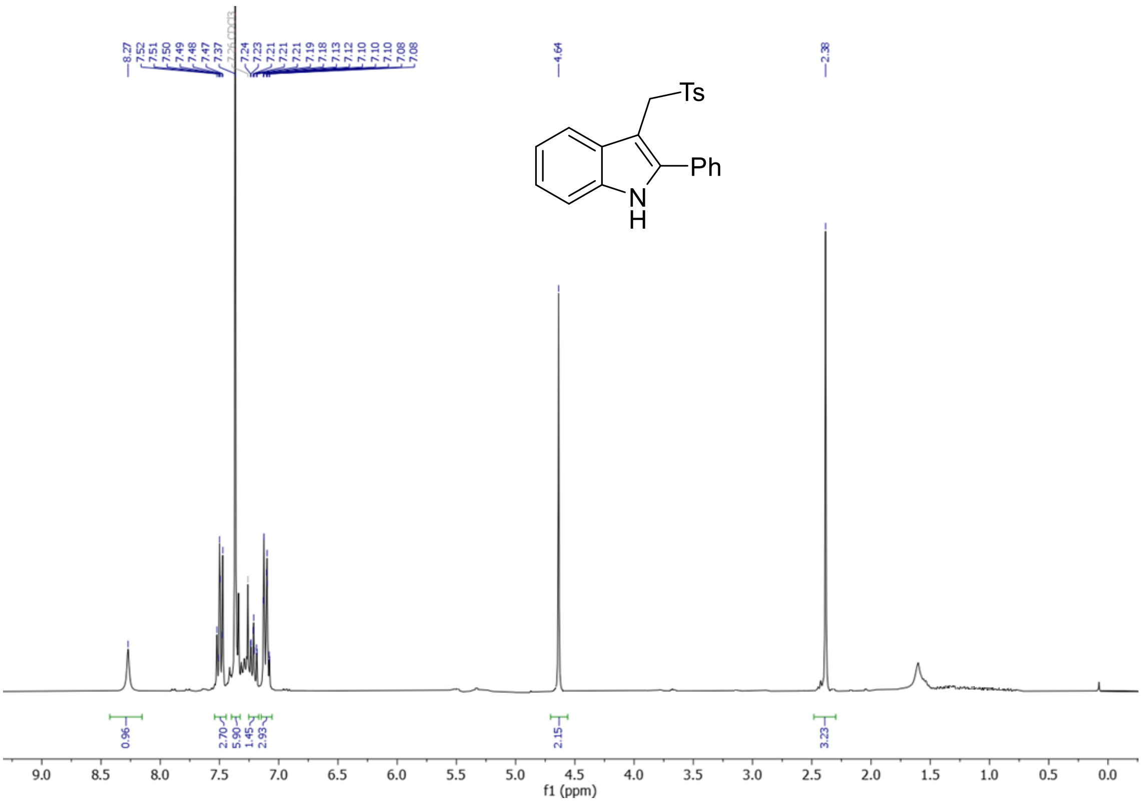

^{1}H NMR (300 MHz, $CDCl_3$) of **13at**

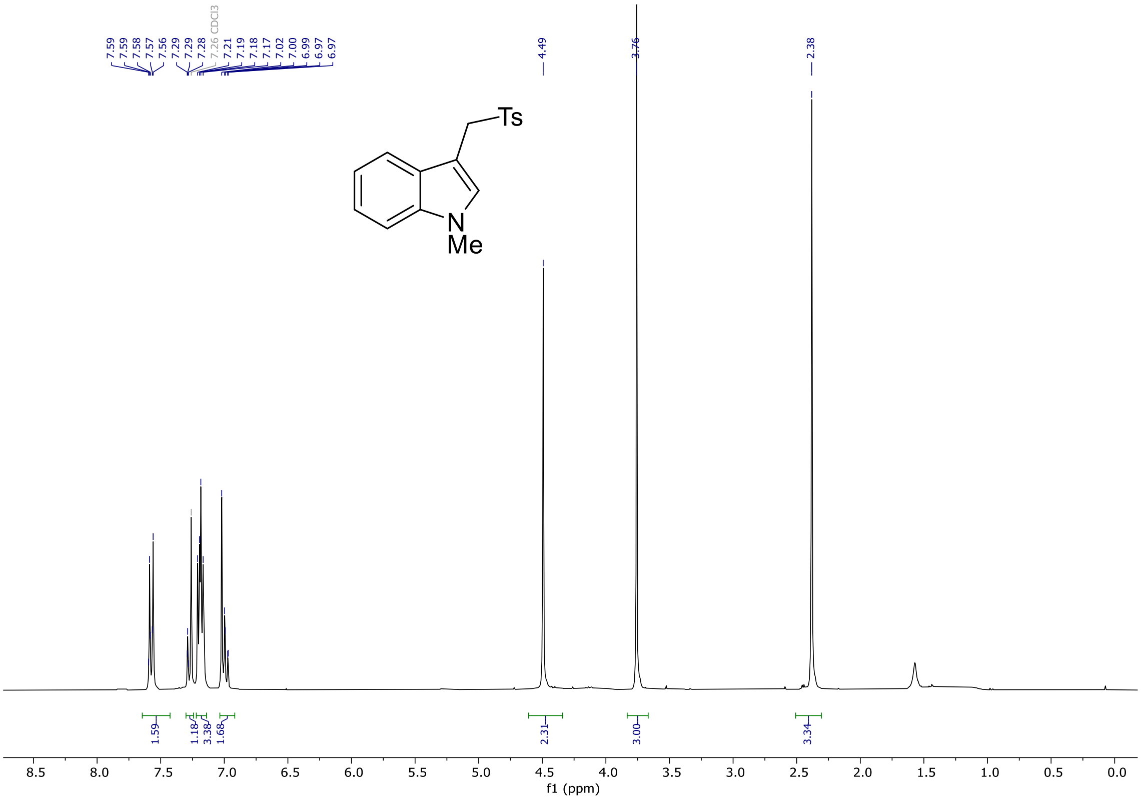

^{1}H NMR (300 MHz, $CDCl_3$) of **14aa**

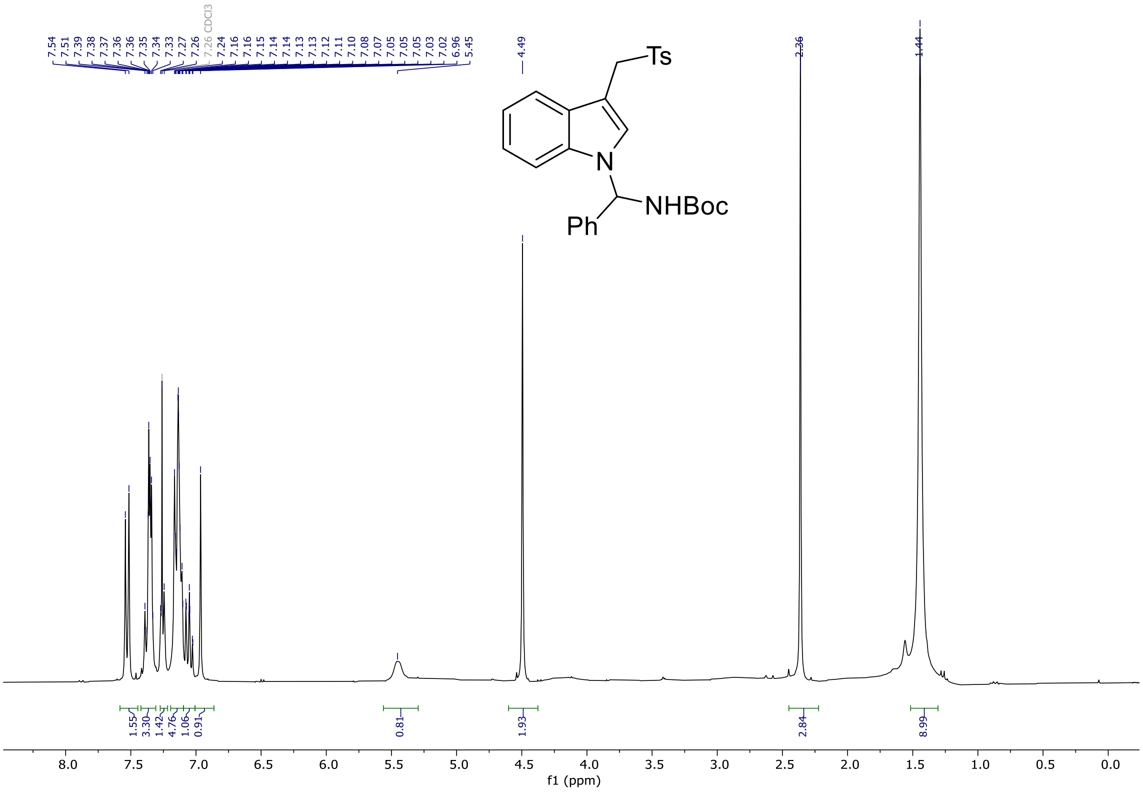

^{13}C{^{1}H} NMR (75 MHz, $CDCl_3$) of **14aa**

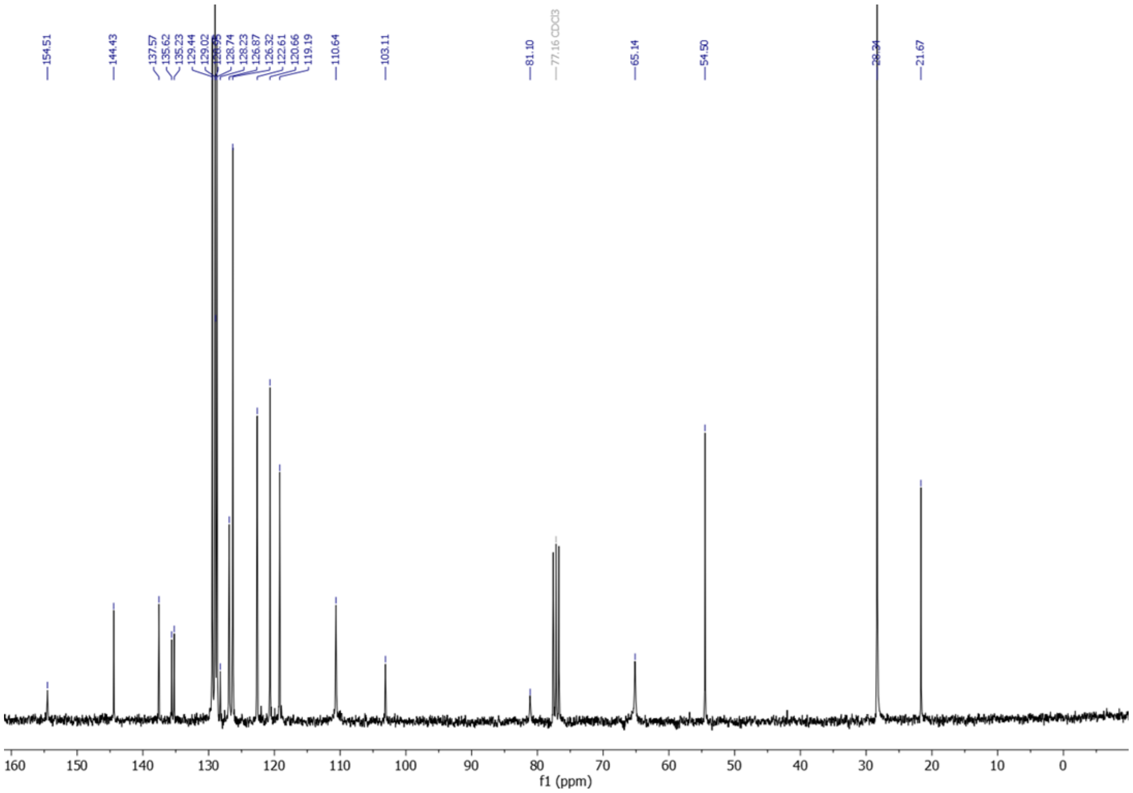

^{1}H NMR (300 MHz, $CDCl_3$) of **14ba**

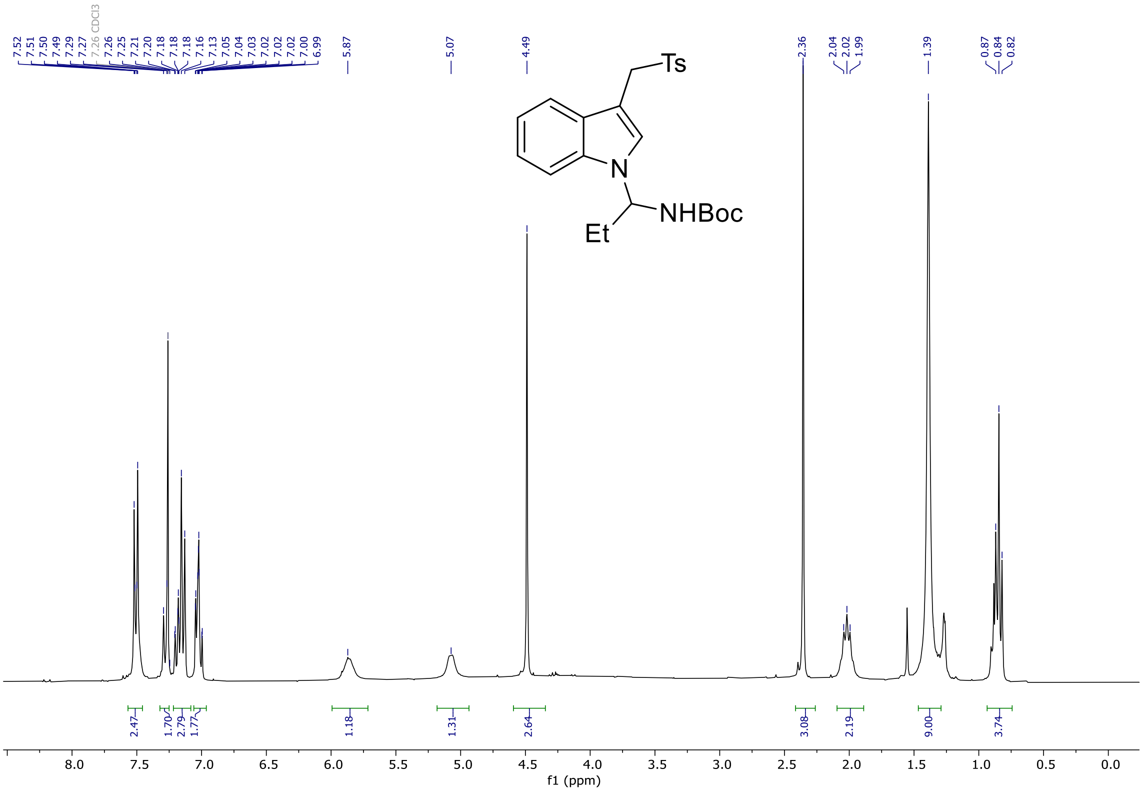

$^{13}C\{^{1}H\}$ NMR (75 MHz, $CDCl_3$) of **14ba**

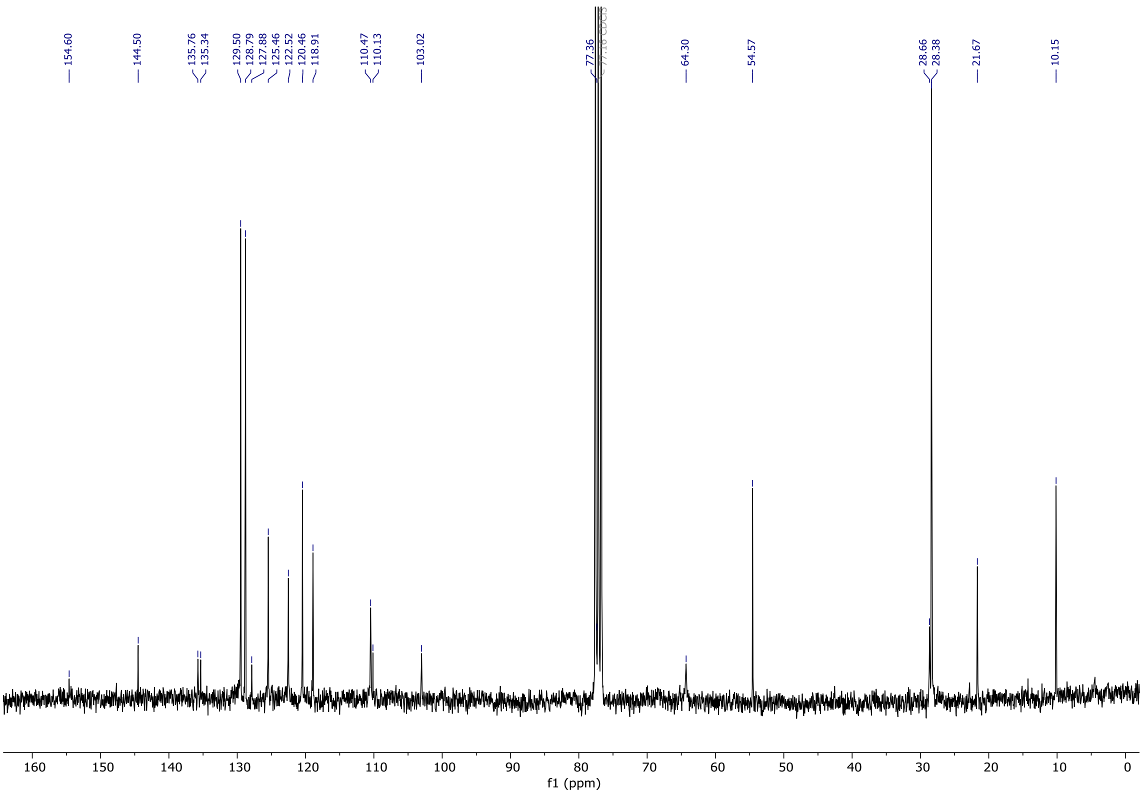

^{1}H NMR (300 MHz, $CDCl_3$) of **14ca**

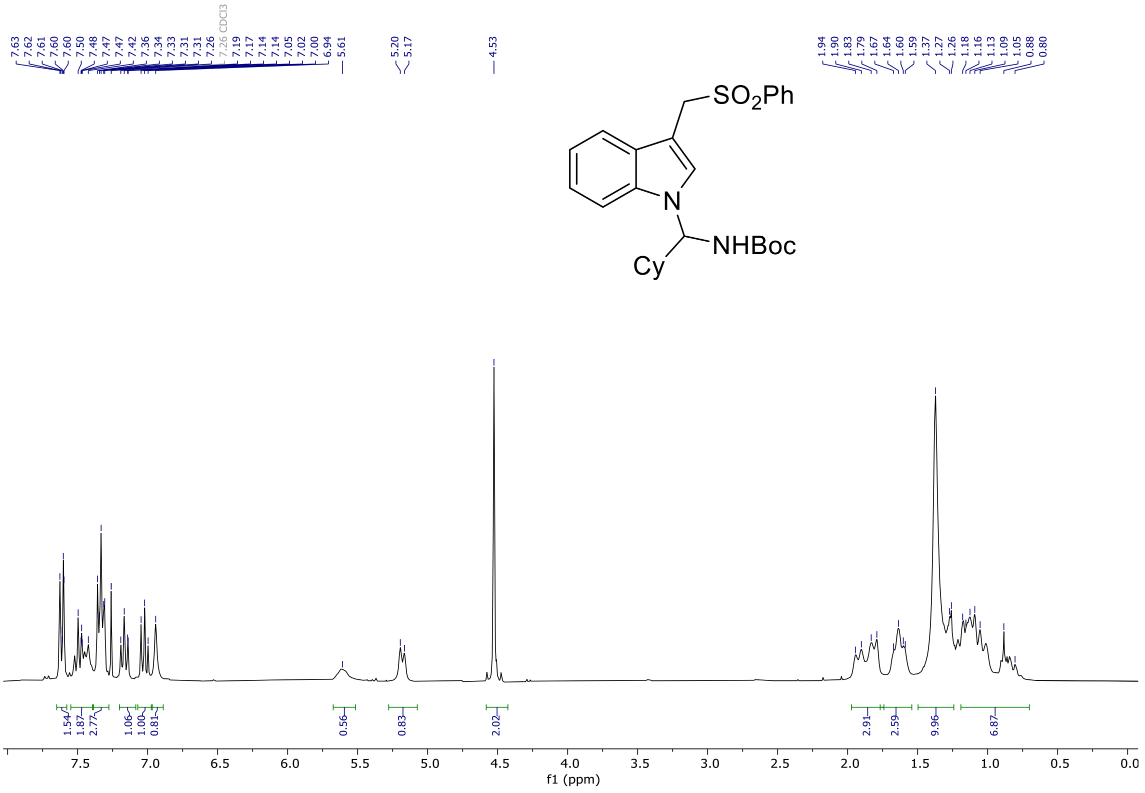

$^{13}C\{^{1}H\}$ NMR (75 MHz, $CDCl_3$) of **14ca**

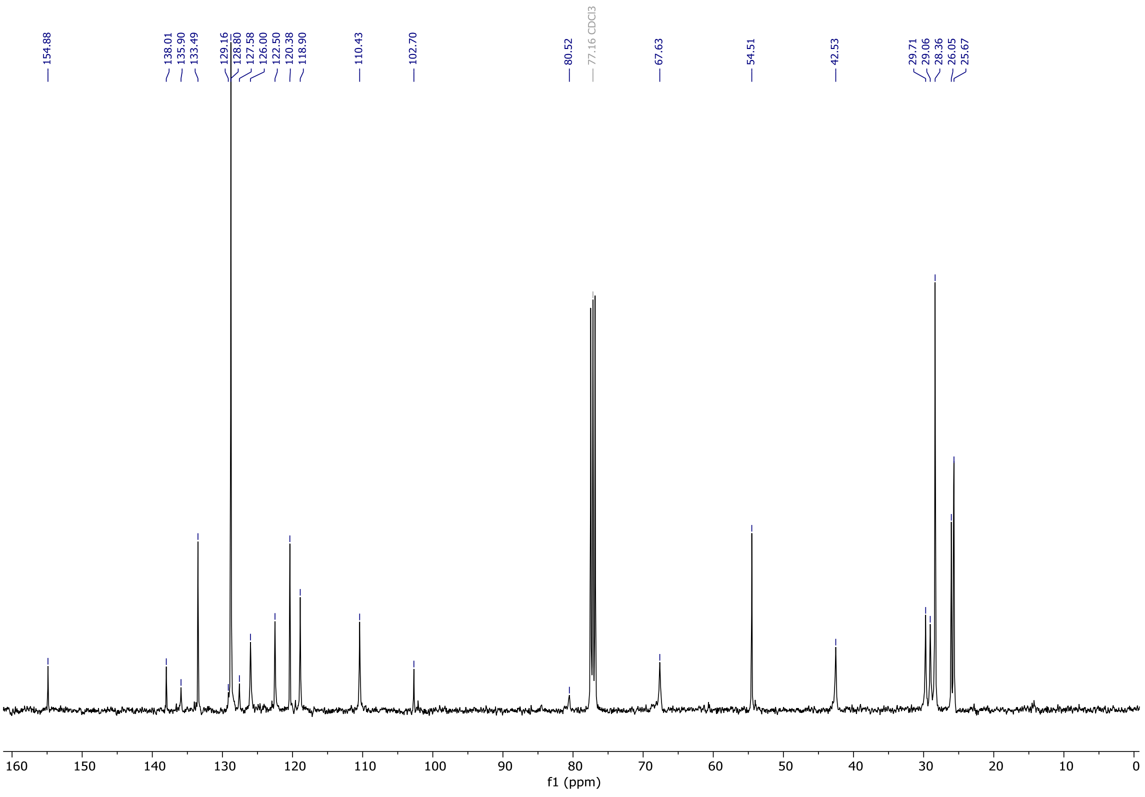

^{1}H NMR (300 MHz, $CDCl_3$) of **14da**

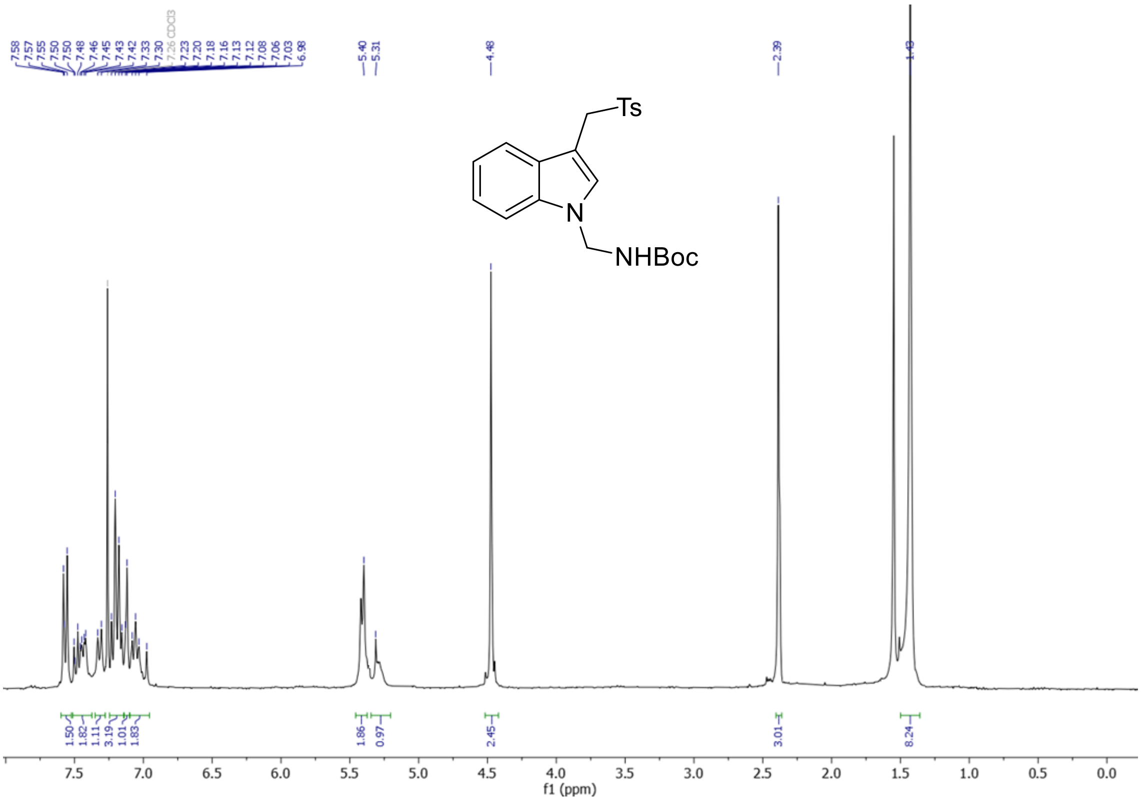

^{13}C{^{1}H} NMR (75 MHz, $CDCl_3$) of **14da**

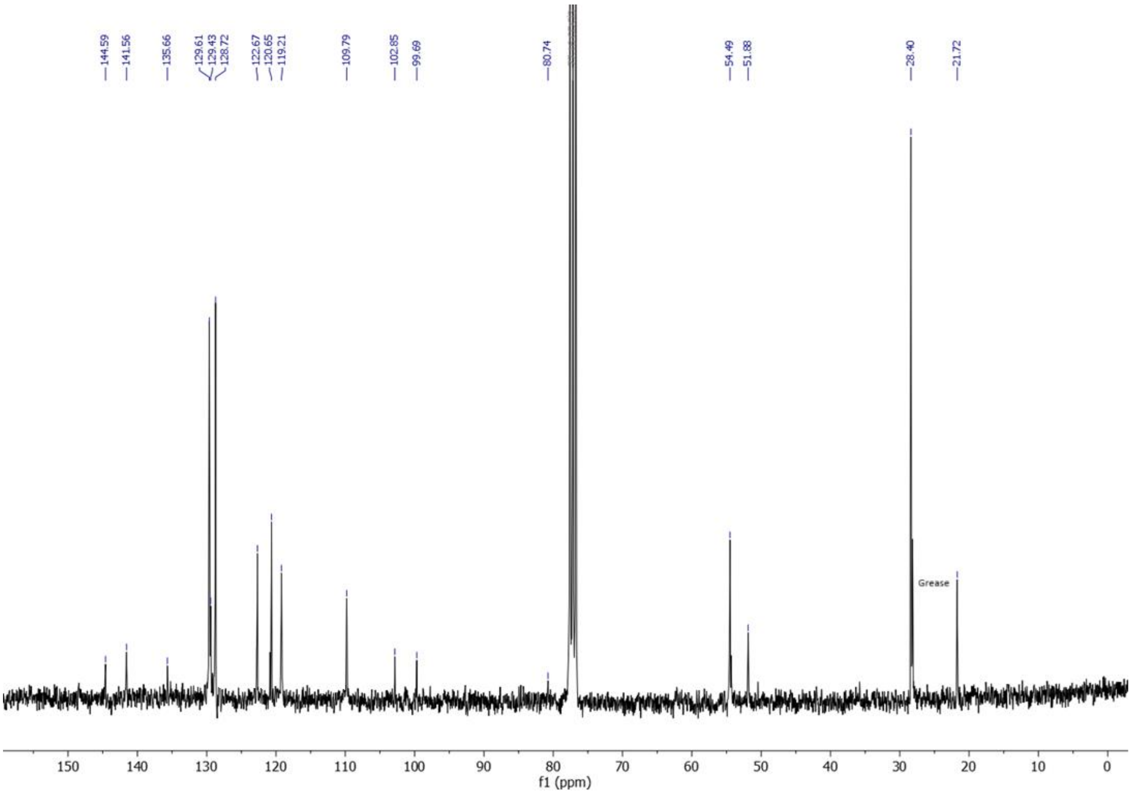

^{1}H NMR (300 MHz, $CDCl_3$) of **14ea**

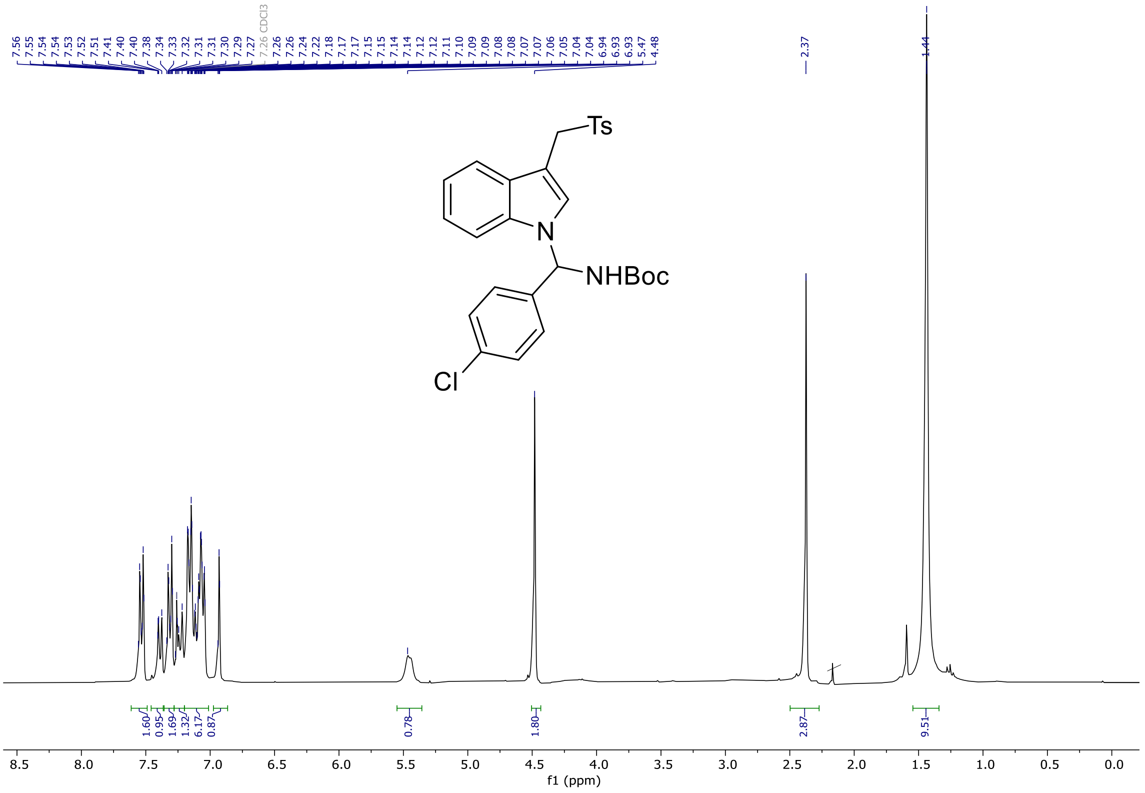

$^{13}C\{^{1}H\}$ NMR (75 MHz, $CDCl_3$) of **14ea**

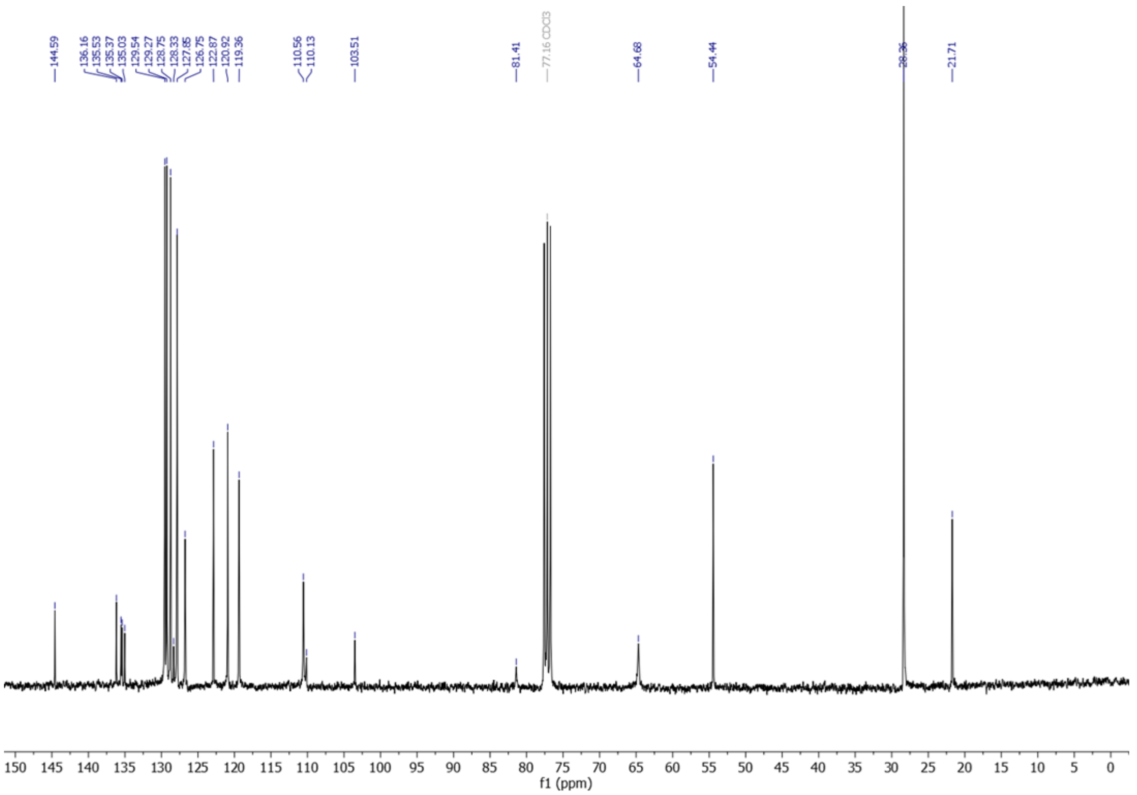

^{1}H NMR (300 MHz, $CDCl_3$) of **14fa**

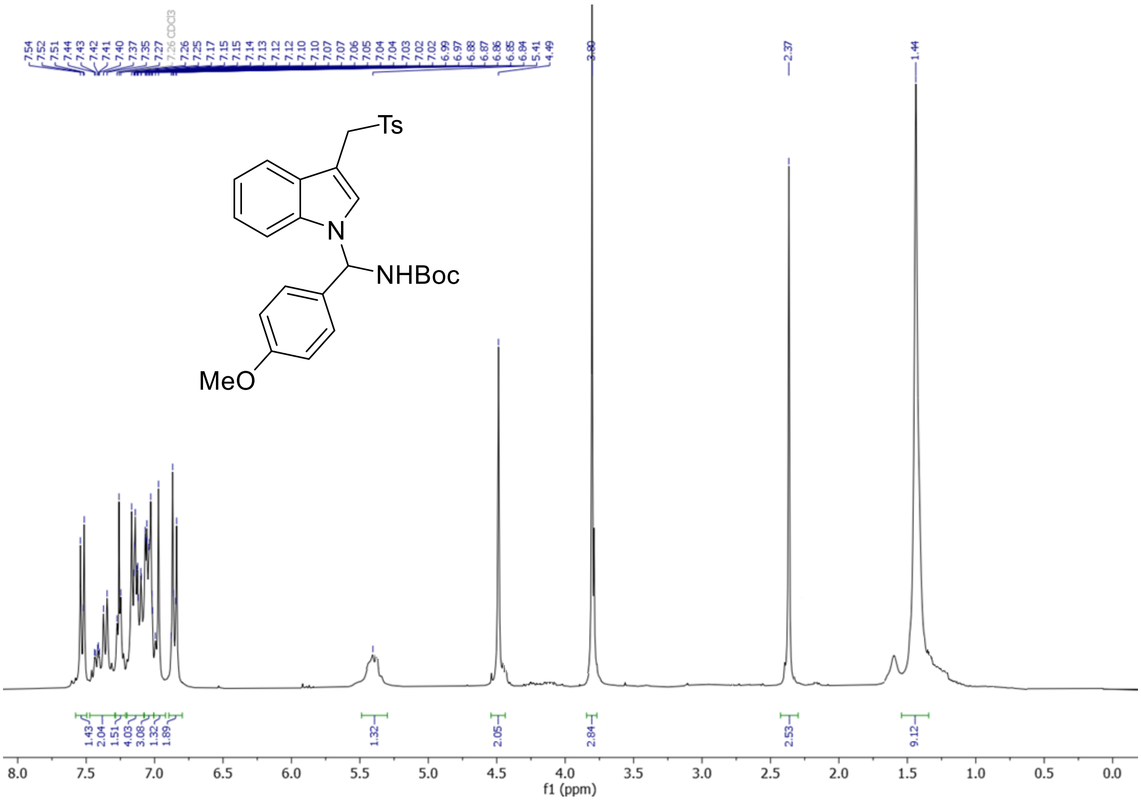

$^{13}C\{^{1}H\}$ NMR (75 MHz, $CDCl_3$) of **14fa**

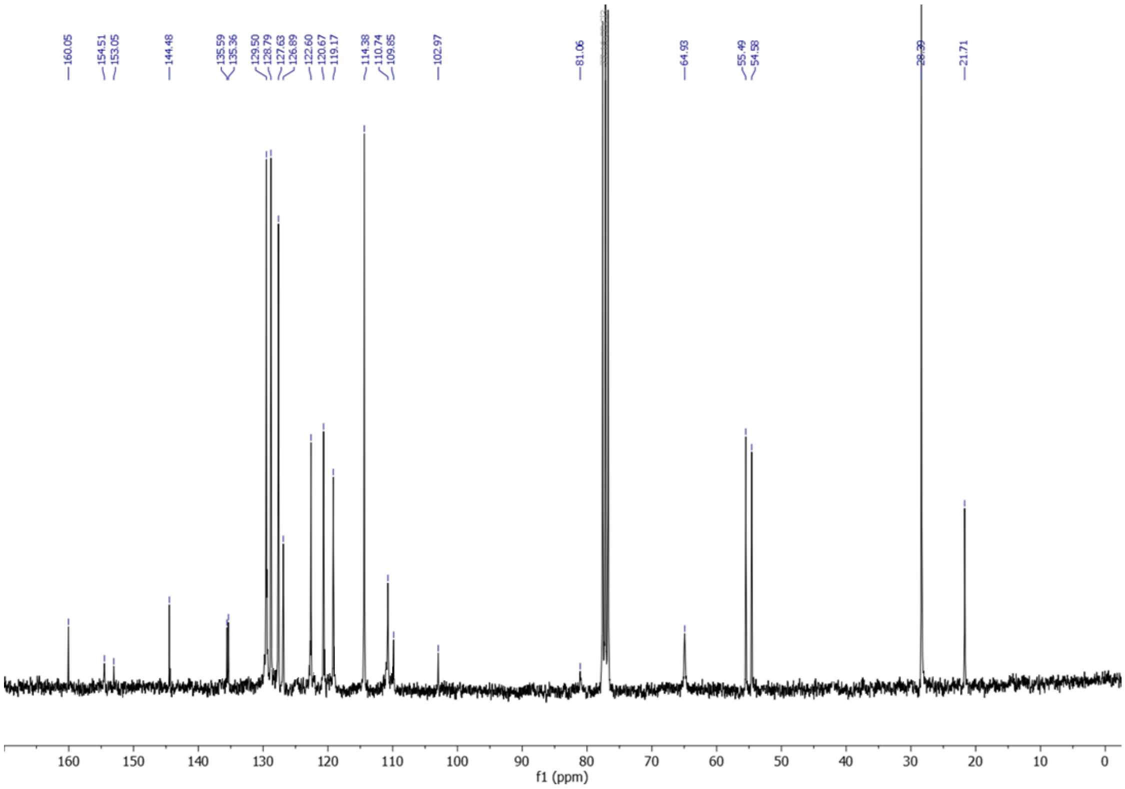

^{1}H NMR (300 MHz, $CDCl_3$) of **14ab**

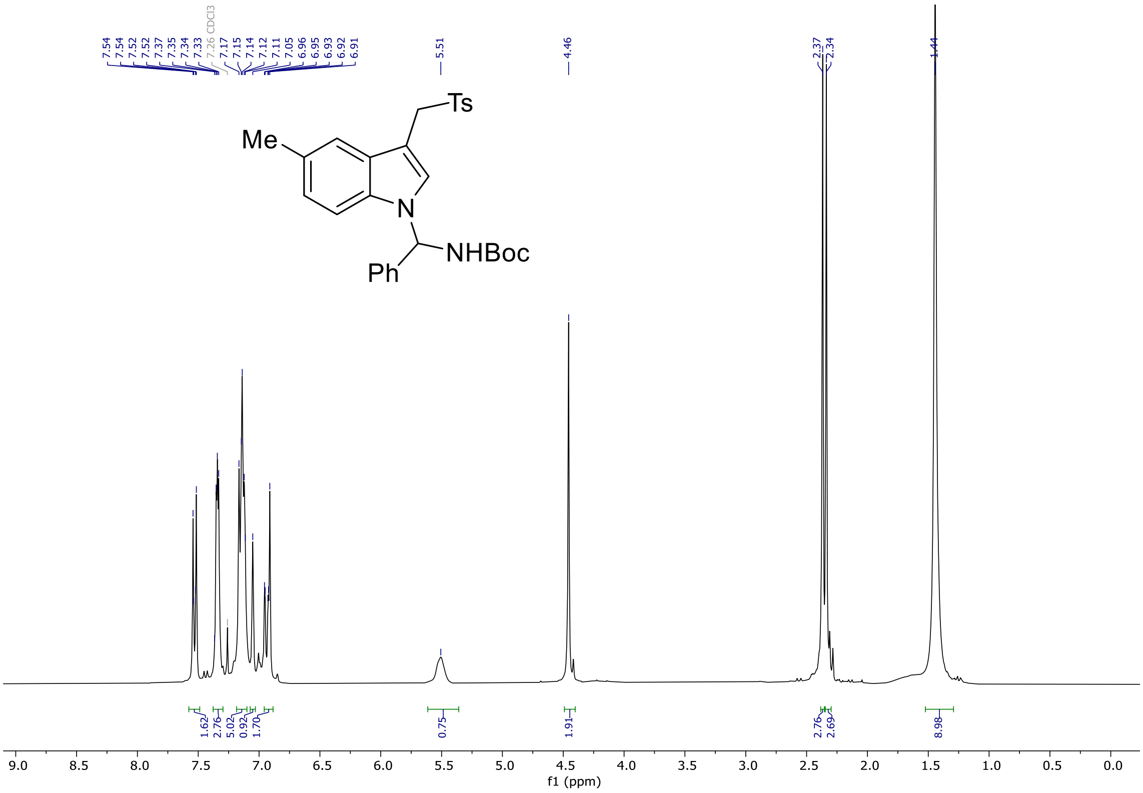

$^{13}C\{^{1}H\}$ NMR (75 MHz, $CDCl_3$) of **14ab**

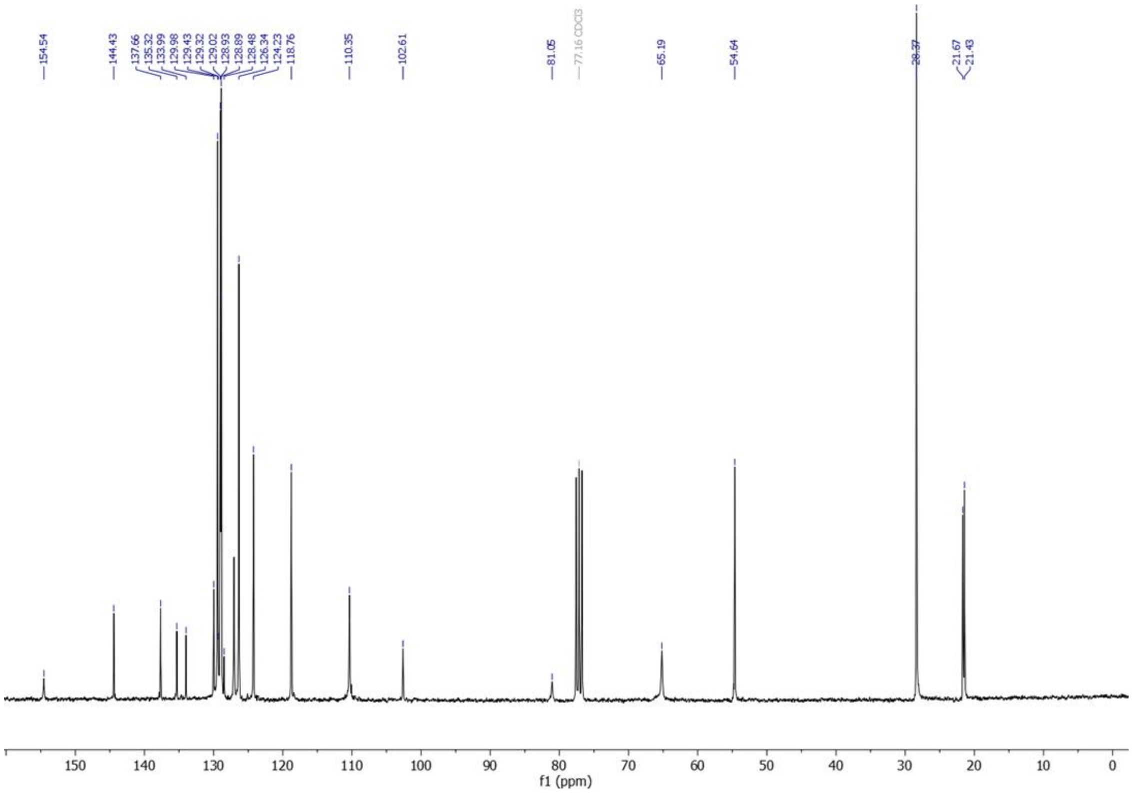

^{1}H NMR (300 MHz, $CDCl_3$) of **14ac**

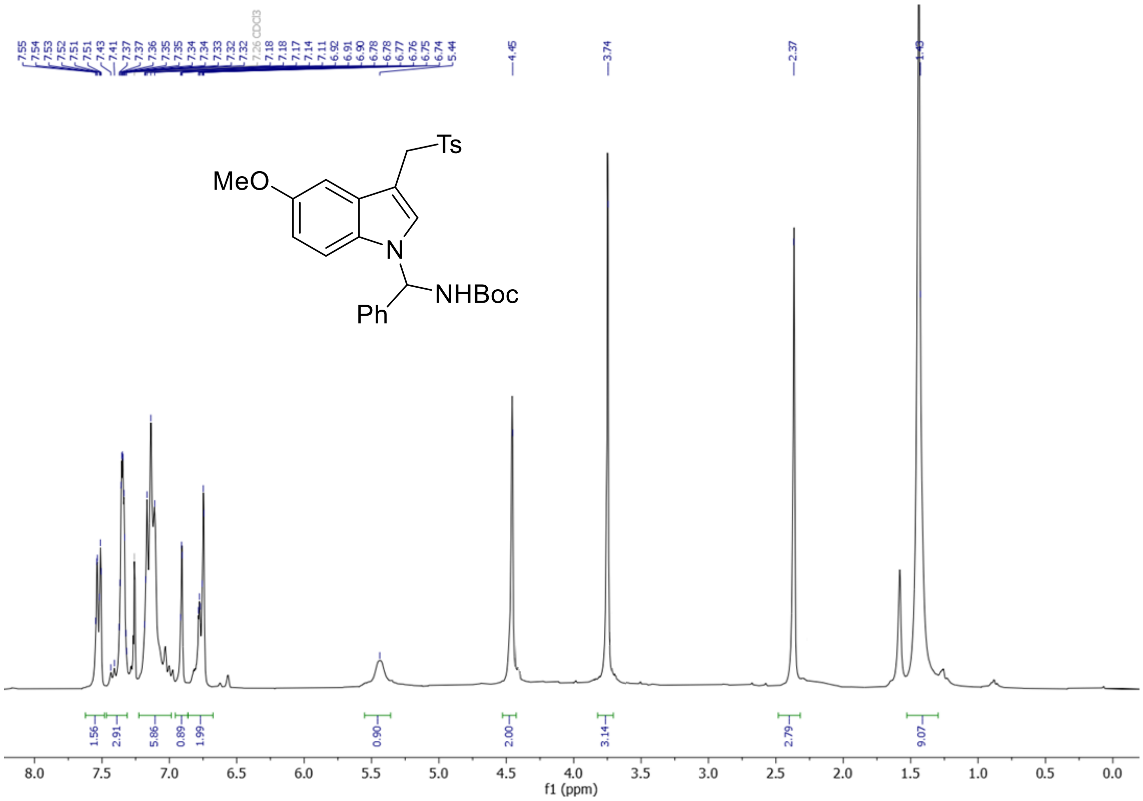

^{13}C{^{1}H} NMR (75 MHz, $CDCl_3$) of **14ac**

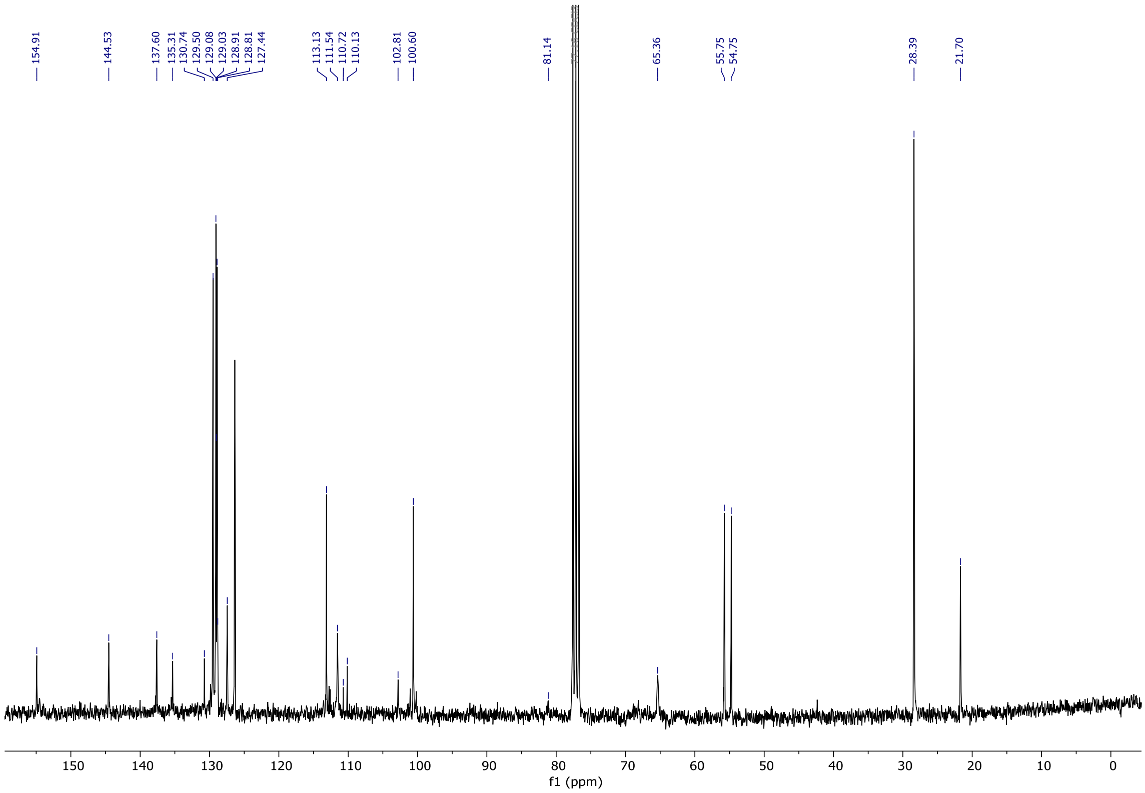

^{1}H NMR (300 MHz, $CDCl_3$) of **14ad**

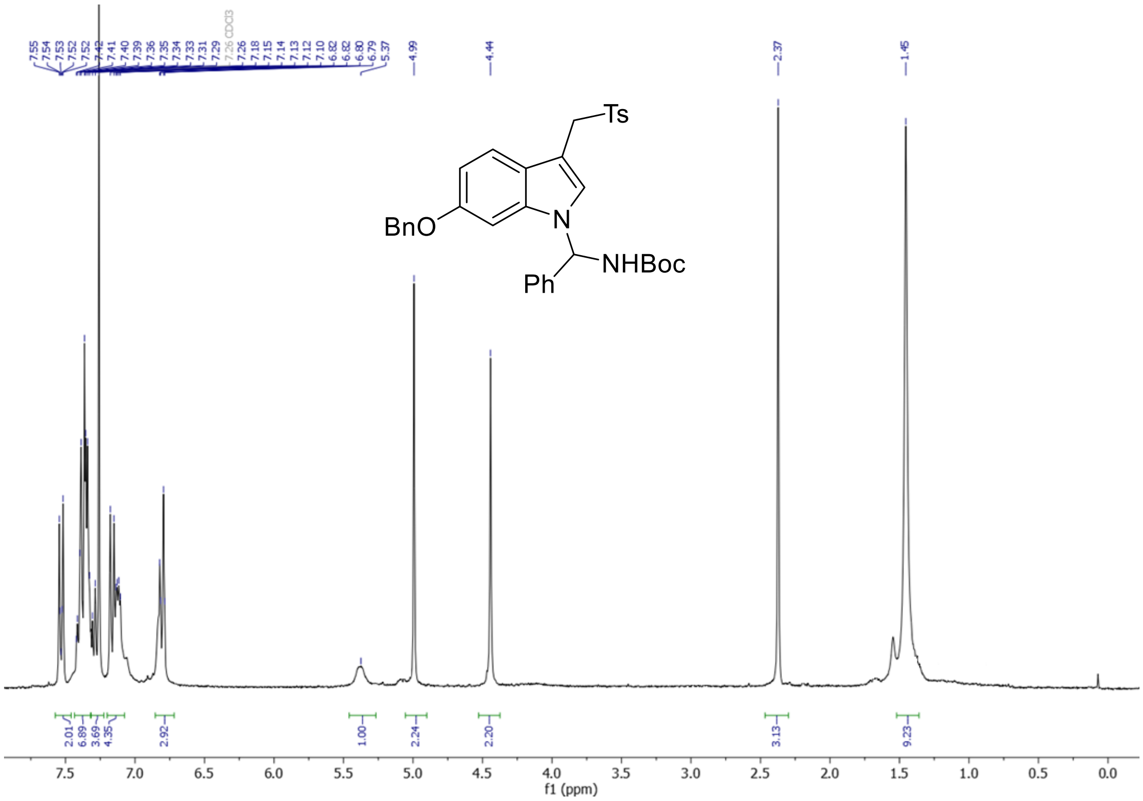

$^{13}C\{^{1}H\}$ NMR (75 MHz, $CDCl_3$) of **14ad**

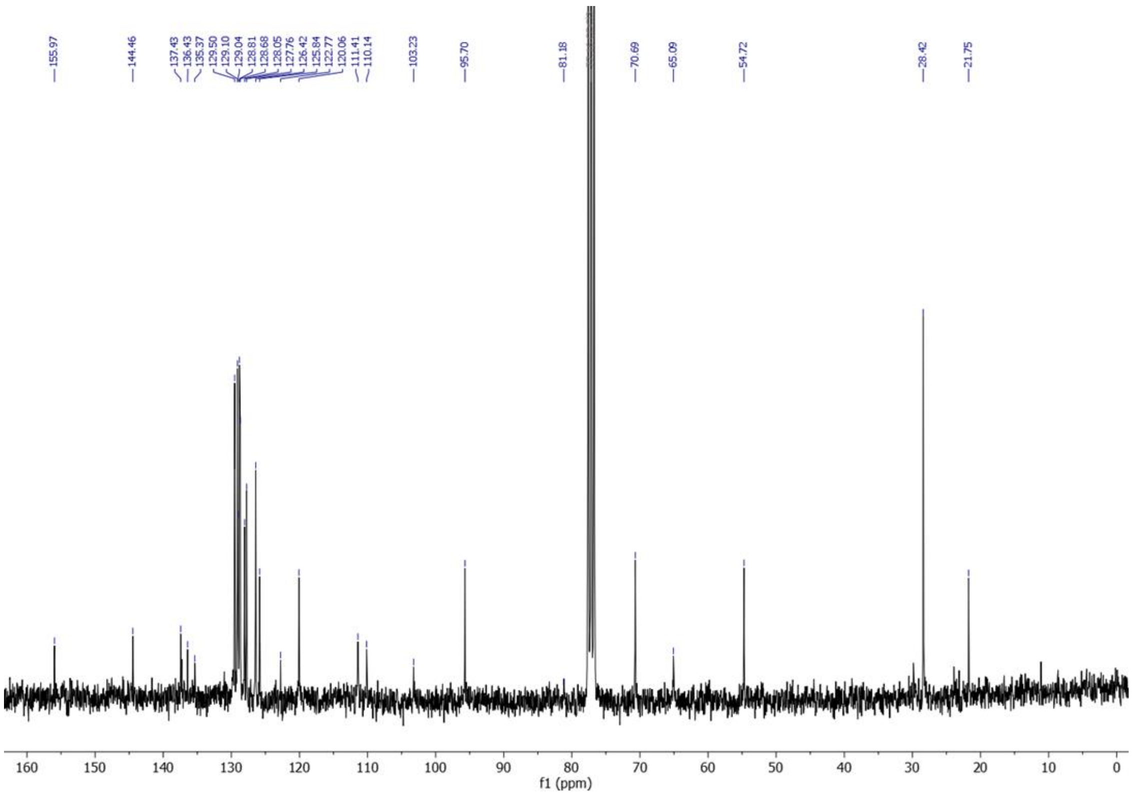

^{1}H NMR (300 MHz, $CDCl_3$) of **14ae**

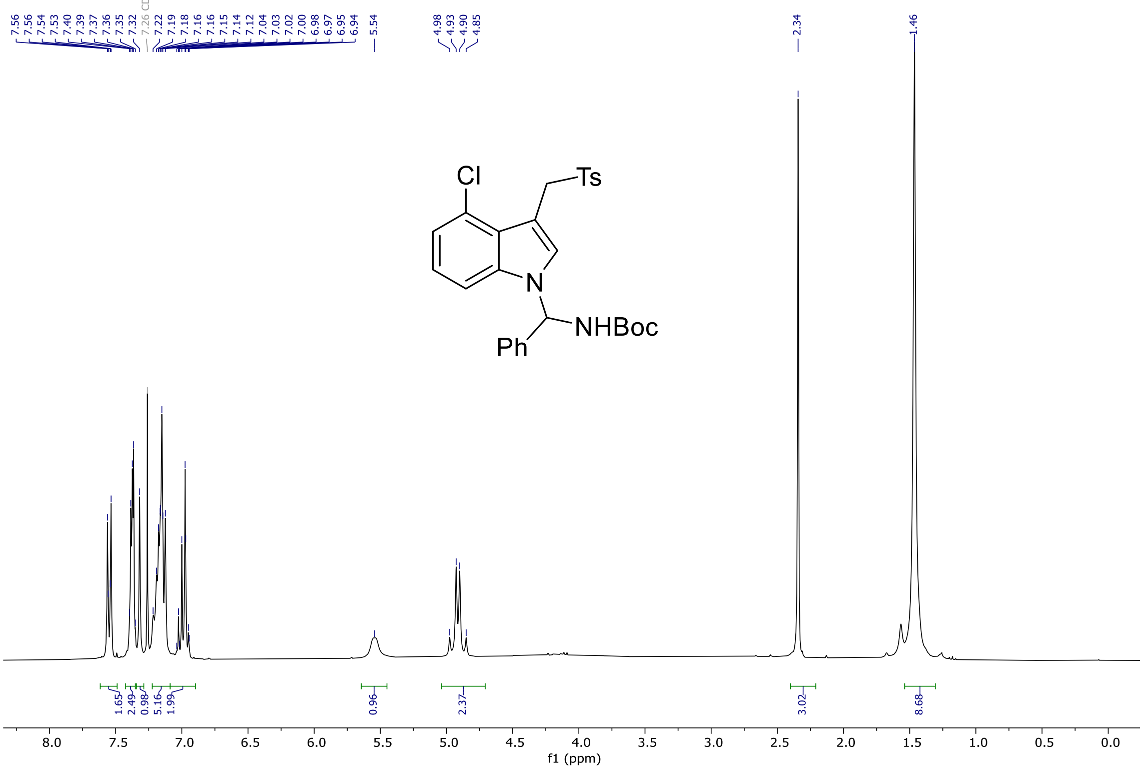

^{13}C{^{1}H} NMR (75 MHz, $CDCl_3$) of **14ae**

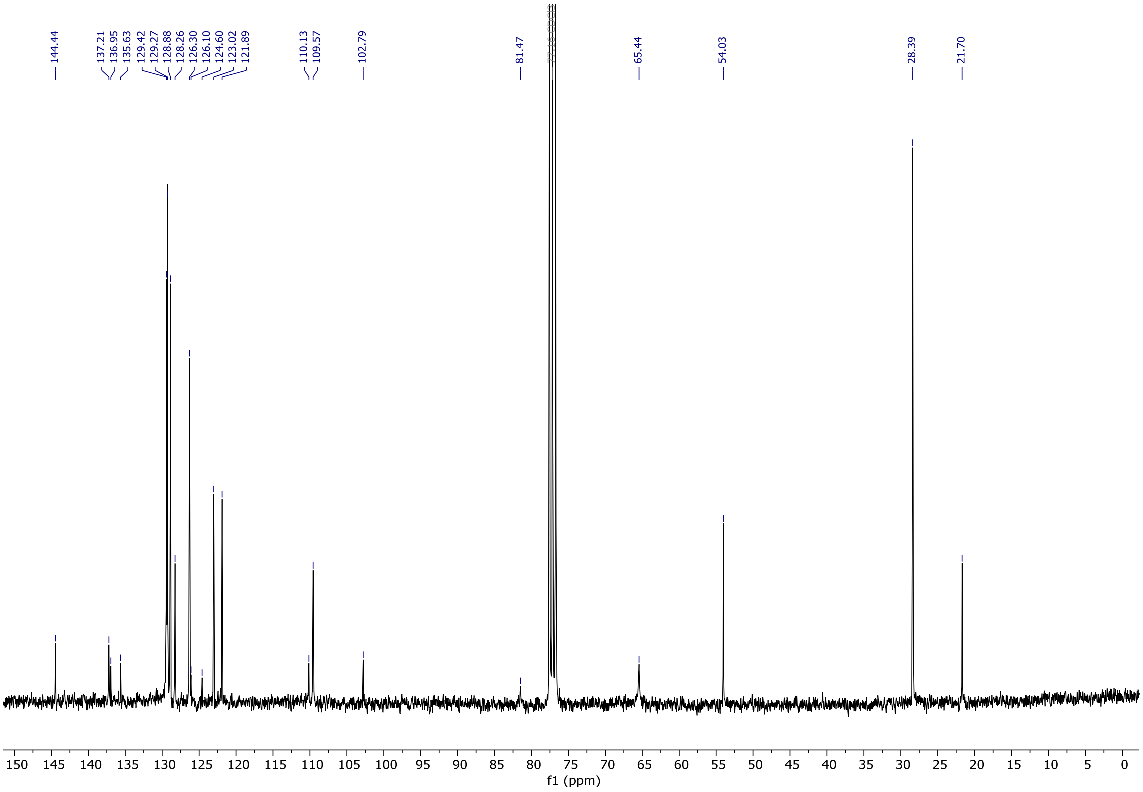

^{1}H NMR (300 MHz, $CDCl_3$) of **14af**

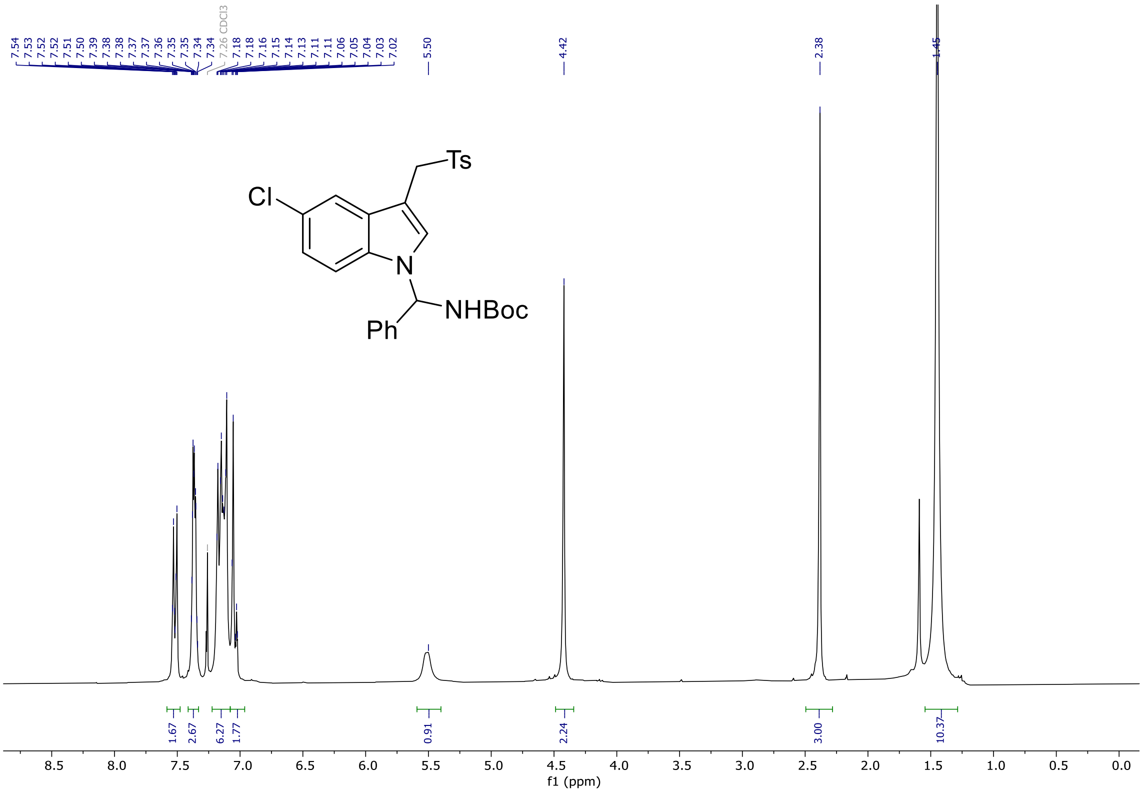

^{13}C{^{1}H} NMR (75 MHz, $CDCl_3$) of **14af**

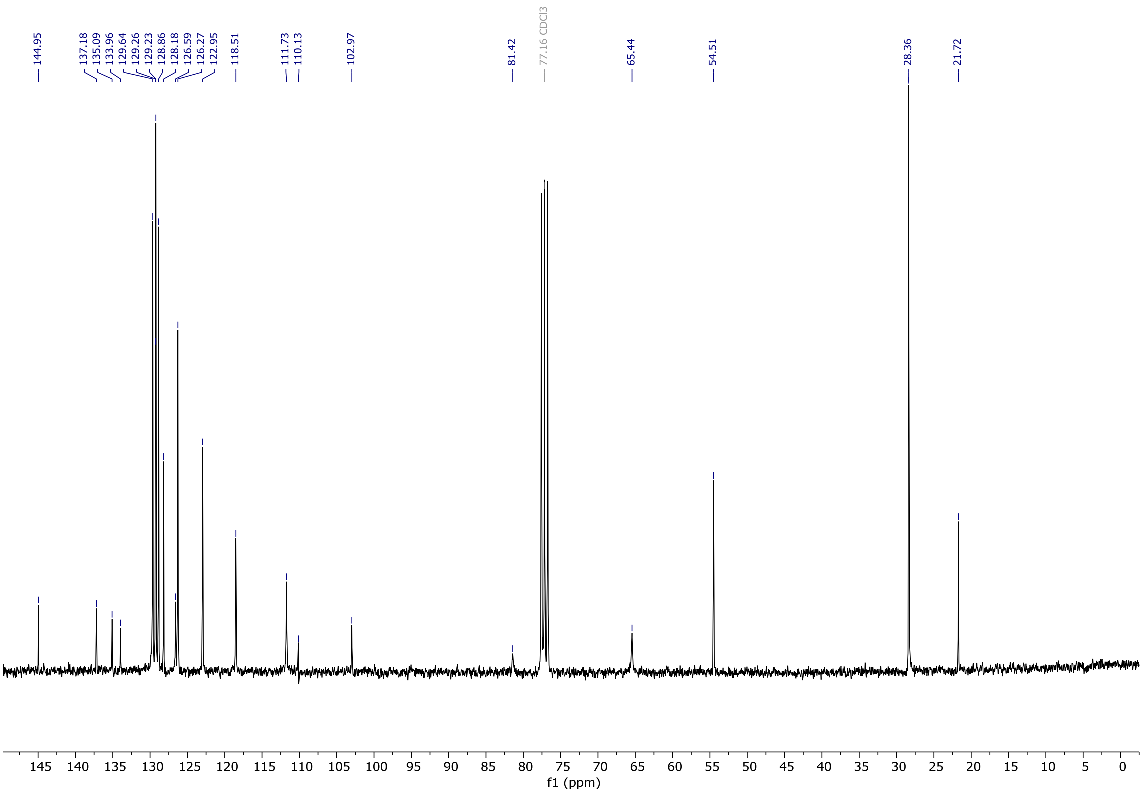

^{1}H NMR (300 MHz, $CDCl_3$) of **14ag**

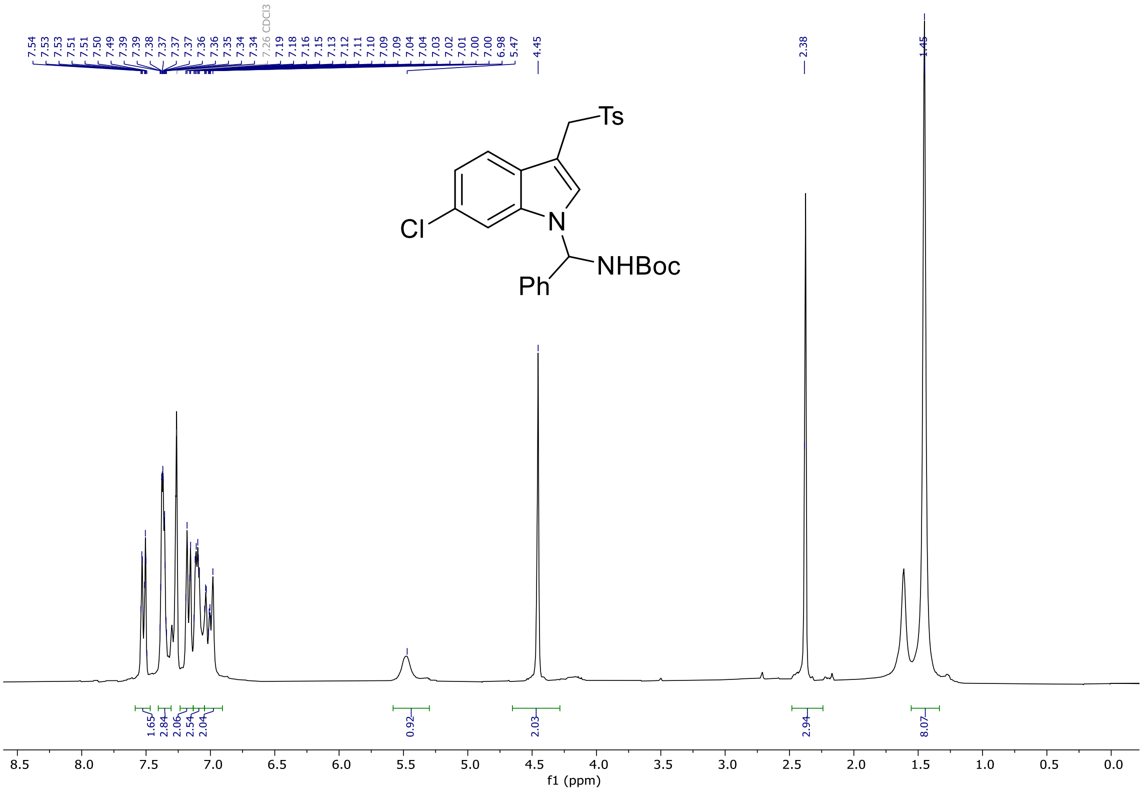

^{13}C{^{1}H} NMR (75 MHz, $CDCl_3$) of **14ag**

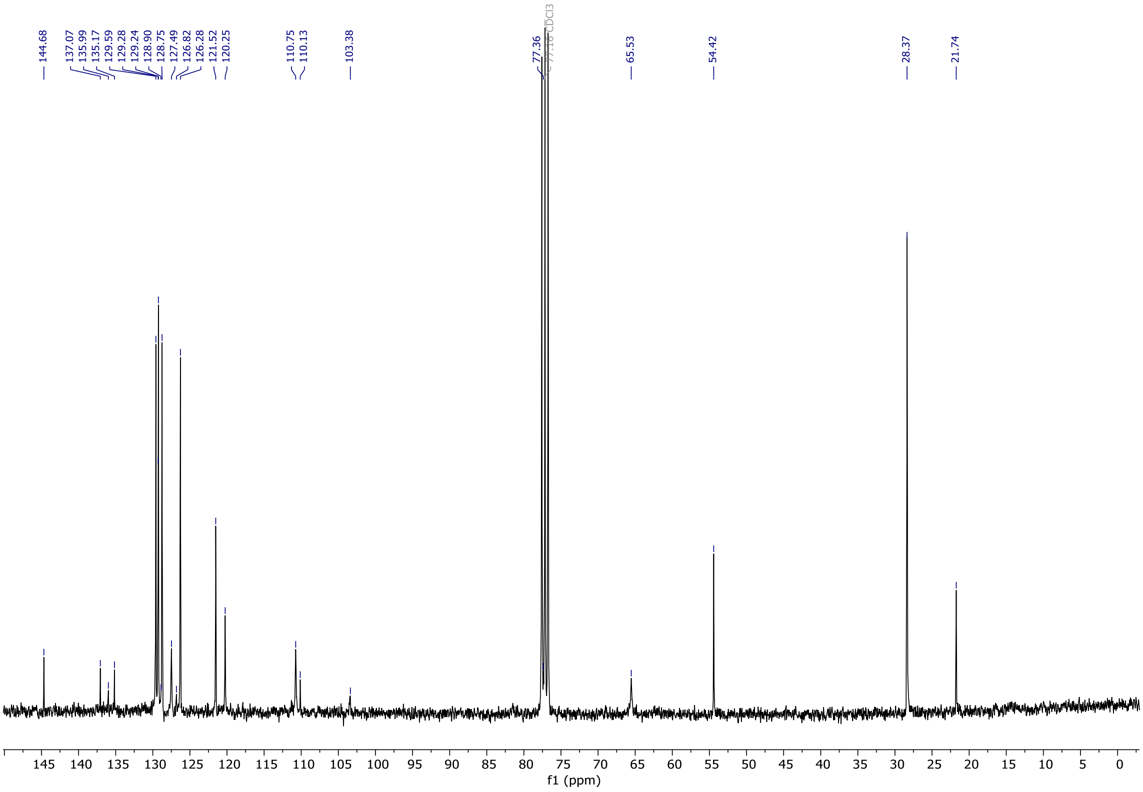

^{1}H NMR (300 MHz, $CDCl_3$) of **14aj**

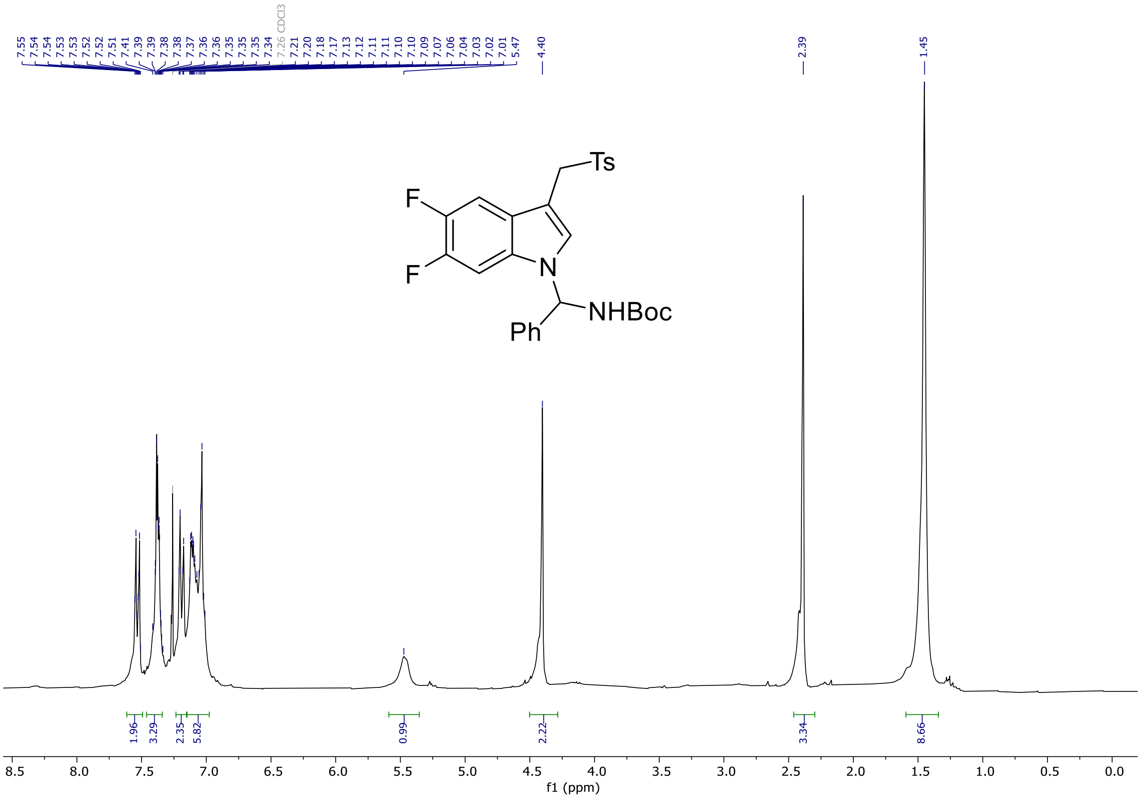

$^{13}C\{^{1}H\}$ NMR (75 MHz, $CDCl_3$) of **14aj**

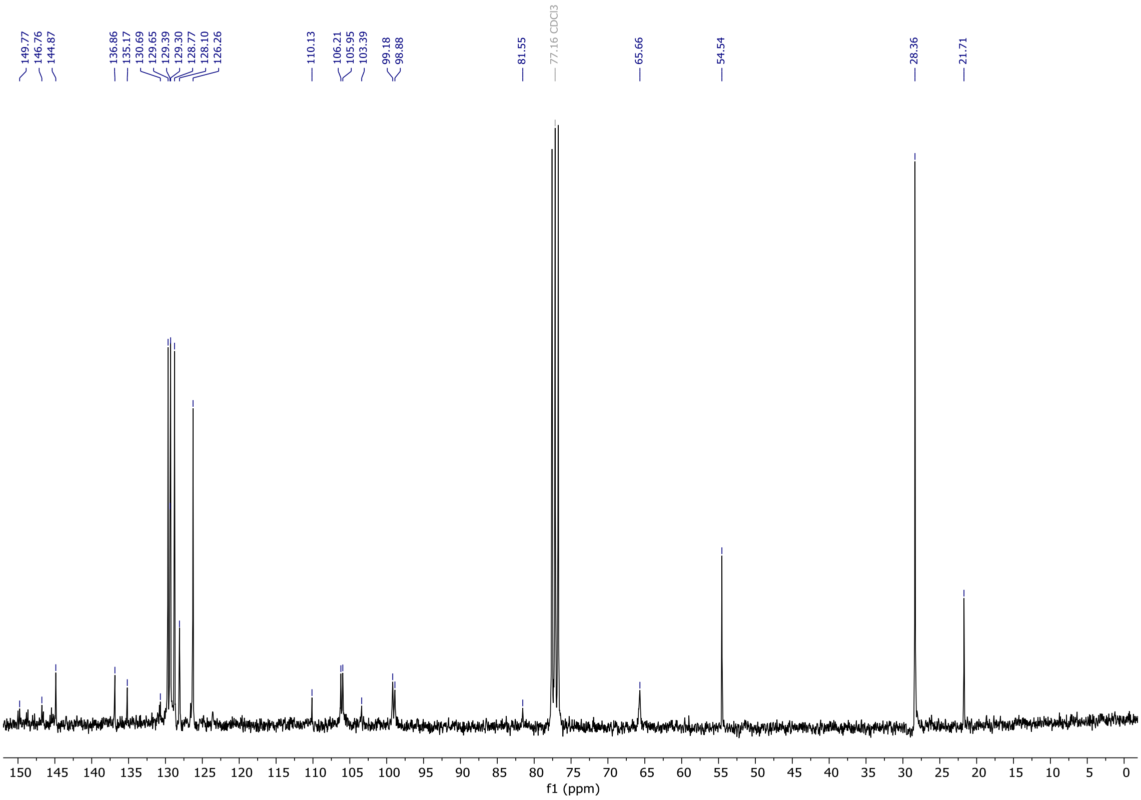

^{19}F NMR (471 MHz, $CDCl_3$) of **14aj**

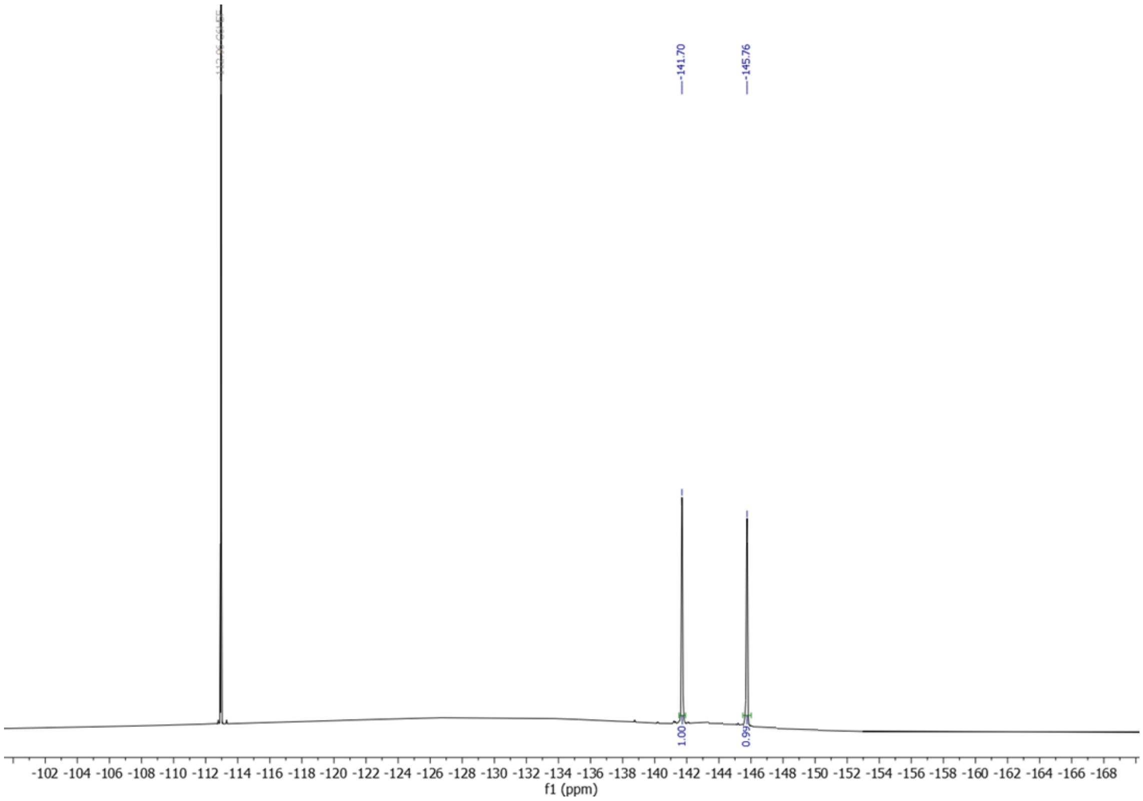

^{1}H NMR (300 MHz, $CDCl_3$) of **14al**

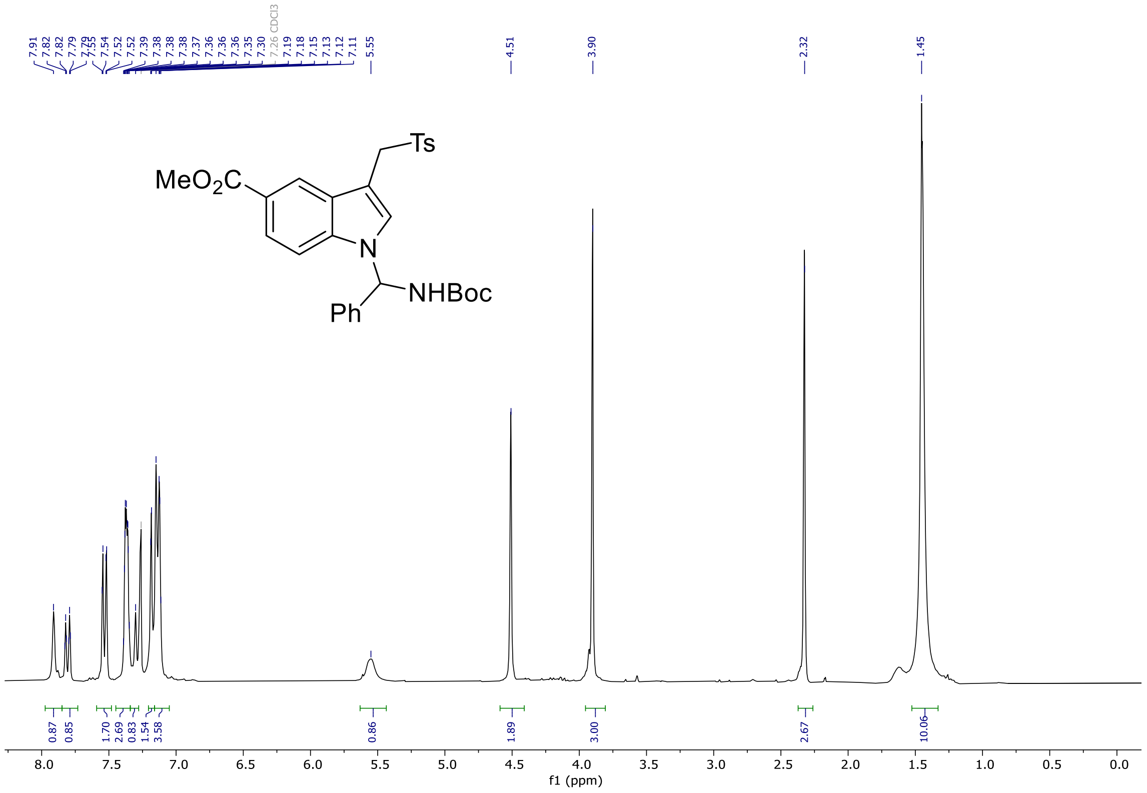

$^{13}C\{^1H\}$ NMR (75 MHz, $CDCl_3$) of **14al**

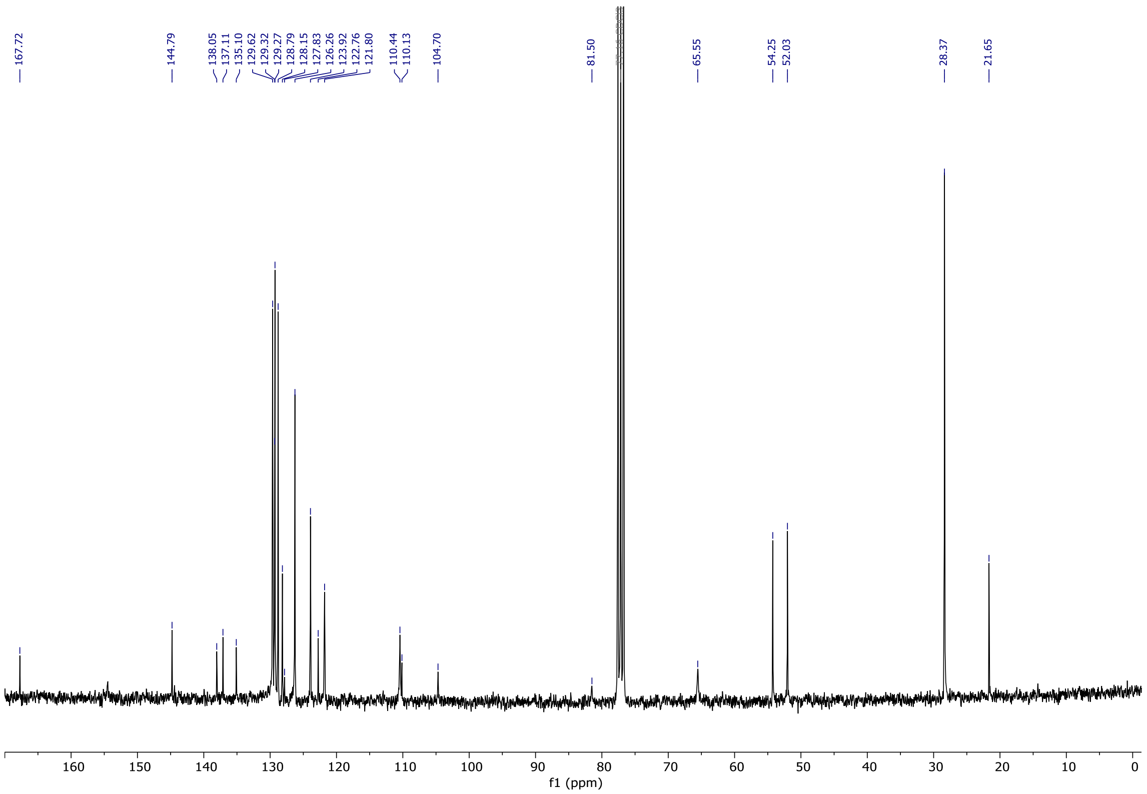

^{1}H NMR (300 MHz, $CDCl_3$) of **14am**

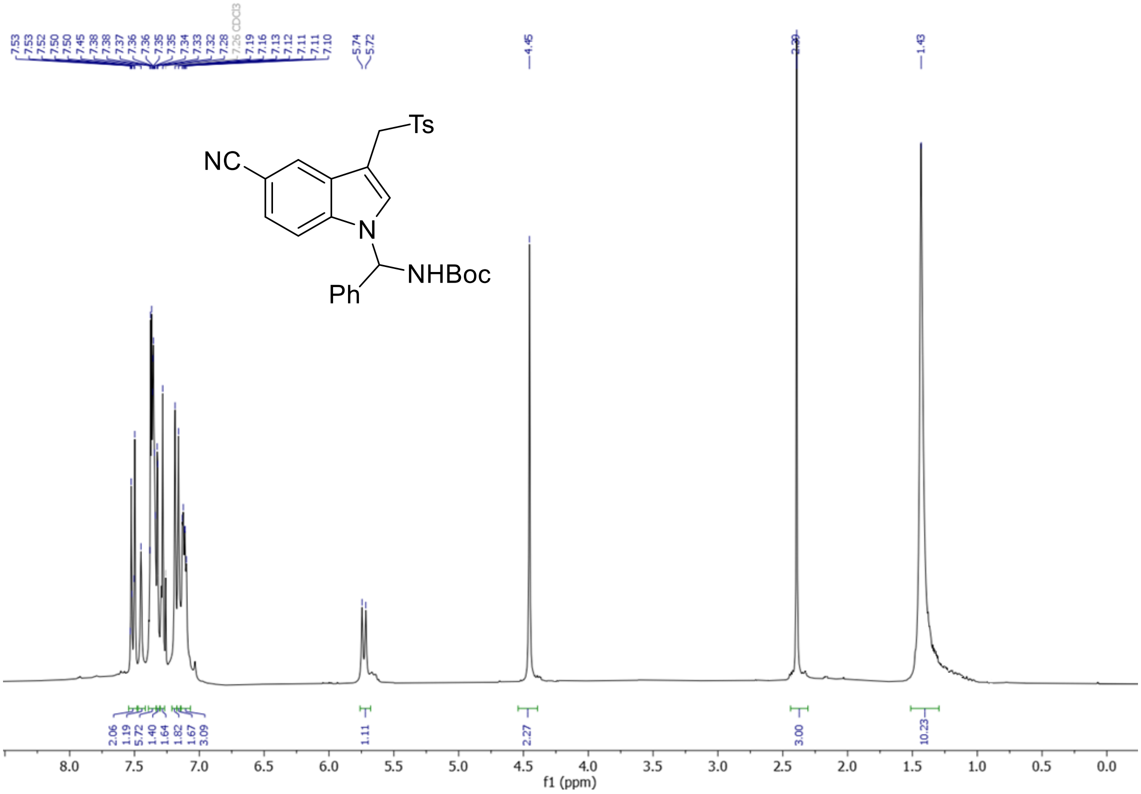

^{13}C{^{1}H} NMR (75 MHz, $CDCl_3$) of **14am**

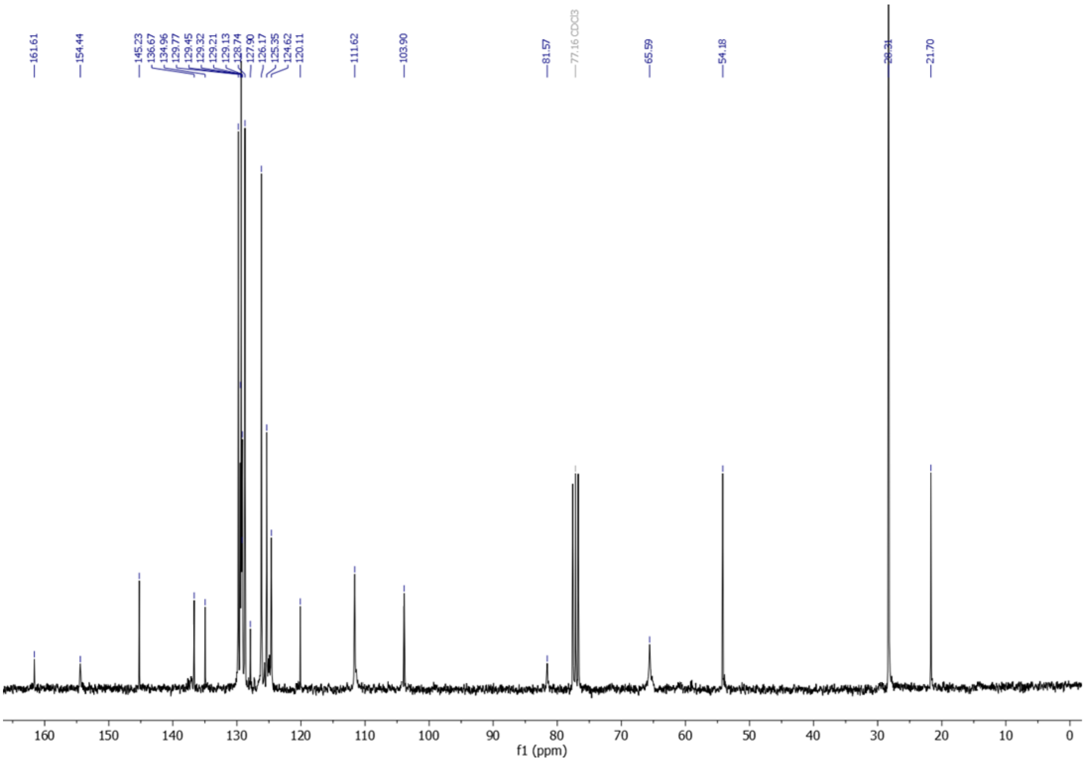

^{1}H NMR (300 MHz, Acetone-d_6) of **14an**

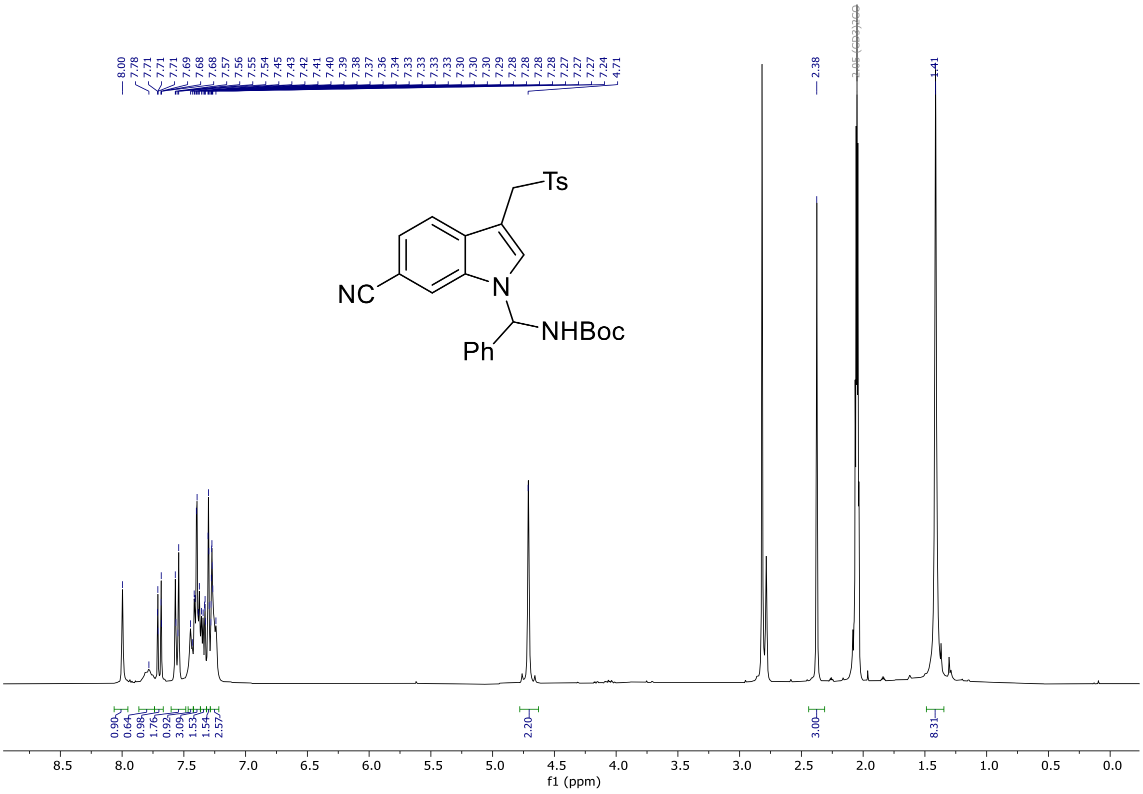

$^{13}C\{^{1}H\}$ NMR (75 MHz, Acetone-d_6) of **14an**

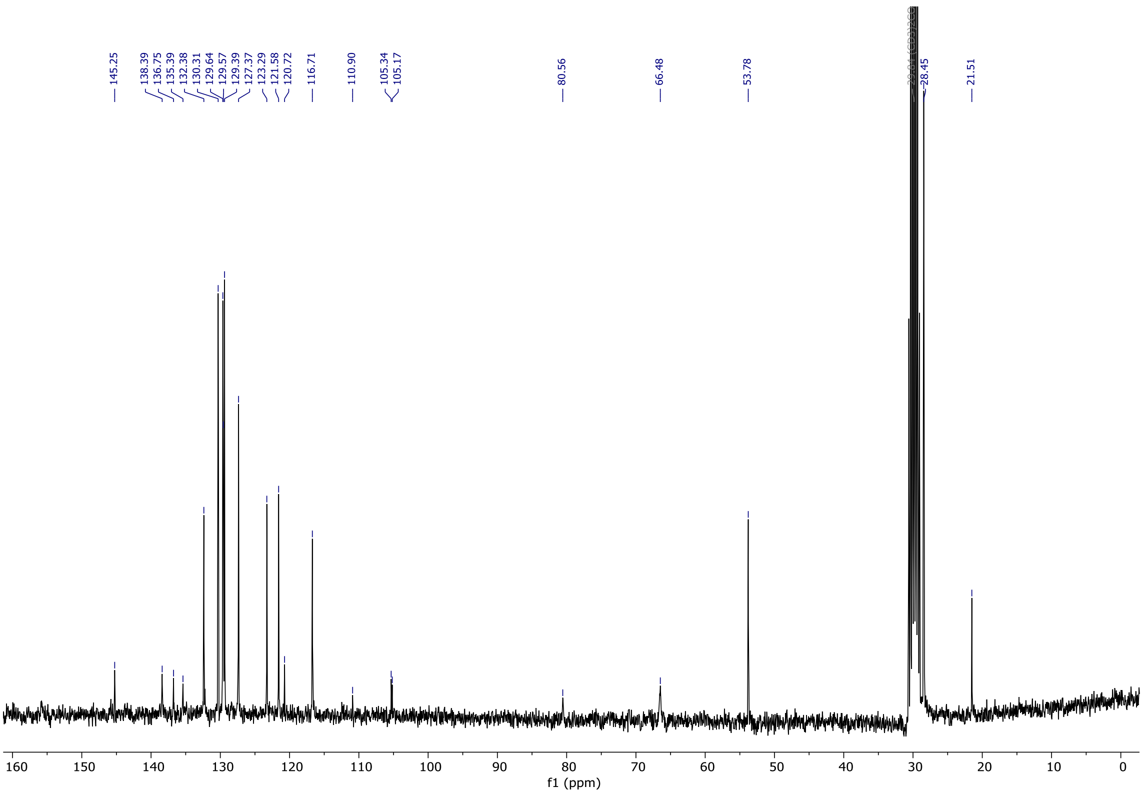

^{1}H NMR (300 MHz, $CDCl_3$) of **14ao**

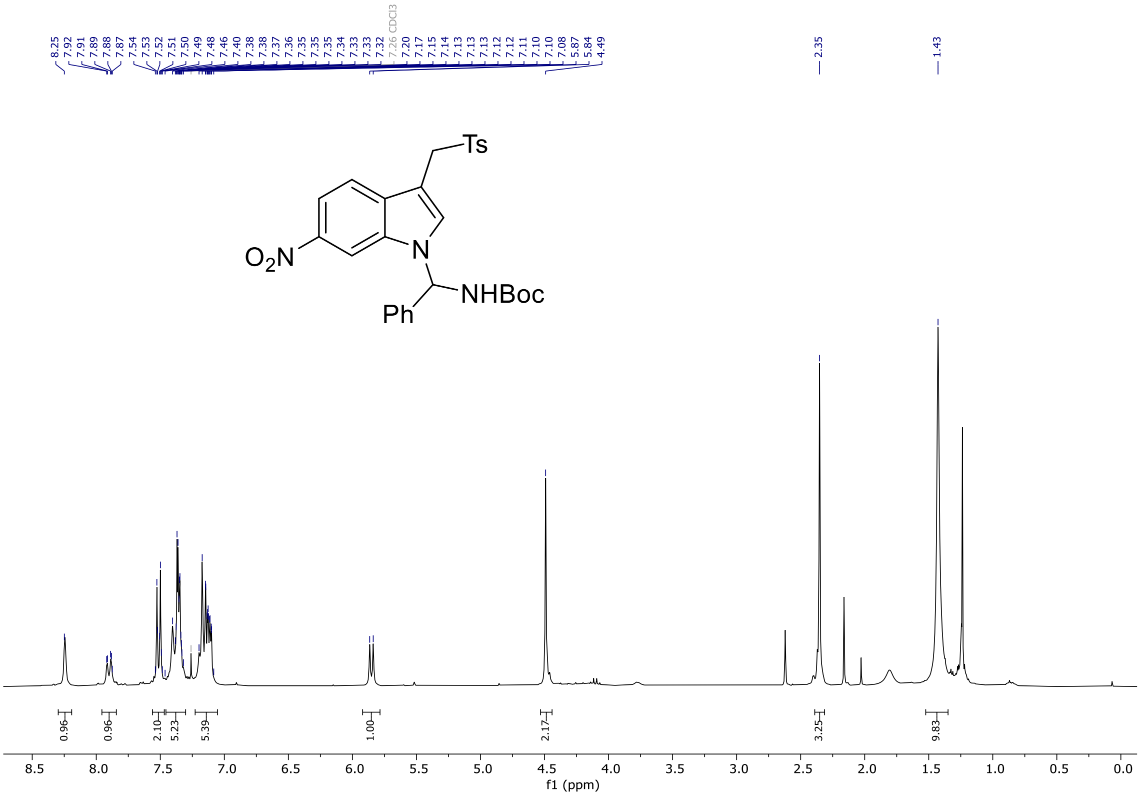

$^{13}C\{^{1}H\}$ NMR (75 MHz, $CDCl_3$) of **14ao**

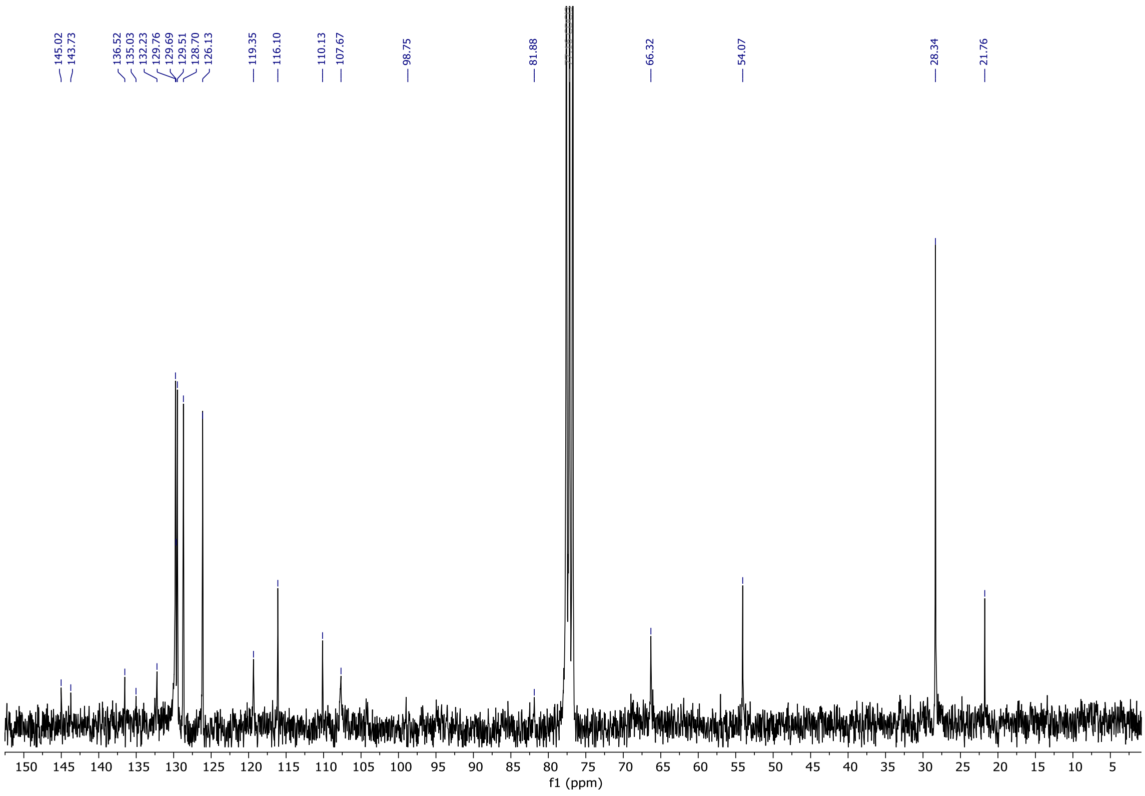

^{1}H NMR (300 MHz, $CDCl_3$) of **14dh**

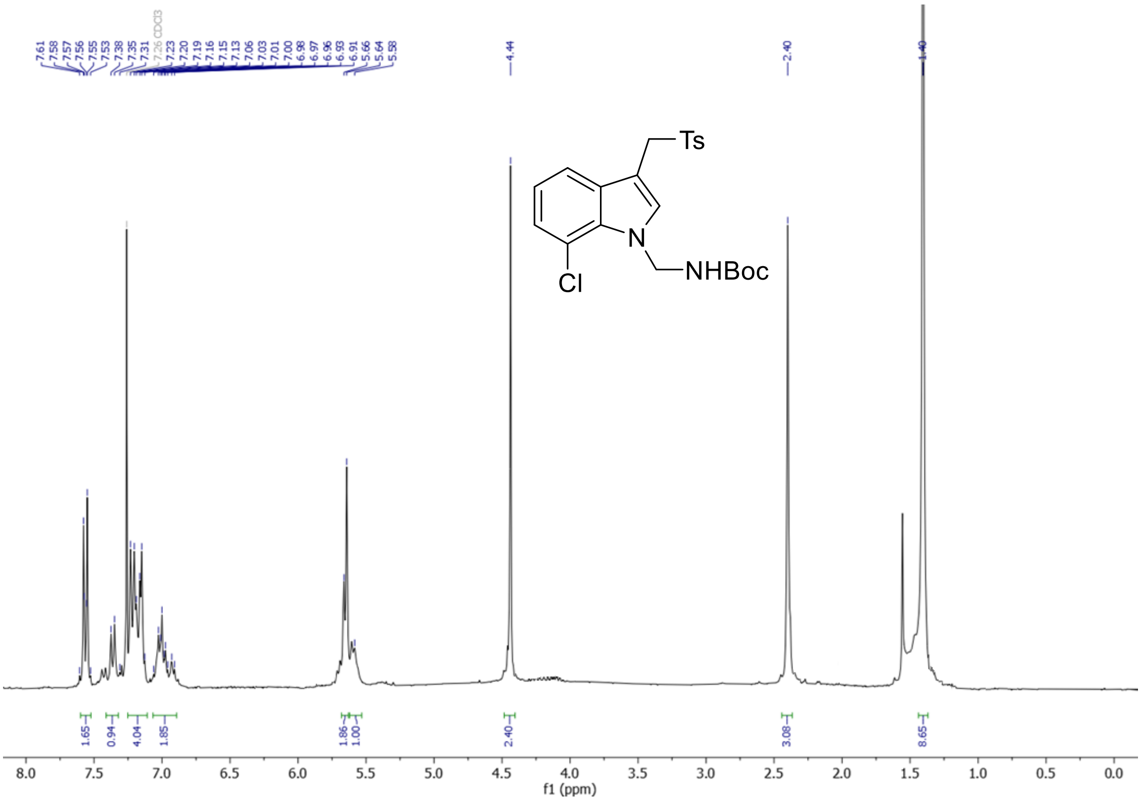

$^{13}C\{^{1}H\}$ NMR (75 MHz, $CDCl_3$) of **14dh**

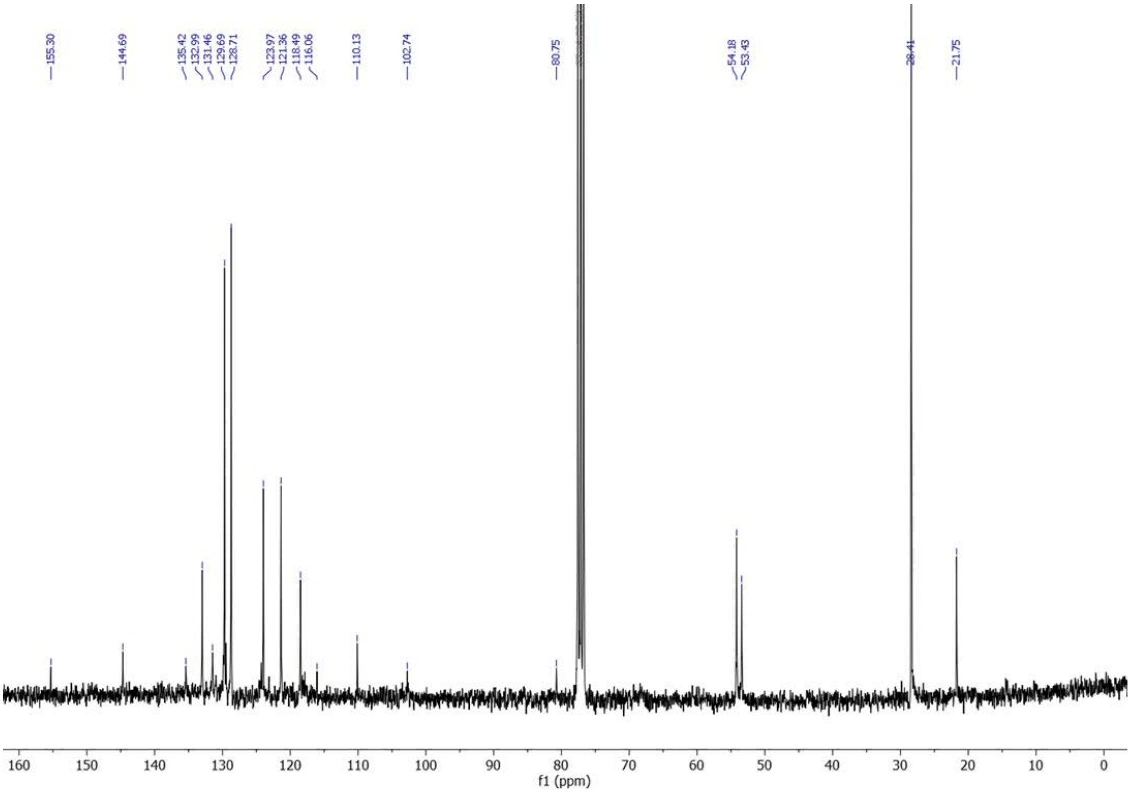

^{1}H NMR (300 MHz, $CDCl_3$) of **14ds**

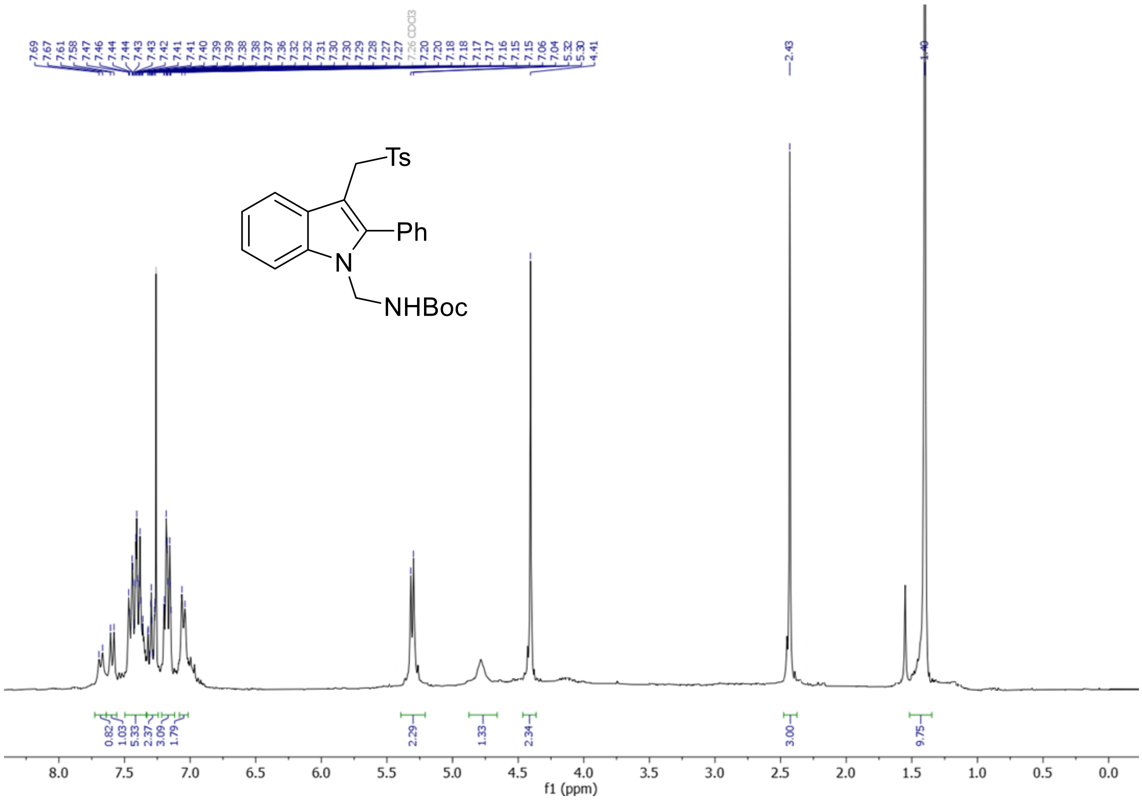

$^{13}C\{^{1}H\}$ NMR (75 MHz, $CDCl_3$) of **14ds**

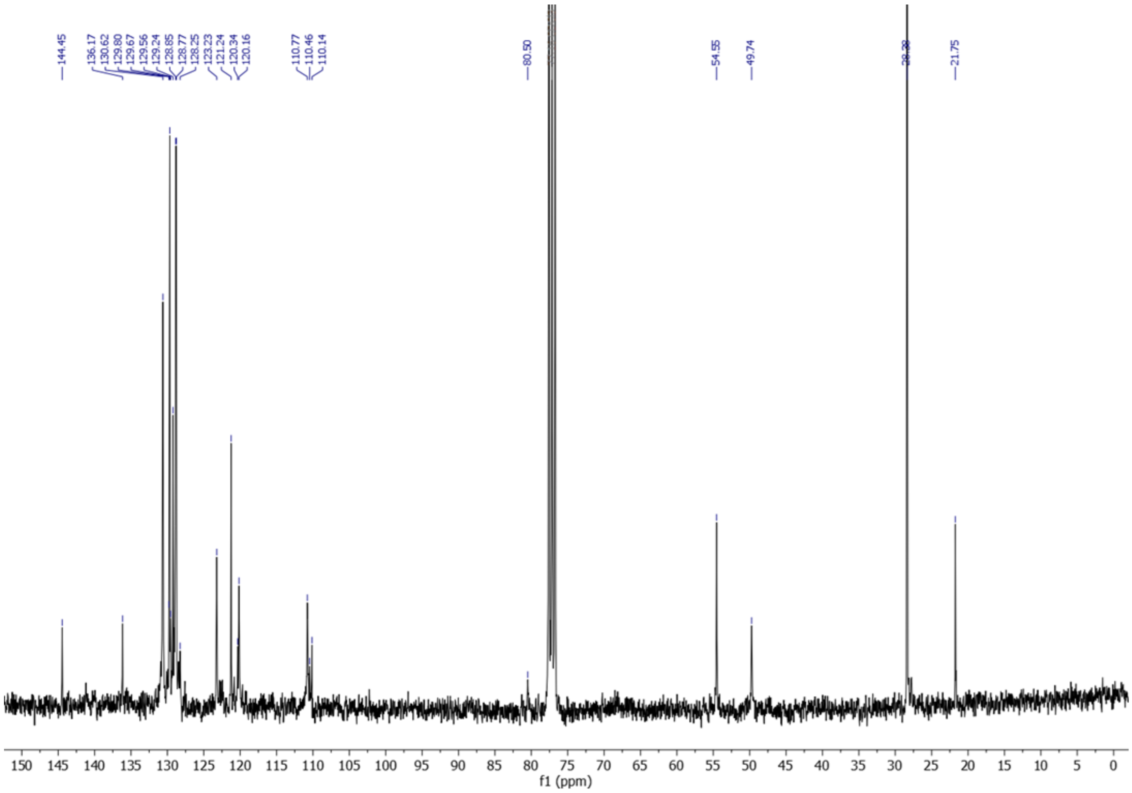

Supplement: Supplementary file 1 [file jo5c01392_si_001.pdf]
